# Phytochemical Characterization of *Tilia americana* var. *mexicana*, Exploratory Study of MAO Molecular Docking and Evaluation of Behavior in Mice in a Reserpine-Induced Parkinson Model

**DOI:** 10.3390/molecules31162874

**Published:** 2026-08-17

**Authors:** Maribel Osorio-García, Antonio Ruperto Jiménez-Aparicio, Maribel Herrera-Ruiz, Enrique Jiménez-Ferrer, Alejandro Zamilpa, Blanca Eda Domínguez-Mendoza, Gabriela Trejo-Tapia, Manasés González-Cortazar

**Affiliations:** 1 Centro de Desarrollo de Productos Bióticos, Instituto Politécnico Nacional, Carr. Yautepec-Jojutla s/n-km. 85, San Isidro, Yautepec 62739, Morelos, Mexico; mosoriog2300@alumno.ipn.mx (M.O.-G.); gttapia@ipn.mx (G.T.-T.); 2 Facultad de Ciencias Químicas e Ingeniería, Universidad Autónoma del Estado de Morelos, Av. Universidad No. 1001, Col. Chamilpa, Cuernavaca 62209, Morelos, Mexico; 3 Centro de Investigación Biomédica del Sur, Instituto Mexicano del Seguro Social, Argentina No. 1, Col. Centro, Xochitepec 62790, Morelos, Mexico; cibis_herj@yahoo.com.mx (M.H.-R.); enriqueferrer_mx@yahoo.com (E.J.-F.); azamilpa_2000@yahoo.com.mx (A.Z.); 4 Centro de Investigaciones Químicas, Universidad Autónoma del Estado de Morelos, Av. Universidad No. 1001, Col. Chamilpa, Cuernavaca 62209, Morelos, Mexico; bed@uaem.mx

**Keywords:** Parkinson’s disease, *Tilia americana*, MAO-B, molecular docking

## Abstract

*Tilia americana* var. *mexicana* is a medicinal species whose reported pharmacological effects primarily target the central nervous system, including anxiolytic, antidepressant, and anticonvulsant activities, attributed in part to flavonoids such as tiliroside (**8**). In this study, 14 compounds were isolated and identified from the methanolic extract (Ta-MeOH) and evaluated by molecular docking against the monoamine oxidases MAO-A and MAO-B, enzymes implicated in Parkinson’s disease (PD) due to their involvement in dopamine metabolism. Chemical analysis identified six terpenes: α- and β-amyrine (**1**–**2**), β-sitosterol (**3**), stigmasterol (**4**), ursolic acid (**5**), and β-sitosterol glucoside (**6**); the novel diglycosylated monoterpene 4α-terpineol sambubioside (**7**), characterized through its hexaacetate derivative (**7a**); the flavonoids tiliroside (**8**) and rutin (**9**); sucrose (**10**); and four phenolic compounds: scopoletin (**11**)**,** caffeic acid (**12**), coumaric acid (**13**), and chlorogenic acid (**14**). Molecular docking against monoamine oxidases A and B (MAO-A and MAO-B) was used as a computational strategy to prioritize isolated metabolites for biological evaluation. β-Sitosterol glucoside (**6**), 4α-terpineol sambubioside (**7**), tiliroside (**8**) and rutin (**9**) exhibited the most favorable docking scores toward MAO-B, with predicted binding energies of −10.8733, −11.1434, −12.16986 and −12.9474 kcal/mol, respectively. Considering the docking results together with phytochemical and experimental criteria, (**6**) and (**8**) (1 mg/kg) were prioritized for biological evaluation, together with Ta-MeOH extract (100 mg/kg) and selected fractions (25 mg/kg), in a reserpine-induced mouse model of parkinsonism using L-DOPA (150 mg/kg) as a positive control. In the open field test, spontaneous locomotor activity was assessed by total crossings and expressed as AUC. Ta-MeOH significantly increased locomotor activity compared with the reserpine-treated group (AUC: 1281.8 vs. 551.7, respectively; *p* < 0.05). Tiliroside (**8**) also increased locomotor activity (AUC: 858). In the Rota-Rod test, fine motor coordination was assessed by latency to fall and expressed as AUC. Ta-MeOH showed the greatest recovery, with AUC values of 431, 89, and 34 at 4, 10, and 20 rpm, respectively. In addition, treatments derived from *T. americana* attenuated behavioral alterations induced by reserpine in the Irwin test. Overall, this study demonstrates that integrating phytochemical isolation, molecular docking, and in vivo pharmacological evaluation provides a useful strategy for prioritizing bioactive metabolites from *T. americana*. These findings support further pharmacological investigation of this medicinal species and its isolated metabolites, while additional studies are required to establish their molecular targets and mechanisms of action.

## 1. Introduction

Parkinson’s disease (PD) affects 1% of the population above 60 years, and its prevalence is increasing with age [1]. It is the second most common neurodegenerative disorder worldwide. It is characterized by the progressive degeneration of dopaminergic neurons in the substantia nigra pars compacta, leading to dopamine depletion in the striatum. This neuronal loss results in the cardinal motor symptoms of PD, including tremor, rigidity, bradykinesia, and postural instability. In addition to motor impairment, patients frequently experience non-motor manifestations such as sleep disturbances, depression, and cognitive dysfunction, which significantly impact quality of life [2,3,4,5]. The pathological mechanisms underlying PD involve multiple processes, such as oxidative stress, ferroptosis, mitochondrial failure, neuroinflammation, gut dysbiosis, abnormal protein aggregation (particularly of α-synuclein), and the catalyzed degradation of dopamine by monoamine oxidases [6,7].

Monoamine oxidases (MAOs) are flavin-containing enzymes located on the outer mitochondrial membrane that catalyze the oxidative deamination of monoamine neurotransmitters such as dopamine, serotonin, and norepinephrine. Two isoforms of this enzyme exist, MAO-A and MAO-B, which differ in substrate specificity, tissue distribution, and pharmacological inhibition. MAO-A preferentially metabolizes serotonin and norepinephrine, whereas MAO-B primarily metabolizes dopamine and trace amines [8].

In the context of PD, MAO-B plays a particularly relevant role because dopamine metabolism through this enzyme produces hydrogen peroxide and other reactive oxygen species, thereby contributing to oxidative stress and neuronal damage [9,10]. Inhibition of MAO-B has therefore emerged as an important therapeutic strategy for Parkinson’s disease, as it reduces dopamine degradation and helps maintain dopaminergic neurotransmission in the brain. Clinically approved MAO-B inhibitors such as selegiline, rasagiline, and safinamide are widely used either as monotherapy in early stages of PD or as adjunct therapy with levodopa in advanced disease [11,12,13]. Despite their clinical benefits, these drugs may cause adverse effects with prolonged treatment and do not halt disease progression, underscoring the need to identify novel inhibitors with improved safety profiles and potential neuroprotective properties [14,15,16].

Natural products represent an important source of bioactive molecules for the discovery of new neuroprotective agents. Plant secondary metabolites such as flavonoids, alkaloids, terpenoids, and phenolic compounds have been widely investigated for their antioxidant, anti-inflammatory, and enzyme-modulating properties in neurodegenerative diseases [17,18].

The genus *Tilia* has long been used in traditional medicine due to its effects on the central nervous system. Species such as *T. cordata*, *T. platyphyllos*, and *T. tomentosa* are traditionally employed as sedative, tranquilizing, anticonvulsant, and anxiolytic remedies. Infusions prepared from their flowers and leaves are commonly used to alleviate nervous disorders, insomnia, headaches, and gastrointestinal complaints [19,20]. *Tilia americana* var. *mexicana* (Schltdl.) Hardin (Malvaceae, subfamily Tilioideae) is widely distributed in Mexico and is locally known as “cyrimbo”, “flor de tila”, “sirima”, “tirimo”, “jonote” or “tilio” [21]. In Mexican traditional medicine, infusions prepared from its leaves or inflorescences are used to treat nervous disorders, reduce agitation, promote sleep, and relieve headaches [22,23]. Experimental studies have confirmed several central nervous system activities associated with this species, including anxiolytic, sedative, and antidepressant effects in animal models [24,25,26]. Phytochemical investigations of *Tilia* species have identified flavonoids as the major bioactive constituents responsible for many of their pharmacological properties. Compounds such as tiliroside, rutin, quercetin derivatives, and kaempferol glycosides have been reported in extracts of *T. americana* and related species [27,28]. Given the pharmacological relevance of *T. americana* var. *mexicana* and the importance of monoamine oxidase B (MAO-B) as a therapeutic target in Parkinson’s disease, the identification of natural compounds capable of modulating this enzyme represents a promising strategy for the development of novel neuroprotective agents.

Flavonoids are known to exert multiple neuroprotective effects, including antioxidant activity, modulation of neurotransmitter systems, and inhibition of enzymes involved in neurodegeneration, suggesting that some of these metabolites may interact with MAO enzymes [29,30,31].

Furthermore, in recent years, structure-based computational approaches, such as molecular docking, have become powerful tools in early drug discovery, enabling prediction of ligand–target interactions and facilitating the identification of potential MAO inhibitors from natural sources [32,33,34].

The aim of this study was to chemically characterize the methanolic extract (Ta-MeOH) of *Tilia americana* using chromatographic and spectrometric techniques. Furthermore, an in silico pharmacological study was performed by evaluating the docking scores of the isolated compounds within the MAO-A and MAO-B binding sites. Finally, the effects of the extract, fractions, and selected compounds were evaluated in a murine model of reserpine-induced parkinsonism through behavioral and motor assessments.

## 2. Results

### 2.1. Isolation and Structural Elucidation and Chemical Classification of Compounds from Methanolic Extract of Leaves of Tilia americana var. mexicana

**Figure 1:**
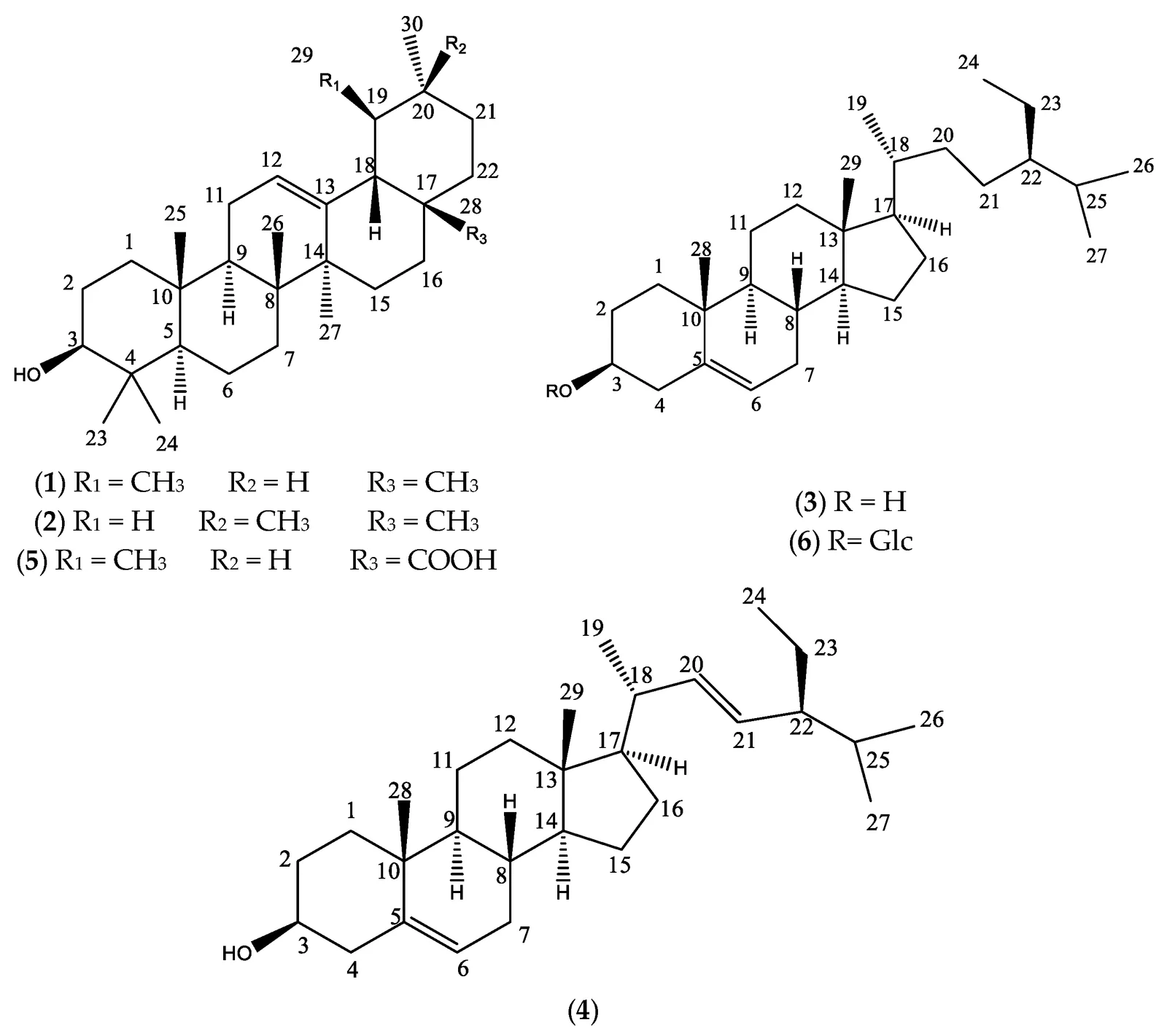
Triterpene-type compounds (**1**–**6**) from leaves of *T. americana* var. *mexicana.*

The extraction yields from leaves of T. americana were 0.74% for the hexane extract (Ta-Hex) and 8.33% for the methanolic extract (Ta-MeOH). After fractionation of Ta-MeOH, five groups of fractions were obtained, designated Ta-R1 to Ta-R5. TLC analysis indicated that Ta-R1 and Ta-R2 contained mainly terpenoid compounds, whereas Ta-R3 to Ta-R5 contained flavonoids and phenolic compounds and saccharides. In this study, several known triterpene-type compounds were obtained: α- and β-amyrine (**1**) and (**2**), β-sitosterol (**3**), stigmasterol (**4**), ursolic acid (**5**), and β-sitosterol glucoside (**6**) (Figure 1).

Compounds (**1**) and (**2**) were obtained as a colorless powder mixture that was soluble in CH_2_Cl_2_. The compounds were visualized on TLC using 4-hydroxybenzaldehyde as the visualizing reagent. The δ 0.67–1.90 region of the ^1^H NMR spectrum displayed a typical triterpene pattern, with H-3 appearing at δ 3.15 (ddd, *J* = 5.5, 11.9, 17.8 Hz), indicating a β-oriented hydroxyl group. The presence of both α-amyrine and β-amyrine was confirmed by the vinyl proton signals of H-12 at δ 5.06 (dd, *J* = 3.6, 3.8 Hz) and 5.12 (dd, *J* = 3.4, 3.5 Hz). The ^13^C NMR spectrum (Supplementary Data S2) displayed 30 carbon signals with the characteristic vinyl carbons of α-amyrine δ 124.6 (C-12); 139.7 (C-13), and β-amyrine δ 121.9 (C-12), 145.3 (C-13). These results were consistent with previous reports, confirming the identification of α-amyrine (**1**) and β-amyrine (**2**) [35,36]. The molecular formula was determined to be C_30_H_50_O, with a calculated molecular weight of 426.39 g/mol. In the negative ion ESI-MS (Supplementary Data S3) showed ions at *m*/*z* 425 [M-H]^−^ for the molecular formula C_30_H_49_O and *m*/*z* 448 [M+Na−H]^−^ for the molecular formula C_30_H_49_NaO.

Compounds (**3**) and (**4**) were obtained as a white solid mixture of β-sitosterol and stigmasterol, soluble in CH_2_Cl_2_. The ^1^H NMR spectrum (Supplementary Data S4) showed a typical steroidal profile (δ 0.68–2.37), with H-3 at δ 3.53 (ddd, *J* = 5.5, 11.9, 17.8 Hz), indicating a β-oriented hydroxyl group. Vinyl protons of stigmasterol were observed at δ 5.02 (dd, *J* = 8.6, 15.2 Hz, H-20) and 5.15 (dd, *J* = 8.6, 15.2 Hz, H-21), and the olefinic H-6 at δ 5.35 (d, *J* = 4.9 Hz). The ^13^C NMR spectrum (Supplementary Data S5) displayed signals with the characteristic vinyl carbons of β-sitosterol δ 140.9 (C-5), 121.9 (C-6) and stigmasterol δ 140.9 (C-5), 121.9 (C-6), 138.5 (C-20), 129.4 (C-21). Spectroscopic data were consistent with literature reports for β-sitosterol and stigmasterol [37,38]. The molecular formulas corresponding to β-sitosterol and stigmasterol are C_29_H_50_O (MW 414.39), and C_29_H_48_O (MW 412.37), respectively. In the positive ion ESI-MS (Supplementary Data S6) showed ions at *m*/*z* 414 [M]^+^ and *m*/*z* 412 [M]^+^.

Compound (**5**) was obtained as a white amorphous powder, soluble in CH_2_Cl_2_. The ^1^H NMR spectrum (Supplementary Data S7) exhibited characteristic signals for a pentacyclic triterpene skeleton, including a broad singlet at δ 5.22 corresponding to the olefinic proton H-12 and a signal at δ 3.16 assigned to H-3, indicating a hydroxyl group at C-3. The ^13^C NMR spectrum (Supplementary Data S8) showed signals consistent with a ursane-type triterpene, including a carboxylic carbon at δ 177.6 and olefinic carbons at δ 125.3 and 138.4. The spectroscopic data were consistent with the literature values for ursolic acid [39]. Its molecular formula was established as C_30_H_48_O_3_ (calculated molecular weight 456.36 g/mol). In the negative ion ESI-MS (Supplementary Data S9) showed a deprotonated molecular ion at *m*/*z* 455 [M-H]^−^ for the molecular formula C_30_H_47_O_3_.

Compound (**6**) was obtained as a white amorphous powder, soluble in DMSO and in a CH_2_Cl_2_/methanol mixture. In the ^1^H NMR spectrum (Supplementary Data S10), the aglycone signals were consistent with those of previously described β-sitosterol. Additionally, the classical signal for a sugar moiety, the anomeric proton consistent with glucose, was detected at δ 4.21 as a doublet (*J* = 7.9 Hz). The ^13^C NMR spectrum (Supplementary Data S11) displayed thirty signals corresponding to the triterpene skeleton, along with additional signals attributable to the glucose moiety. A signal corresponding to anomeric carbon C-1′ was observed at δ 100.8. The spectroscopic data were consistent with literature reports for β-sitosterol glucoside [40]. Its molecular formula was established as C_35_H_60_O_6_ (calculated molecular weight 576.85 g/mol). In the positive ion ESI-MS (Supplementary Data S12) showed ion at *m*/*z* 577 [M+H]^+^ for the molecular formula C_35_H_61_O_6_.

**Figure 2:**
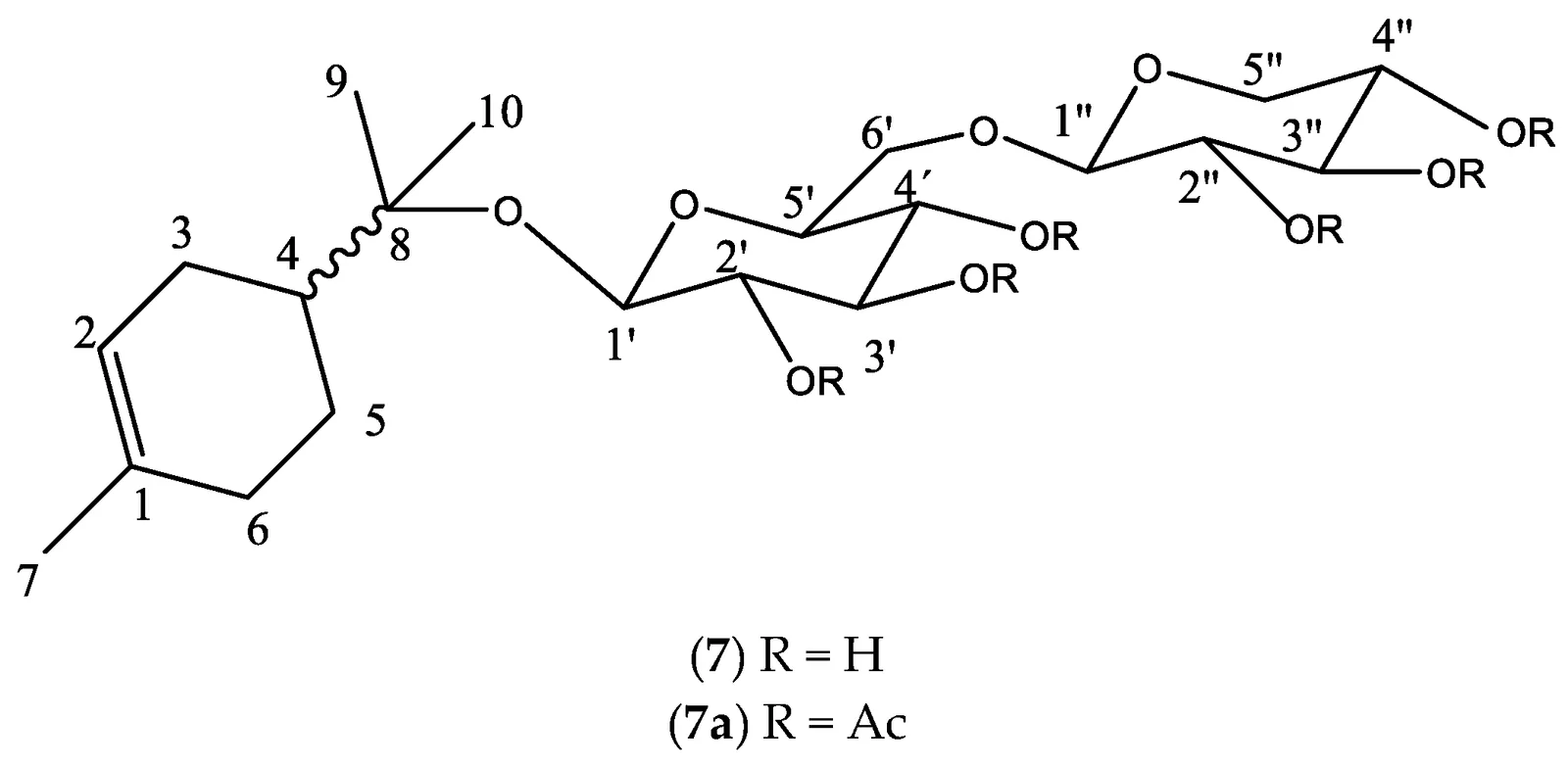
New compound 4α-terpineol sambubioside (**7**) and its hexaacetylated derivative 4α-terpineol sambubioside hexaacetate (**7a**) from leaves of *T. americana*.

In addition, a novel compound (**7**), identified as a diglycosylated monoterpene, was obtained and characterized as its hexaacetylated derivative, 4α-terpineol sambubioside hexaacetate (**7a**) (Figure 2).

**Figure 3:**
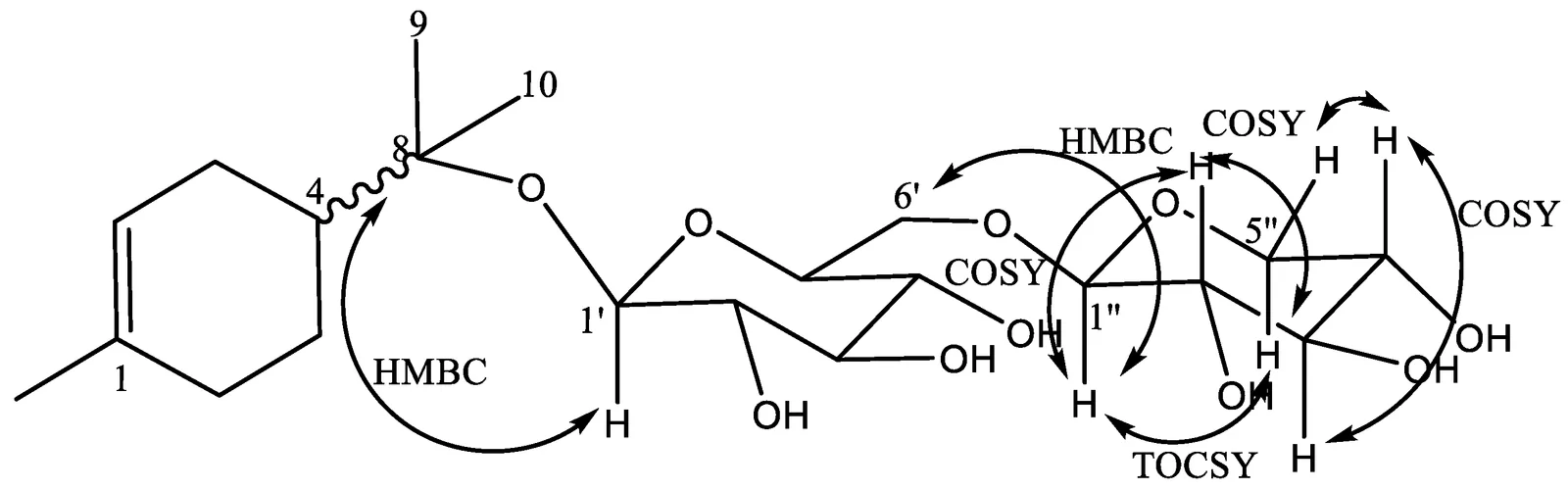
COSY, TOCSY, and HMBC correlations of (**7a**).

The ^1^H NMR spectrum of compound (**7a**), obtained as a white solid soluble in CH_2_Cl_2_, showed two characteristic double signals for the anomeric hydrogens at δ 4.66 (d, *J* = 8.0 Hz, H-1′) and at δ 4.55 (d, *J* = 6.5 Hz, H-1″), respectively (Supplementary Data S13). This is consistent with the DEPT and HSQC experiments (Supplementary Data S14, Supplementary Data S17) in which signals were observed at δ 95.2 and 100.17 for C-1′ and C-1″ respectively [41]. Analysis of the TOCSY experiment (Supplementary Data S15) revealed correlations for the anomeric proton H-1′ with the hydrogens H-2′, H-3′, H-4′, H-5′, H-6a′, and H-6b′ for a hexose molecule. The COSY experiment showed the correlation of H-1′ at δ 4.66 (d, *J* = 8.0 Hz) with the multiplet signal at δ 4.90 (m) assigned to H-2′, which in turn correlates with H-1′ and with the triplet signal at δ 5.19 (*J* = 9.5 Hz) assigned to H-3′. This analysis was performed for each hydrogen in this hexose, determining its coupling constants and chemical shift, thereby allowing for the identification of this sugar as β-glucose. The same analysis was followed for the second sugar. The TOCSY experiment showed all the correlations of H-1″ with H-2″, H-3″, H-4″, H-5a″, and H-5b″, indicating a pentose-type molecule. COSY experiment of H-1″ at 4.55 (d, *J* = 6.5 Hz) showed coupling with a double–double signal at δ 4.87 (dd, *J* = 8.3, 6.5 Hz) assigned to H-2″, likewise H-2″ showed coupling with the double–double signal at δ 5.10 (dd, *J* = 8.1, 8.2 Hz) assigned to H-3″. H-3″ couples with a multiplet signal at δ 4.91 (m) and H-4″ couples with the H4″ signal with H-5a″ and H-5b″ at δ 4.13 (dd, *J* = 11.9, 4.9 Hz, H-5a″) and 3.34 (dd, *J* = 12.0, 8.4 Hz, H-5b″) along with geminal coupling (*J* = 12 Hz) and the axial–axial coupling (*J* = 8.4 Hz) between H-5b″ and H-4″, together with the axial–equatorial coupling (*J* = 4.9 Hz) between H-5a″ and H-4″, these data allowed for us to determine that this sugar is called β-xylose. The HSQC experiment (Supplementary Data S17) was used to determine and assign the chemical shifts of each carbon to their corresponding hydrogen atoms. The interglycosidic bond was observed in the HMBC (Supplementary Data S18), with three H-1″(δ 4.55) ligations of xylose to C-6′(δ 67.65) of glucose and glucose to H-1′(4.66) with an oxygen base signal corresponding to C-8(δ 80.62). See all correlations in Figure 3.

On the other hand, in the DEPT spectrum, 10 remaining signals were observed, corresponding to three methyl (δ 23.47, 22.04, and 25.22), three methylene (δ 26.79, 23.56, and 31.0), one methine (δ 44.12), one quaternary (δ 80.62), and two olefinic (δ 134.22 and 120.44) carbons, consistent with a monoterpene fragment for the aglycone skeleton known as 4α-terpineol. Based on these spectroscopic data (see Table 1) and comparisons with the literature for a similar compound [41], (**7a**) was identified as 4α-terpineol 8-O-[β-xylopyranosyl-(1→6)-β-glucopyranoside] hexaacetate (**7a**)**,** known as sambubioside of 4α-terpineol sambubioside hexaacetate, and the natural product would be 4α-terpineol sambubioside (**7**).

The molecular formula of 4α-terpineol sambubioside hexaacetate (**7a**) was established as C_33_H_48_O_16_ (calculated molecular weight 700.29 g/mol). In the positive ion, ESI-MS (Supplementary Data S19) showed a molecular ion at *m*/*z* 723.27 [M+Na]^+^ for C_33_H_48_NaO_16_, and *m*/*z* 137 [M-acetilated sambubioside (C_23_H_31_O_16_)]^+^ (Supplementary Data S20) and HR-ESI-MS analysis revealed the molecular formula to be C_33_H_48_NaO_16_ (*m*/*z* = 723.2820 [M+Na]^+^); see Supplementary Data S21.

**Figure 4:**
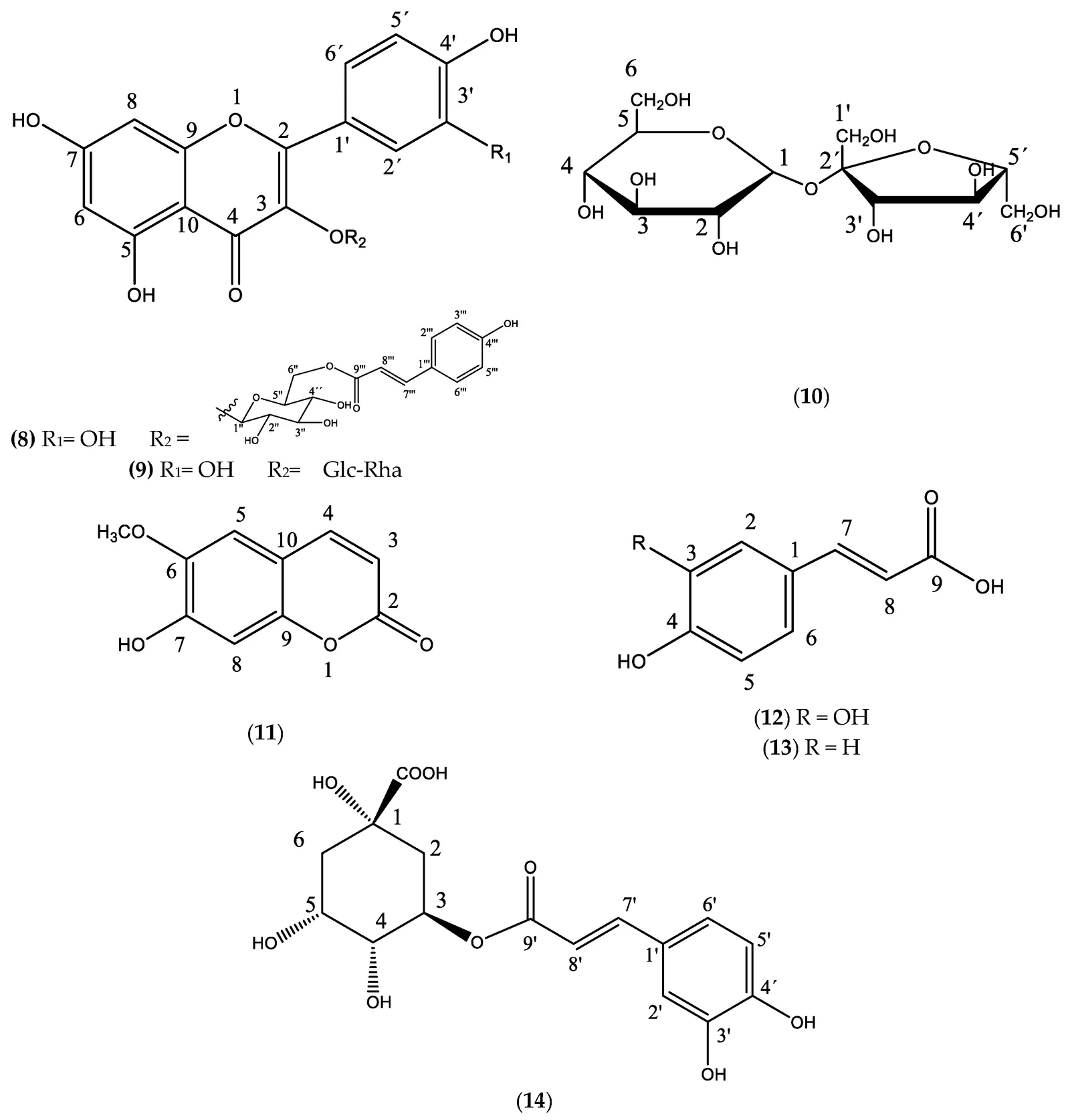
Flavonoids (**8**–**9**), sugars (**10**) and phenolic compounds (**11**–**14**) are isolated from the methanolic extract of the leaves of *T. americana.*

In addition to the compounds described above, the flavonoids tiliroside (**8**) and rutin (**9**) were identified. Sucrose (**10**) and four phenolic compounds, scopoletin (**11**), caffeic acid (**12**), coumaric acid (**13**), and chlorogenic acid (**14**), were detected too (Figure 4).

Compound (**8**) was obtained as a yellow amorphous powder. UV (MeOH) λ_max_ 213.1, 266.3 and 313.9 nm. The ^1^H NMR spectrum (Supplementary Data S22), exhibited three characteristic subsystems: (i) a kaempferol nucleus, evidenced by two meta-coupled doublets at δ 6.10 and 6.25 (*J* = 2.5 Hz, H-6 and H-8) and an AA′BB′ aromatic system at δ 7.97 and 6.79 (*J* = 8.9 Hz, H-2′, H-6′/H-3′, H-5′); (ii) on the other hand, a *p*-coumaroyl is present, indicated by an aromatic ring system AA′BB′ at δ 7.29 and 6.78 *(J* = 8.5 Hz) together with trans-olefinic protons (H-7‴-H-8‴) at δ 6.07 and 7.38 (*J* = 15.6 Hz), confirming an *E*-configuration; and (iii) a sugar unit identified as the β-glucopyranosyl by the presence of the anomeric proton at δ 5.24 (d, *J* = 7.6 Hz). The DEPT spectra (Supplementary Data S23) revealed 12 quaternary carbons, 17 methine carbons, and two methylenes, consistent with a flavonol glycoside bearing an acyl substituent. HSQC correlations (Supplementary Data S24) established glycosylation at C-3 of the flavonol core and acylation of C-6″ of glucose by the *p*-coumaroyl group. Spectroscopic data were consistent with literature reports for the following compound, its IUPAC name being [(2R,3S,4S,5R,6S)-6-[5,7-dihydroxy-2-(4-hydroxyphenyl)-4-oxochromen-3-yl]oxy-3,4,5-trihydroxyoxan-2-yl]methyl (E)-3-(4-hydroxyphenyl)prop-2-enoate, known as kaempferol-3-O-(6″-O-cis-coumaryl)glucoside or tiliroside [42,43]. Its molecular formula was established as C_30_H_26_O_13_ the molecular weight of 594.5 g/mol was confirmed by positive mode ESI-MS (Supplementary Data S28). In the positive ion, ESI-MS showed molecular ions at *m*/*z* 595 [M+H]^+^ for the molecular formula C_30_H_27_O_13_ and 617 [M+Na]^+^ for the molecular formula C_30_H_26_NaO_16_. Together, these data confirmed compound (**8**) as tiliroside. This compound had already been isolated and identified in this species.

Compound (**9**) was obtained as a yellow amorphous powder, soluble in methanol. UV (MeOH) λ_max_ 211.9, 255.6 and 353.4 nm. The ^1^H NMR spectrum (Supplementary Data S29) exhibited characteristic signals a quercetin nucleus conformed by an AB system, H-6 at δ 6.20 (d, *J* = 2.3 Hz) and H-8 at δ 6.39 (d, *J* = 2.3 Hz), and an additional ABX system with H-2′ at δ 7.67 (d, *J* = 2.3 Hz), H-5′ at δ 6.86 (d, *J* = 8.5 Hz), H-6′ at δ 7.63 (dd, *J* = 2,3, 8.5 Hz). Additionally, a rutinose moiety, with two anomeric protons at δ 5.11 (d, *J* = 7.4 Hz, H-1″) and 4.52 (d, *J* = 1.5 Hz, H-1‴), indicating β-glucose and α-rhamnose configurations, and for the latter one δ 1.12 (d, *J* = 6.2) corresponding to the hydrogens of the methyl group of rhamnose (H-6‴). The ^13^C NMR spectrum (Supplementary Data S30) show signals of AB system at δ 134.2 (C-3), 177.9 (C-4), 157.9 (C-5), 98.6 (C-6), 164.9 (C-7), 93.5 (C-8), and 161.5 (C-9), and signals of ABX system at δ 122.5 (C-1′), 116.3 (C-2′), 144.4 (C-3′), 148.4 (C-4′), 116.3 (C-5′), 122.5 (C-6′). The anomeric carbons are present at δ 101.0 (C-1″) for glucose and δ 103.2 (C-1‴) for rhamnose, and their oxygen-bearing carbons were found between δ 76.7 and 68.3, and finally at δ 16.3 the carbon corresponding to the C-6‴ of rhamnose. These data are consistent with the data described in the literature [44], and the compound was identified as quercetin 3-O-rutinoside, known as rutin. In the negative ion ESI-MS of (**9)**, the quasimolecular ion peak at *m*/*z* 609.22 [M-H]^-^ for the molecular formula C_27_H_29_O_16_ (Supplementary Data S31).

Compound (**10**), a white solid, soluble in water and DMSO, was isolated as a slightly orange amorphous solid. The ^1^H NMR spectrum (Supplementary Data S32) showed signals between δ 3.11 and 5.17, consistent with a disaccharide composed of glucose and fructose units linked through a C-1→C-2 glycosidic bond. The ^13^C NMR spectrum (Supplementary Data S33) displayed 12 oxygenated carbons between δ 60.5–104.1. Its molecular formula was established as C_12_H_22_O_11_, MW= 342.3 g/mol. In the negative ion, ESI-MS (Supplementary Data S34) showed ions at *m*/*z* 341 [M-H]^-^, *m*/*z* 325 [M-OH]^-^ and *m*/*z* 311 [M-CH_2_OH]^-^, this compound was identified as sucrose (**10**) according to data reported in the literature [45].

Compound (**11**) was isolated as a white crystalline solid, soluble in CH_2_Cl_2_. UV (MeOH): λ_max_ 224, 297 and 344 nm, characteristic of a coumarin. The spectroscopic data were consistent with literature reports [46] for 7-hydroxy-6-methoxychromen-2-one or scopoletin (**11**). In the positive ion, ESI-MS (Supplementary Data S35) showed an ion at *m*/*z* 193 [M+H]^+^ for the molecular formula C_10_H_9_O_4_.

Compound (**12**) was obtained as a yellow solid, soluble in CH_2_Cl_2_. UV (MeOH): λ_max_ 217.8, 235.5 and 322.6 nm. In the positive ion, ESI-MS (Supplementary Data S36) showed an ion at *m*/*z* 180 [M]^+^ for the molecular formula C_9_H_8_O_4_. The spectroscopic data were consistent with literature reports [47] for (*E*)-3-(3,4-dihydroxyphenyl) prop-2-enoic acid, known as caffeic acid (**12**).

Compound (**13**) was isolated as a white solid soluble in CH_2_Cl_2_. UV (MeOH): λ_max_ 226.1 and 309.2 nm. The molecular formula C_9_H_8_O_3_ and molecular weight 164.2 g/mol was confirmed with the negative ion ESI-MS (Supplementary Data S37) that showed *m*/*z* 163 [M-H]^-^ for the molecular formula C_9_H_7_O_3_. The compound was identified after comparison with reported literature data [48] as (*E*)-3-(4-hydroxyphenyl) prop-2-enoic acid or *p*-coumaric acid **(13)**.

Compound (**14**) was obtained as a yellow amorphous powder, soluble in methanol. UV (MeOH): λ_max_ 217.8, 240.2, 325.9 nm. The ^1^H NMR spectrum (Supplementary Data S38) exhibited characteristic signals for a caffeoylquinic acid. The caffeoyl moiety showed signals at δ 7.92 (d, *J*= 15.9, H- 7′), 7.54 (d, *J* = 2.0 Hz, H-2′), 7.48 (dd, *J* = 8.2, 2.0 Hz, H-6′), 7.27 (d, *J* = 8.1 Hz, H-5′), and 6.65 (d, 15.9, H-8′), and the hydrogens corresponding to the quinic moiety were observed at δ 4.43 (m, H-5), 3.96 (m, H-4) 2.28 (dd, *J*= 7.6, 13.2, H-6 ax), 2.52 (dd, *J*= 12.2, 5.9 Hz, H-6 eq), 2.44 (d, *J* = 3.7, H-2ax), and 2.46 (d, *J* = 2.3 Hz, H-2eq). The ^13^C NMR spectrum (Supplementary Data S39) showed signals at δ 175.12 (COOH) and 165.96 (C=O) caffeic carbonyl; δ 148.50 (C-4′), 145.7 (C-7′), 145.1 (C-5′), 125.7 (C- 1)′, 121.6 (C-2′), 115.9 (C-3′), 114.8 (C-8′), 114.4 (C-6′), 73.6 (C-1), 71.0 (C-4), 70.4 (C-3), 68.2 (C-5), 37.3 (C-6), and 36.4 (C-2). The spectroscopic data [49,50] were consistent with literature reports for (1S,3R,4R,5R)-3-[(E)-3-(3,4-dihydroxyphenyl)prop-2-enoyl]oxy-1,4,5-trihydroxycyclohexane-1-carboxylic acid, also known as 3-caffeoylquinic acid or chlorogenic acid (**14**) (C_16_H_18_O_9_, MW 354.3 g/mol). The positive ion ESI-MS (Supplementary Data S40) showed *m*/*z* 355 [M+H]^+^ for the molecular formula C_16_H_19_O_9_.

### 2.2. Molecular Docking Against Monoamine Oxidases

#### 2.2.1. Docking Screening of the Isolated Compounds

Compounds (**1**–**9**) and (**11**–**14**), obtained from the methanolic extract of *T. americana* leaves, were evaluated for their potential interactions with MAO-A and MAO-B by molecular docking analysis. Compound (**7**) was used instead of the hexaacetylated derivative (**7a**), which was prepared exclusively for structural elucidation, because compound (**7**) is the natural metabolite present in the plant. Compound (**10**) was not included in the docking study because it corresponds to sucrose. Harmine and safinamide, the co-crystallized ligands of MAO-A and MAO-B, respectively, were used as reference compounds. The docking scores obtained for the protein–ligand complexes are summarized in Table 2 together with those of the reference ligands.

Most of the evaluated compounds produced less favorable docking scores in MAO-A than the co-crystallized ligand harmine (−8.2358 kcal/mol). In contrast, several compounds yielded favorable docking scores in MAO-B. Among them, β-sitosterol glucoside (**6**), 4α-terpineol sambubioside (**7**), tiliroside (**8**), and rutin (**9**) showed docking scores of −10.8733, −11.1434, −12.6986, and −12.9474 kcal/mol, respectively, compared with the docking score of the co-crystallized ligand safinamide (−7.2729 kcal/mol). The three-dimensional protein–ligand complexes obtained from the docking simulations for all tested compounds against both MAO-A and MAO-B are provided in the Supplementary Data (Table S1).

Because the objective of this study was to identify candidates for subsequent biological evaluation in a Parkinson’s disease model, compound selection was based on two complementary criteria: (i) favorable docking scores in MAO-B and (ii) the extent to which the predicted binding modes reproduced the binding orientation and key protein–ligand interactions established by the co-crystallized ligand safinamide within the MAO-B active site. Based on these criteria, compounds (**6**) and (**8**) were selected for the initial in vivo evaluation. Compound (**7**) also yielded a favorable docking score. Although only its hexaacetylated derivative (**7a**) was available for isolation and structural elucidation, molecular docking was performed using the natural compound (**7**), as it represents the metabolite present in the extract and fractions. Therefore, compound (**7**) was not included in the in vivo evaluation. Although rutin (**9**) exhibited one of the most favorable docking scores, it was not included in this initial biological evaluation and remains a promising candidate for future studies. Protein–ligand interaction analysis was subsequently performed to compare the interaction profiles of compounds (**6**) and (**8**) with those established by the reference ligand safinamide.

#### 2.2.2. Binding Mode Analysis of Selected Compounds (**6**) and (**8**)

**Figure 5:**
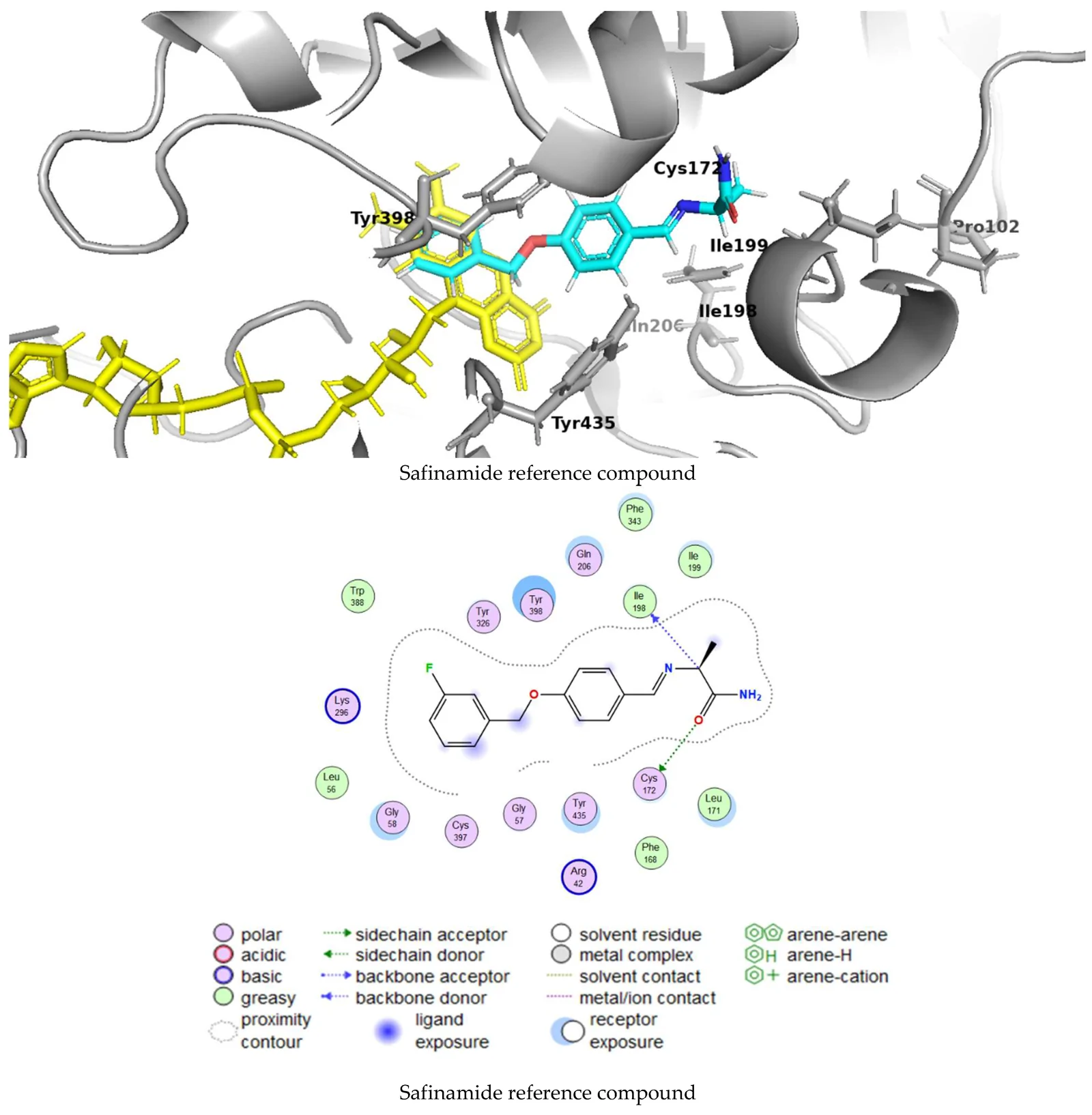

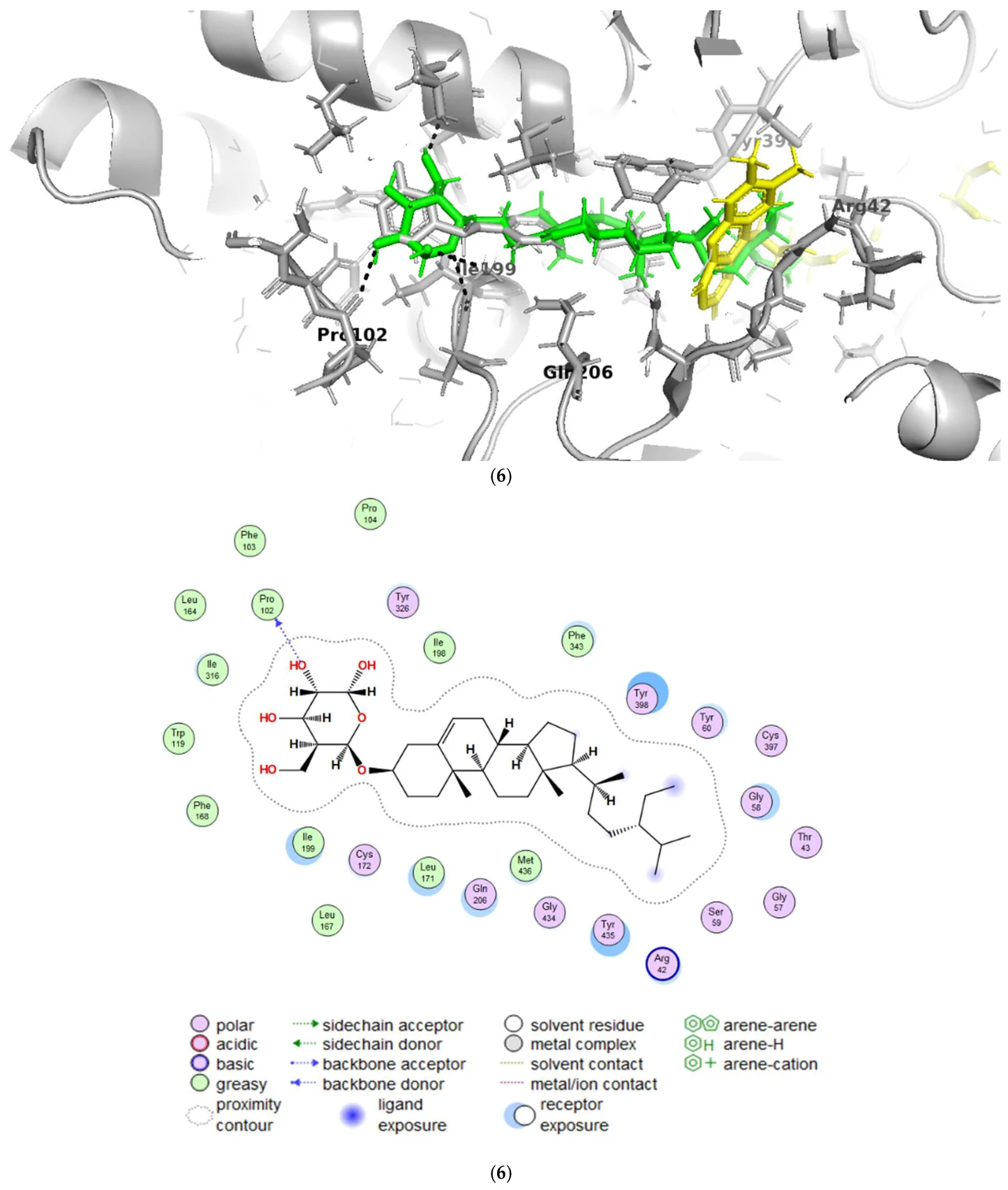

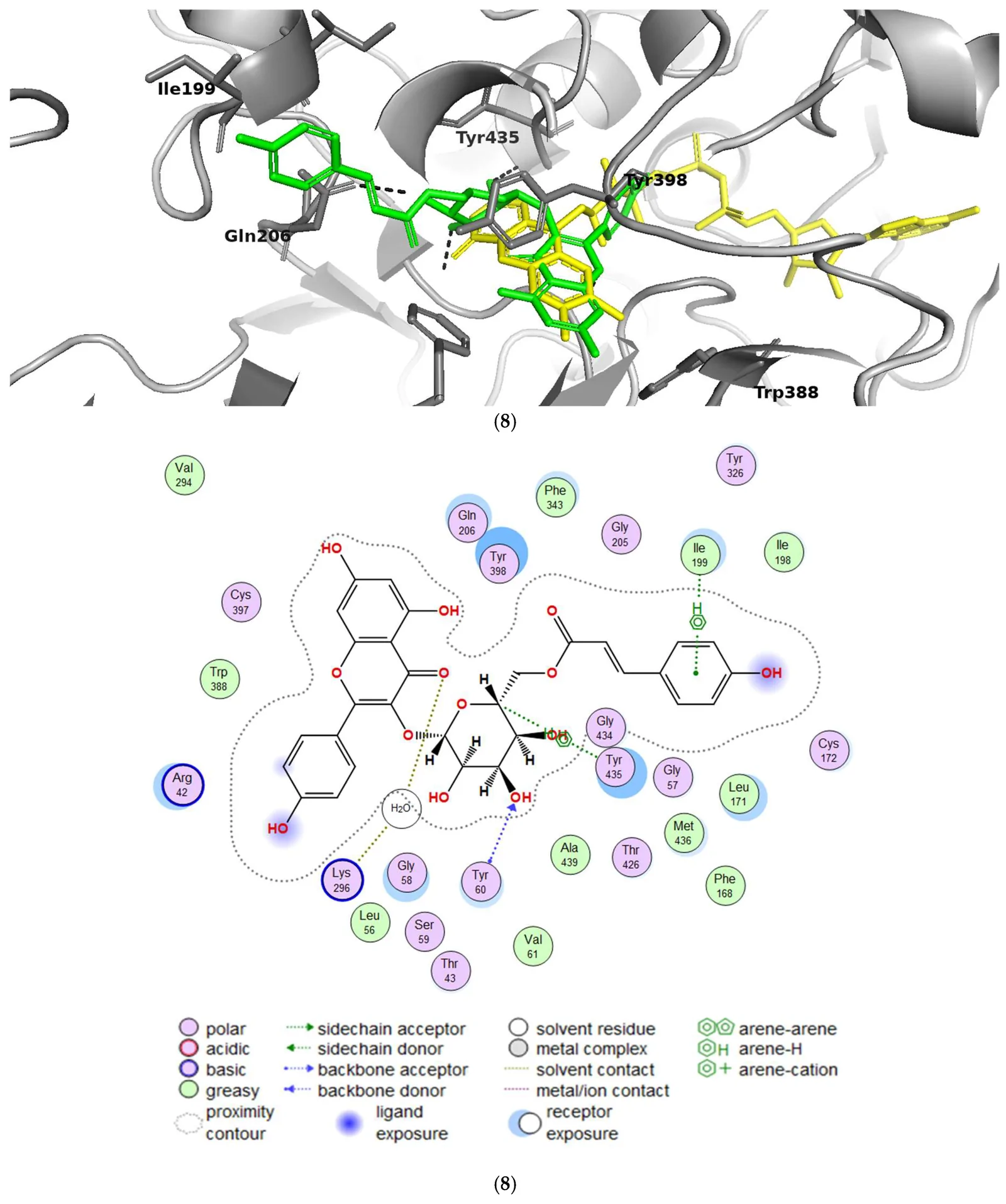
Three-dimensional (3D) representations of the binding poses of safinamide (reference compound) and compounds (**6**) and (**8**) in the MAO-B active site, together with two-dimensional (2D) protein–ligand interaction diagrams highlighting the key binding interactions. In the 3D views, the protein is displayed as a curved gray shape, key binding site residues as gray sticks, ligands as green sticks, and the FAD cofactor as yellow sticks. Hydrogen bonds are indicated by black dashed lines.

Figure 5 illustrates the predicted binding modes of the reference ligand safinamide together with β-sitosterol glucoside (**6**) and tiliroside (**8**) in the MAO-B binding site. The three-dimensional binding poses and two-dimensional protein–ligand interaction diagrams for all compounds docked against MAO-B are provided in the Supplementary Data (Table S2).

The interactions between the selected compounds (**6**) and (**8**) and amino acid residues within the MAO-B active site are summarized in Table 3.

Additionally, the MAO-B amino acid interaction diagrams for all tested compounds are included in the Supplementary Data (Table S3).

Docking analysis showed that the co-crystallized ligand safinamide binds within the MAO-B active site through an extensive network of polar, hydrophobic, and other non-covalent interactions (Table 3). This interaction pattern served as the structural reference for comparison with the selected compounds.

β-Sitosterol glucoside (**6**) reproduced several of the interactions established by safinamide, particularly with residues Cys172, Ser59, Gly57, Tyr60, Cys397, Phe168, Leu164, Ile316, Gln206, Tyr398, Tyr435, Ile199, and Leu171 (Table 3).

Likewise, tiliroside (**8**) shared several interactions with the reference ligand, including contacts with Cys397, Gly205, Cys172, Gly57, Ser59, Phe168, Ile198, Tyr398, Tyr60, Gly58, Ile199, and Leu171, together with additional backbone donor, solvent contact, and arene–H interactions (Table 3).

### 2.3. Behavioral Evaluation of Reserpine-Induced Parkinsonism in Mice

Following molecular docking, β-sitosterol glucoside (**6**) and tiliroside (**8**) were selected for biological evaluation in a reserpine-induced mouse model of parkinsonism based on their favorable predicted binding modes within the MAO-B active site. Accordingly, the effects of the methanolic extract (Ta-MeOH), the fractions (Ta-R1, Ta-R3, and Ta-R4), and compounds (**6**) and (**8**) were evaluated using the Irwin observational test, the open field test (OFT) to assess spontaneous locomotor activity, and the Rota-Rod test to evaluate motor coordination, allowing for the experimental findings to be compared with the molecular docking results. The order for performing the tests was the Irwin test, then the open field test, and finally the Rota-Rod test. Mice were allowed a rest period between each test.

#### 2.3.1. Irwin Test

The behavioral variables in the Irwin test were evaluated at different times during the experiment. This test was performed qualitatively and in an exploratory manner. The behavior induced with RES can be observed, along with the effects of different treatments. RES induced severe neurological signs and impairment, including abdominal writhing, piloerection, palpebral ptosis, motor alterations, huddling, constipation, and reduced intestinal transit, which were absent in the basal group. In contrast, the positive control group (L-DOPA) exhibited progressive improvement starting on day 15, with near-complete resolution of signs by day 23, although mild stereotyped behaviors were observed in the final evaluation (see Table 4).

In the group treated with Ta-MeOH (100 mg/kg, p.o.), as shown in Table 5, the severity of reserpine-induced signs gradually decreased. Abdominal writhing, piloerection, and palpebral ptosis evolved from severe to mild or absent by day 23. Motor alterations, huddling, and loss of muscle tone, which remained between severe and moderate in the RES group, were reduced to mild levels with Ta-MeOH treatment. Constipation decreased from severe to absent, and the pattern of reduced intestinal transit was comparable to that of the control group, resolving by the end of treatment. Fractions Ta-R1, Ta-R3, and Ta-R4 displayed similar trends, supporting their inclusion, as they contain previously isolated compounds. Compounds (**6**) and (**8**) were also confirmed; the results are shown in Table 6. The group of animals with (**8**) showed effects in almost all parameters, except for altered movements and loss of muscle tone. At the same time, mice with (**6**) presented a recovery of almost all symptoms except abdominal contortions and rising hair.

On day 23, the group that received only RES still showed signs of neurotoxicity, including abdominal contortion, hair standing on end, palpebral ptosis, altered movements, huddling, and loss of muscle tone. The group treated with L-DOPA exhibited stereotyped behaviors.

#### 2.3.2. Open Field Test (OFT)

The behavioral effects of the methanolic extract (Ta-MeOH), fractions (Ta-R1, Ta-R3, Ta-R4), and compounds (**6**, **8**) were evaluated in the open field test (OFT). Locomotor behavior was assessed by measuring total crossings on days 6, 8, 15, and 23. The longitudinal response was summarized as the area under the curve (AUC) using the trapezoidal method.

**Figure 6:**
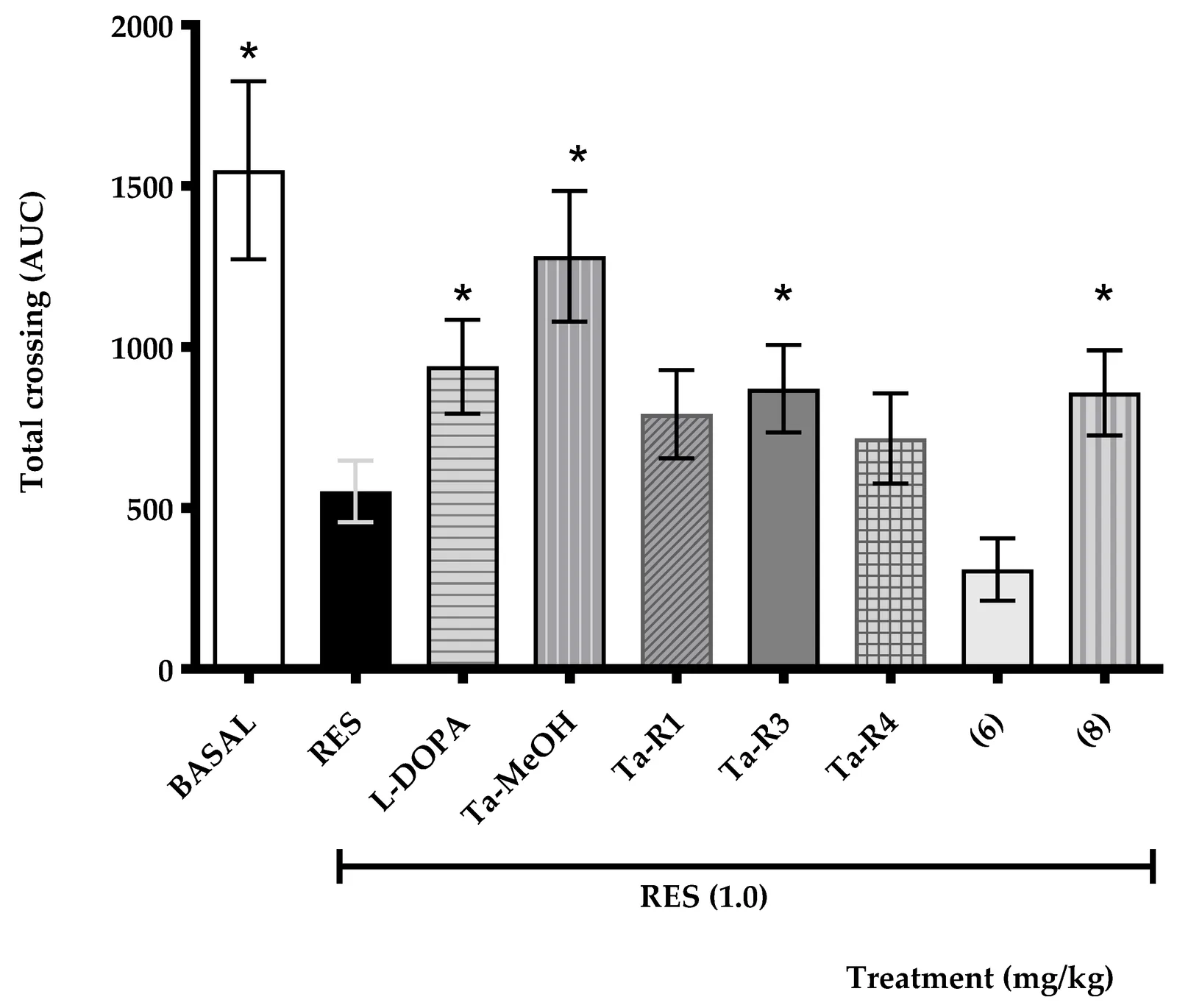
Effect of the methanolic extract of *T. americana* (Ta-MeOH), fractions (Ta-R1, Ta-R3, Ta-R4), and compounds (**6**, **8**) on mice with reserpine-induced Parkinson’s disease subjected to the open field test (OFT). Data are expressed as the area under the curve (AUC) of total crossings. RES = group of mice treated with reserpine (RES), without treatment; BASAL = healthy animals. All data were analyzed by one-way ANOVA followed by a post hoc Dunnett’s test. *p* < 0.05 (N = 6) compared with the RES group (damage without treatment). All analyses were performed using the SPSS 11.0 statistical package. * *p* < 0.05, statistically significant compared with the RES group.

In this test, healthy mice showed an AUC of 1558, whereas the RES group showed a significant decrease to 551.7. L-DOPA showed a value of 934.41, while Ta-MeOH, tiliroside (**8**), and Ta-R3 showed values of 1281.83, 858, and 870.1, respectively. The RES group exhibited a lower AUC than the BASAL group, whereas Ta-MeOH significantly increased locomotor activity compared with the RES group. According to the statistical analysis (Figure 6), the Ta-R3, L-DOPA, Ta-MeOH, and tiliroside (**8**) groups showed a significant difference compared with the RES group (*p* < 0.05). Ta-R1 also showed an increase in AUC; however, this difference was not statistically significant compared with the RES group (*p* > 0.05).

#### 2.3.3. Motor Coordination Test in Rota-Rod Apparatus

Fine motor coordination was assessed using the Rota-Rod test by measuring latency to fall at 4, 10, and 20 rpm on days 6, 8, 15, and 23. The longitudinal response was summarized as the area under the curve (AUC) using the trapezoidal method.

**Figure 7:**
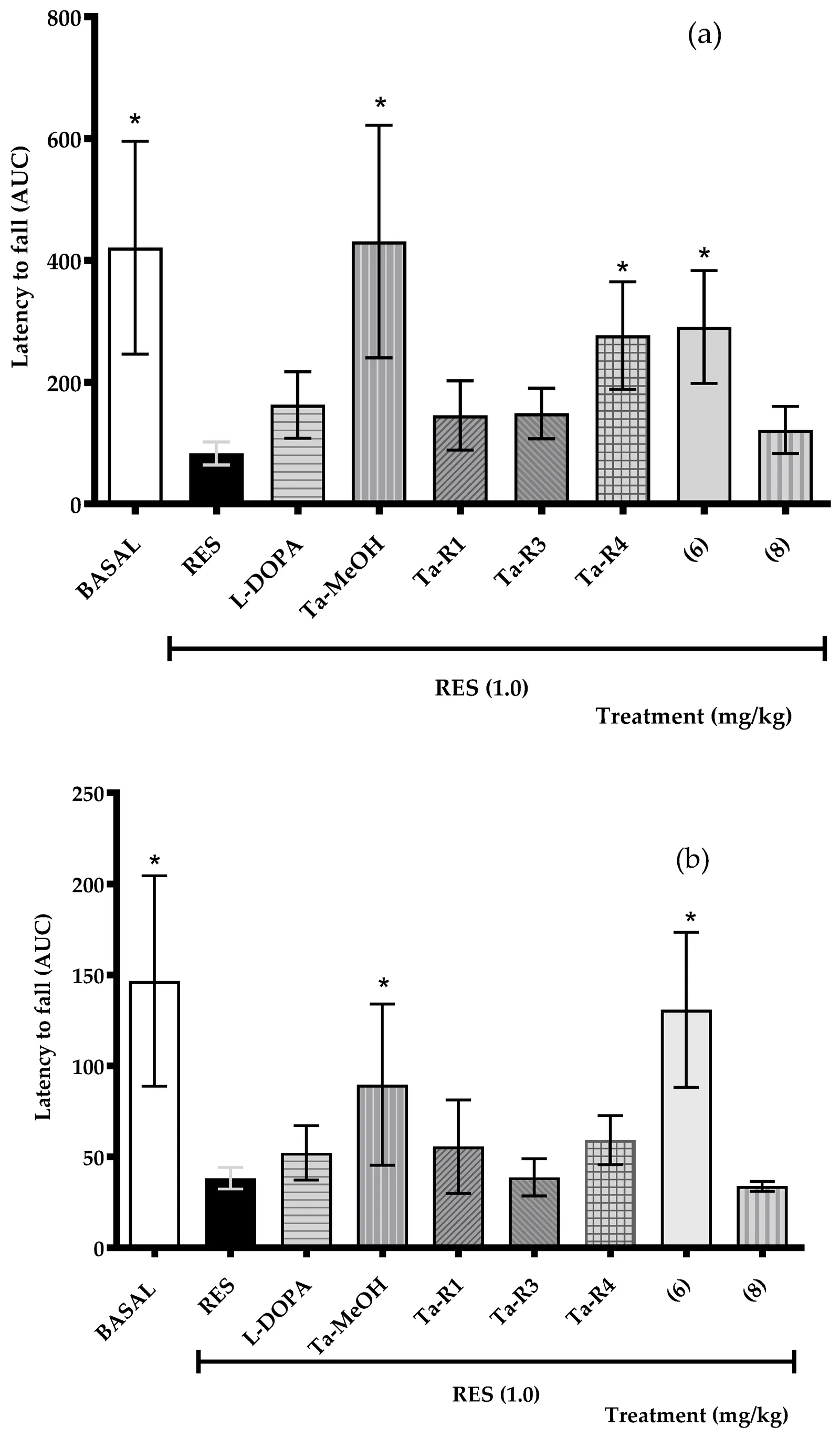

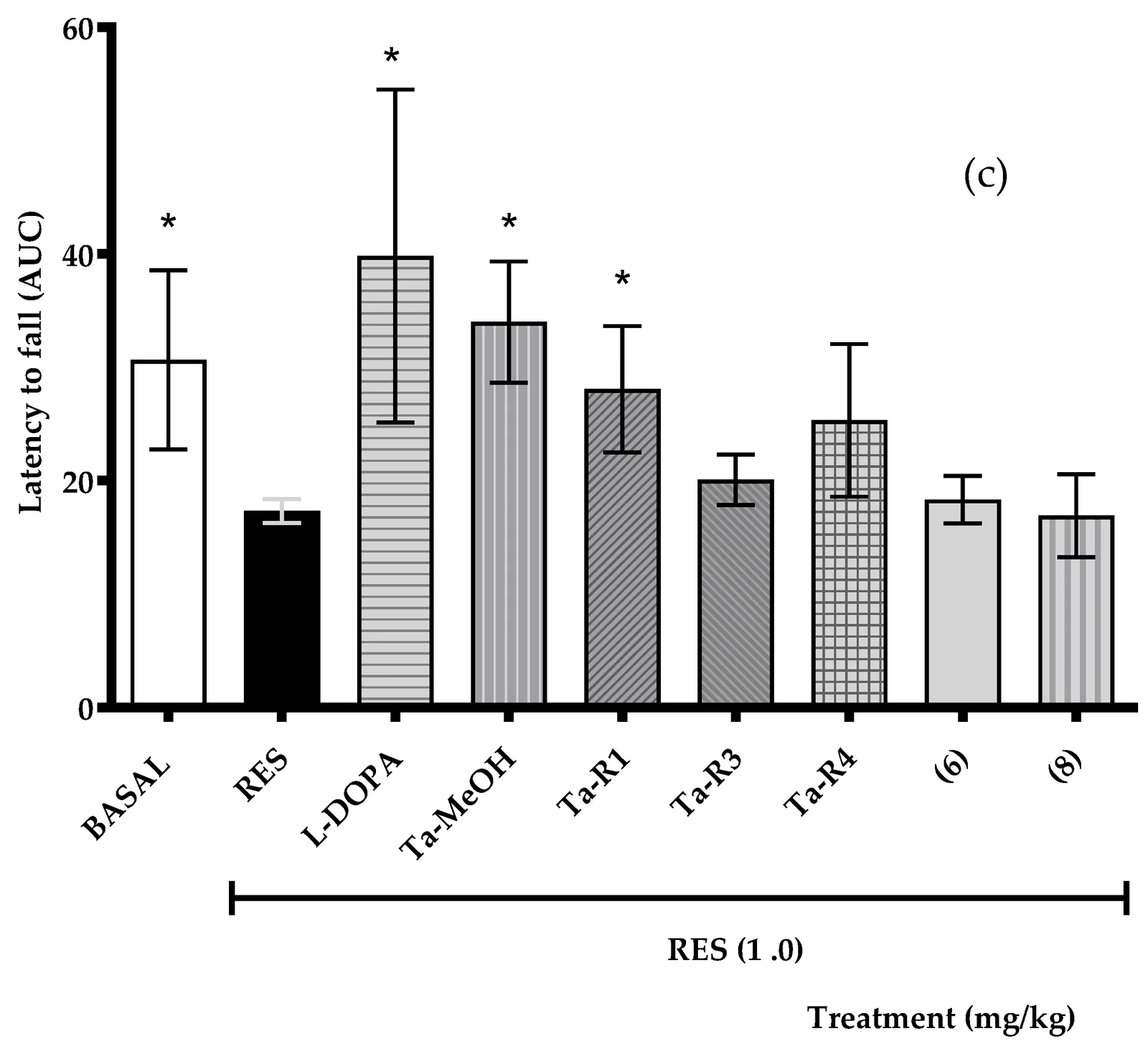
Effect of the methanolic extract of *T. americana* (Ta-MeOH), fractions (Ta-R1, Ta-R3, and Ta-R4), and compounds (**6** and **8**) on mice with reserpine-induced Parkinson’s disease (PD) subjected to the Rota-Rod test. (**a**) 4 rpm; (**b**) 10 rpm; (**c**) 20 rpm. Data are expressed as mean AUC ± SD of latency to fall. RES = reserpine-treated group without treatment; BASAL = healthy animals. N = 6. All data were analyzed by one-way ANOVA followed by Dunnett’s post hoc test. *p* < 0.05 compared with the RES group. Statistical analyses were performed using SPSS version 11.0. * *p* < 0.05, statistically significant compared with the RES group.

The results at 4, 10, and 20 rpm are presented in Figure 7a–c. RES administration resulted in lower AUC values for latency to fall at 4, 10, and 20 rpm, with values of 82, 38, and 17, respectively, compared with healthy animals, which showed values of 420, 146, and 30.6, respectively. L-DOPA showed a significant improvement at 4 and 20 rpm, with AUC values of 162.7 and 39, respectively. Among the treatments derived from the plant, Ta-MeOH showed the highest AUC values, with 431, 89, and 34 at 4, 10, and 20 rpm, respectively.

At 4 rpm, the Ta-MeOH group showed a response comparable to that of the BASAL group, see Figure 7a, with a significant difference compared with the RES group (*p* < 0.05). Similarly, Ta-R4 and compound (**6**) showed significant differences compared with the RES group. At 10 rpm, a statistically significant improvement in fine motor coordination was observed in the Ta-MeOH and compound (**6**) groups compared with the RES group, *p* < 0.05, see Figure 7b. At 20 rpm, significant differences compared with the RES group were observed for L-DOPA, Ta-MeOH, and Ta-R1, *p* < 0.05, see Figure 7c.

## 3. Discussion

In this study, fourteen compounds were isolated and identified from *T. americana*, including seven terpenoid compounds, five of which were triterpenes. Compound (**6**) was identified as a β-sitosterol glucoside, whereas compound (**7**), a diglycosylated monoterpene, is reported here for the first time in this species. In addition, two flavonoids (compounds **8** and **9**) were isolated, together with one sugar (compound **10**) and four phenolic compounds (compounds **11**–**14**).

Flavonoids have been widely associated with the biological activities reported for *T. americana* and other *Tilia* species [20]. Tiliroside, rutin, quercitrin, quercetin glucoside, and kaempferol have been reported to exhibit hypoglycemic, antioxidant, anxiolytic, antidepressant, anti-inflammatory, anticancer, and neuropharmacological properties [23,25,27,28]. These compounds have been isolated from extracts of different polarities, including methanolic, aqueous, and ethyl acetate extracts [51,52,53,54,55].

In contrast, terpenoid constituents of *T. americana* have received comparatively little attention in previous pharmacological studies. The present findings suggest that these metabolites may also contribute to the behavioral improvements observed in the reserpine-induced Parkinson’s disease model. Molecular docking showed that compounds (**6**) and (**7**) displayed favorable interactions with the catalytic site of MAO-B, a validated therapeutic target in Parkinson’s disease and other neurological disorders. Although molecular docking does not demonstrate enzyme inhibition or therapeutic efficacy, these findings suggest that modulation of MAO-B could represent one of the mechanisms contributing to the behavioral effects observed in vivo.

Previous studies have demonstrated antidepressant-like effects of both the methanolic extract of *T. americana* and the isolated flavonoid tiliroside (compound **8**) in mice subjected to the forced swimming and tail suspension tests. However, the molecular mechanisms underlying these effects remain unclear. In the present study, MAO-A and MAO-B were evaluated as potential molecular targets for the isolated compounds to explore possible mechanisms associated with the behavioral effects observed in the reserpine-induced Parkinson’s disease model. Nevertheless, further enzymatic and mechanistic studies are required to confirm whether modulation of these targets contributes to the pharmacological activity of *T. americana*.

After analyzing the predicted molecular interactions, differences in docking scores were observed among the compounds of *T. americana* for MAO-A and MAO-B. These differences can be explained by the structural characteristics of the two isoforms. Although MAO-A and MAO-B share a high degree of identity (73.7%) and sequence similarity (89.5%), they differ in the conformation and dimensions of their catalytic cavities, which influence ligand recognition. Crystallographic studies have shown that these differences are mainly associated with four amino acid substitutions (Ile180/Leu171, Asn181/Cys172, Phe208/Ile199, and Ile335/Tyr326). In particular, the Phe208 residue of MAO-A creates steric hindrance that restricts the accommodation of elongated ligands, whereas the corresponding Ile199 residue in MAO-B favors their binding within the active site [56]. Consequently, the differences observed in the docking results among the isolated compounds may be explained by both the geometry of the catalytic pocket and the number, type, and spatial arrangement of their predicted interactions with specific amino acid residues [57].

These findings are consistent with previous studies highlighting the importance of hydrophobic and aromatic interactions in ligand recognition by MAO-B. Several reported MAO-B inhibitors establish arene–H interactions with residues such as Ile199, Leu171, and Phe343, as well as contacts with Cys127 and Phe168 [58]. Likewise, phytosterols such as β-sitosterol and stigmasterol have been predicted to interact mainly through hydrophobic contacts with Leu88, Pro102, Pro104, Trp119, Phe168, and Ile199, together with hydrogen bonds involving residues such as Leu164 [59]. Other reported MAO-B ligands also display combinations of hydrogen bonds with Cys172 and π–π or π–alkyl interactions involving Tyr398, Phe343, Tyr326, Ile199, and Leu171 [58]. Collectively, these studies indicate that the molecular recognition of ligands by MAO-B depends not only on the number of interactions established but also on their nature and location within the catalytic pocket, where residues such as Ile199, Phe168, and Tyr398 appear to play an important role in stabilizing protein–ligand complexes [59].

The interaction profiles predicted for β-sitosterol glucoside (**6**) and tiliroside (**8**) revealed contacts with several key residues also involved in safinamide binding, including Ile199, Leu171, Phe168, Pro102, Pro104, Trp119, and Leu164. These shared interactions support the predicted binding modes of both compounds within the MAO-B active site.

Because MAO-B plays a central role in dopamine metabolism, it remains an important therapeutic target for Parkinson’s disease. Based on the molecular docking results, β-sitosterol glucoside (**6**) and tiliroside (**8**) were prioritized for biological evaluation in a reserpine-induced mouse model of parkinsonism. Compound (**7**) was not included in the in vivo evaluation because only its acetylated derivative (**7a**), prepared for structural elucidation, was available. Although rutin (**9**) also exhibited one of the most favorable docking scores, it was not included in this initial biological evaluation and remains an interesting candidate for future pharmacological studies. Likewise, although tiliroside (**8**) has previously been reported to exhibit antidepressant activity [28], whether this activity is associated with modulation of MAO remains to be established through dedicated biochemical studies.

In this study, a first approach to the biological effects of the methanolic extract (Ta-MeOH); the Ta-R1, Ta-R3, and Ta-R4 fractions; as well as β-sitosterol glucoside (**6**) and tiliroside (**8**) was carried out using a reserpine-induced mouse model of parkinsonism. Behavioral alterations were evaluated using the Irwin Observation Test, spontaneous locomotor activity was assessed by the OFT, and motor coordination was evaluated using the Rota-Rod test. Overall, the different preparations obtained from *T. americana* produced distinct behavioral responses, suggesting that different metabolites may contribute to different aspects of the biological activity of the extract.

Reserpine induces depletion of monoamines, including dopamine, norepinephrine, and serotonin, by interfering with their storage in synaptic vesicles through ATP- and magnesium-dependent mechanisms. Consequently, animals develop behavioral alterations characteristic of this experimental model, including hypolocomotion, ptosis, muscular rigidity, and other signs associated with monoaminergic depletion, in agreement with previous reports [60,61]. Administration of reserpine also markedly reduced spontaneous locomotor activity, a parameter commonly used to evaluate motor impairment in experimental models of Parkinson’s disease.

Treatments derived from *T. americana* attenuated several of these behavioral alterations, although each preparation displayed a distinct activity profile. The OFT is widely used to evaluate exploratory behavior and spontaneous locomotor activity in experimental models of neurological disorders [62,63,64,65,66,67]. Under the experimental conditions employed, Ta-MeOH, Ta-R3, and compound (**8**) significantly increased the number of crossings compared with the reserpine-treated group, indicating an improvement in locomotor performance.

The behavioral profile of compound (**8**) is particularly interesting because this flavonoid has previously been reported to exhibit antidepressant- and sedative-like effects in healthy mice, where it reduced spontaneous locomotor activity during exploration [28]. In contrast, in the present study, compound (**8**) increased locomotor activity in animals with reserpine-induced motor impairment. These apparently different responses suggest that its pharmacological profile may vary depending on the physiological or pathological condition under which it is evaluated. In addition, previous studies have shown that tiliroside potentiates the antiparkinsonian effects of lisuride and exhibits antioxidant and anti-inflammatory activities [68,69,70]. Although the present study was not designed to establish the mechanisms underlying the behavioral improvements observed, these previously reported properties provide a plausible biological context for the effects detected in the reserpine model.

Motor coordination impairment is one of the principal manifestations of Parkinson’s disease and can be evaluated experimentally using the Rota-Rod test. In the present study, Ta-MeOH, Ta-R4, and β-sitosterol glucoside (**6**) significantly improved animal performance compared with the reserpine-treated group, indicating better motor coordination under the experimental conditions employed. Among these treatments, compound (**6**) produced the most consistent effect. Because the Rota-Rod evaluates balance, coordination, and fine motor control, the increased latency to fall reflects an improvement in motor performance; however, the molecular mechanisms responsible for this effect cannot be established from the present study alone [65,66].

β-Sitosterol has been extensively investigated because of its antioxidant and anti-inflammatory properties [71,72]. More recently, phytosterols have also been reported to exert neuroprotective effects in experimental models through modulation of oxidative stress, neuroinflammatory responses, and neuronal signaling pathways [73]. For example, β-sitosterol improved motor performance and reduced oxidative damage in a model of vanadium-induced neurotoxicity [74]. These previous findings are consistent with the behavioral improvements observed in the present study and support the continued investigation of β-sitosterol glucoside as one of the bioactive constituents of *T. americana*. Nevertheless, additional biochemical studies will be required to determine the molecular pathways involved in its biological effects.

Interestingly, β-sitosterol glucoside (**6**) improved motor coordination in the Rota-Rod test without significantly modifying spontaneous locomotor activity in the OFT, whereas tiliroside (**8**) exerted a more evident effect on spontaneous locomotor activity. These differences suggest that the isolated compounds may influence distinct components of the behavioral alterations induced by reserpine rather than producing a generalized stimulatory effect.

Overall, the isolated compounds displayed complementary activity profiles. β-Sitosterol glucoside (**6**) was associated primarily with improvements in motor coordination, whereas tiliroside (**8**) showed greater effects on spontaneous locomotor activity and behavioral parameters recorded during the Irwin and OFT evaluations. Although these responses were less pronounced than those produced by L-DOPA, both compounds attenuated behavioral alterations induced by reserpine. These findings are consistent with their selection following the molecular docking analysis and support their further investigation as bioactive constituents of *T. americana*. However, additional biochemical and pharmacological studies will be necessary to identify their molecular targets and clarify the mechanisms responsible for their biological activity.

While this study represents an initial approach to assessing the inhibitory potential of the methanolic extract and isolated compounds from *Tilia americana* var. *mexicana* against MAO-A and MAO-B through molecular docking, these findings require further experimental validation. Although docking suggested potential interactions with MAO-B, the behavioral improvements observed in the reserpine-induced Parkinsonian model cannot be considered direct evidence of MAO-B inhibition, as MAO enzymatic activity, brain monoamine concentrations, and target engagement were not assessed. In vitro assays to determine inhibitory activity, IC_50_ values, MAO-B selectivity, and the mechanism of inhibition, together with molecular dynamics simulations, would provide complementary evidence to validate the computational predictions and better characterize ligand–protein interactions.

Some methodological considerations should also be acknowledged. Behavioral assessments and data analyses were not performed under blinded conditions, as all experimental procedures were conducted by the same investigator to ensure methodological consistency. The absence of blinding, particularly during the qualitative Irwin test, may introduce observer bias. In addition, although a formal *a priori* sample size or power calculation was not performed, the sample size was established based on previous studies using similar experimental models and in accordance with the principles of the 3Rs. Future studies incorporating blinded assessments, formal power calculations, and complementary biochemical and computational approaches may further strengthen the robustness of the findings and provide additional insight into the mechanisms underlying the pharmacological effects of *T. americana* var. *mexicana* metabolites**.**

Taken together, the integration of these experimental and computational approaches will contribute to a deeper understanding of the mechanisms of action of *T. americana* var. *mexicana* metabolites and help determine their potential as candidates for the development of new therapeutic strategies for Parkinson’s disease.

## 4. Materials and Methods

### 4.1. Equipment and Reagents

NMR spectra were obtained on an Agilent DD2-600 (Agilent Technologies, Santa Clara, CA, USA) equipped with an ultra-cool probe at 600 MHz for ^1^H and 150 MHz for ^13^C NMR, using CDCl_3_, CD_3_OD, or DMSO-d_6_ as the solvent. Bidimensional experiments (COSY, HSQC, and HMBC) were carried out for samples (**7a**) and (**8**). Chemical shifts are reported in ppm relative to TMS. Mass spectrometric analyses were performed using a Waters triple quadrupole mass spectrometer (Waters Corporation, Milford, MA, USA) equipped with a ZSpray electrospray ionization (ESI) source maintained at 150 °C. Argon was used as the collision gas at a flow rate of 0.10 mL min^−1^. For high-resolution HR-ESI-MS mass determination of (**7a**), a microOTOF II mass spectrometer (Bruker Daltonics Inc., Billerica, MA, USA) was used with the Compass platform (OTOF Control and DataAnalysis by Bruker Daltonics Inc.). The spectra were acquired in positive mode with a capillary voltage of 4500 V, nitrogen as the nebulization gas at 0.5 bar, a drying gas flow rate of 4.0 L/min, and a drying temperature of 150 °C. UV chromatograms were obtained using an HPLC system (Waters 2695 Separation Module, Waters, Milford, MA, USA) equipped with a diode array detector (UV detection range 200–600 nm). A Discovery^®^ C18 (5 μm) column (L × I.D. 25 cm × 4.6 mm; Supelco, Merck KGaA, Darmstadt, Germany) and the Pro-3 software package (Version 3.8.1, Waters, Milford, MA, USA) using the methodology described in the literature [75] were used. Melting points were determined using a Barnstead International/Thermolyne MEL-TEMP capillary melting point apparatus (Model 1001). Barnstead Thermolyne Corp., Dubuque, IA, USA). Optical rotations were measured using a PerkinElmer 341 polarimeter (PerkinElmer Inc., Waltham, MA, USA).

Reagent-grade methanol (GR), dichloromethane, methanol-HPLC, acetonitrile-HPLC, and acidulated water-HPLC (0.05 TFA) were used as solvents for chemical separation; these reagents were purchased from Sigma-Aldrich, Naucalpan de Juárez, Mexico. Normal-phase TLC silica gel 60 F254 plates and TLC silica gel 60 RP_18_ F254S plates were used for TLC. The chromatoplates were analyzed using an ultraviolet lamp with a wavelength of 254–365 nm. For the physical detection of terpenes and flavonoids, 4-hydroxybenzaldehyde and 2-amino-ethyl-diphenyl-borinate were used, respectively, as was serum ammonia sulfate.

### 4.2. Plant Material

Leaves were collected from wild *T. americana* var. *mexicana* in Mexicapa, State of Mexico, in the following locations: 18°59′20.22″ N, 99°19′11.04″ W, at an altitude of 2205 m, and 18°59′28.05″ N, 99°19′26.16″ W, at 2327 m above sea level. A specimen was prepared for identification by Ms. Abigail Aguilar Contreras at the IMSS Herbarium of the Siglo XXI National Medical Center and was granted registration number 15099.

#### Extracts Obtention

The leaves of *T. americana* var. *mexicana* were dried at room temperature and ground to a particle size of approximately 4 mm using a Pulvex^®^ mechanical mill (Pulvex, Mexico). A 3000 g quantity of dried, ground leaves were placed in a 20 L container and degreased by maceration in 9 L of hexane for 72 h. The plant material was filtered, and the liquid was concentrated by distillation under reduced pressure using a Heidolph^®^ rotary evaporator (Hei-VAP Advantage) (Heidolph Instruments GmbH & Co. KG, Schwabach, Germany).

The extract obtained was dried at room temperature to produce the hexane extract (Ta-Hex). Next, 9 L of methanol were added to the plant material, which was then left to rest for 24 h. The resulting solution was filtered, and the solvent was then concentrated using a rotary evaporator. This process was repeated 4 times to obtain the methanolic extract (Ta-MeOH). Following extraction with n-hexane, 22.1 g of Ta-Hex was obtained, while extraction with methanol yielded 249.9 g of Ta-MeOH.

### 4.3. Chromatographic Fractionation of Ta-MeOH

A 170.3 g quantity of the methanolic extract was adsorbed onto 130 g of silica gel (0.063–0.2 mm, Merck, Papenburg, Germany) and placed in a 10 × 30 cm open glass column packed with silica gel. The column was eluted with a CH_2_Cl_2_ gradient, increasing the polarity by 10% with CH_3_OH as the mobile phase. Fractions of 300 mL were collected, yielding 59 fractions. The chemical separation was monitored by thin-layer chromatography (TLC), and the compounds were grouped by retention factor (Rf) and chemical similarity into 5 fractions (Ta-R1 to Ta-R5). These fractions were as follows: Ta-R1 (fractions 1–9, 100% CH_2_Cl_2_), Ta-R2 (fractions 10–24, 95:5 CH_2_Cl_2_: CH_3_OH), Ta-R3 (fractions 25–26, 90:10 CH_2_Cl_2_: CH_3_OH), Ta-R4 (fractions 27–40, 80:20 and 70:30 CH_2_Cl_2_: CH_3_OH), and Ta-R5 (fractions 41–59, 50:50 CH_2_Cl_2_:CH_3_OH, 100% CH_3_OH). The chromatographic separation used to obtain compounds (**1**–**10**) is described below.

#### 4.3.1. Isolation of Compounds (**1**–**10**)

Compounds (**1**–**5**) were obtained from fraction Ta-R1. Their structure was elucidated based on ^1^H and ^13^C NMR. The spectroscopic data closely matched reported values, confirming the identification of this compound as a known triterpenoid sterol.

The Ta-R1 fraction (8.5 g), which contains terpenes, was absorbed onto silica gel (10 g, 70–230 mesh, Merck) and placed into a column (3 × 40 cm) packed with activated charcoal (Meyer). An isocratic system of CH_2_Cl_2_:CH_3_OH (50:50) was used as the mobile phase, with a total volume of 400 mL. The mixture was concentrated on a rotary evaporator to give a waxy white precipitate (R1Fp). This was washed with methanol to give 7.94 g of R1Fp, which showed a single spot in TLC revealed with 4-hydroxybenzaldehyde. ^1^H and ^13^C NMR identified this as a mixture of α/ β-amyrine (**1**) and (**2**).

A 1 g quantity of the mother liquor (R1FAm), soluble in methanol, was absorbed with silica gel (1 g, 70–230 mesh, Merck) and placed in a glass column (2 × 30 cm) packed with silica gel (15 g). The column was eluted with a 5% gradient of increasing polarity with ethyl acetate (AcOEt), collecting 50 mL fractions. Fifty-two fractions were obtained. Fractions 17 to 24 (95:5 hexane: AcOEt) also contained the mixture of α/ β-amyrine (**1**) and (**2**). Fractions 29 to 31 (95:5 hexane: AcOEt) contained a mixture of β-sitosterol (**3**) and stigmasterol (**4**). Fractions 32 to 35 (90:10 hexane: AcOEt) contained a white solid corresponding to ursolic acid (**5**)**.**

A 5 g quantity of the Ta-R2 fraction was adsorbed onto silica gel (10 g, 70–230 mesh, Merck) and placed in a glass column (3 × 40 cm) packed with silica gel (80 g). Elution started with CH_2_Cl_2_, and the polarity gradient was increased by 5% with CH_3_OH to give 69 fractions of 50 mL each. Fractions 23–28 (95:5 CH_2_Cl_2_:CH_3_OH) produced a white solid, which was washed with CH_3_OH and identified as β-sitosterol glucoside (**6**).

A 14 g quantity of Ta-R4 was adsorbed onto silica gel (15 g, 70–230 mesh, Merck) and placed in a column (3 × 40 cm) packed with silica gel (45 g). Elution was initiated with CH_2_Cl_2_, and the polarity gradient was increased with 10% CH_3_OH to obtain 60 fractions, each containing 100 mL. Fractions 18–27 (6 g, 80:20 CH_2_Cl_2_:CH_3_OH) were combined and subjected to reverse-phase chromatographic separation using 3 g of silica (POLYGOPREP^®^ 60-50 C18, MACHEREY-NAGEL GmbH & Co. KG, Düren, Germany). Elution started with 100% H_2_O, and polarity decreased using acetonitrile (CH_3_CN) with a 10% polarity gradient; 10 mL fractions were collected and concentrated to yield 64 fractions. Fractions 13–15 (50 mg, 90:10 H_2_O:CH_3_CN) exhibited similar TLC profiles, prompting the performance of an acetylation reaction to yield 80 mg of the organic phase (ethyl acetate fraction). The crude reaction product (80 mg) was adsorbed onto 1 g of normal-phase silica gel and placed into a glass column (2 × 30) cm) packed with silica gel (10) g. The elution was started with hexane, with the polarity increased in 10% increments using ethyl acetate, and 25 mL fractions were collected, yielding 50 vials. Fractions 40–44 (80:20 hexane: AcOEt) precipitated a white solid when washed and filtered with methanol. This corresponds to 4α-terpineol sambubioside hexaacetate (**7a**, 90.9% purity, mp 134–138 °C; [α]D −40 (c 0.3, CH_2_Cl_2_)). On the other hand, a yellow solid corresponding to tiliroside (**8,** 99.15% purity) was obtained by precipitation with CH_3_OH in fractions 48 to 52.

**Figure 8:**
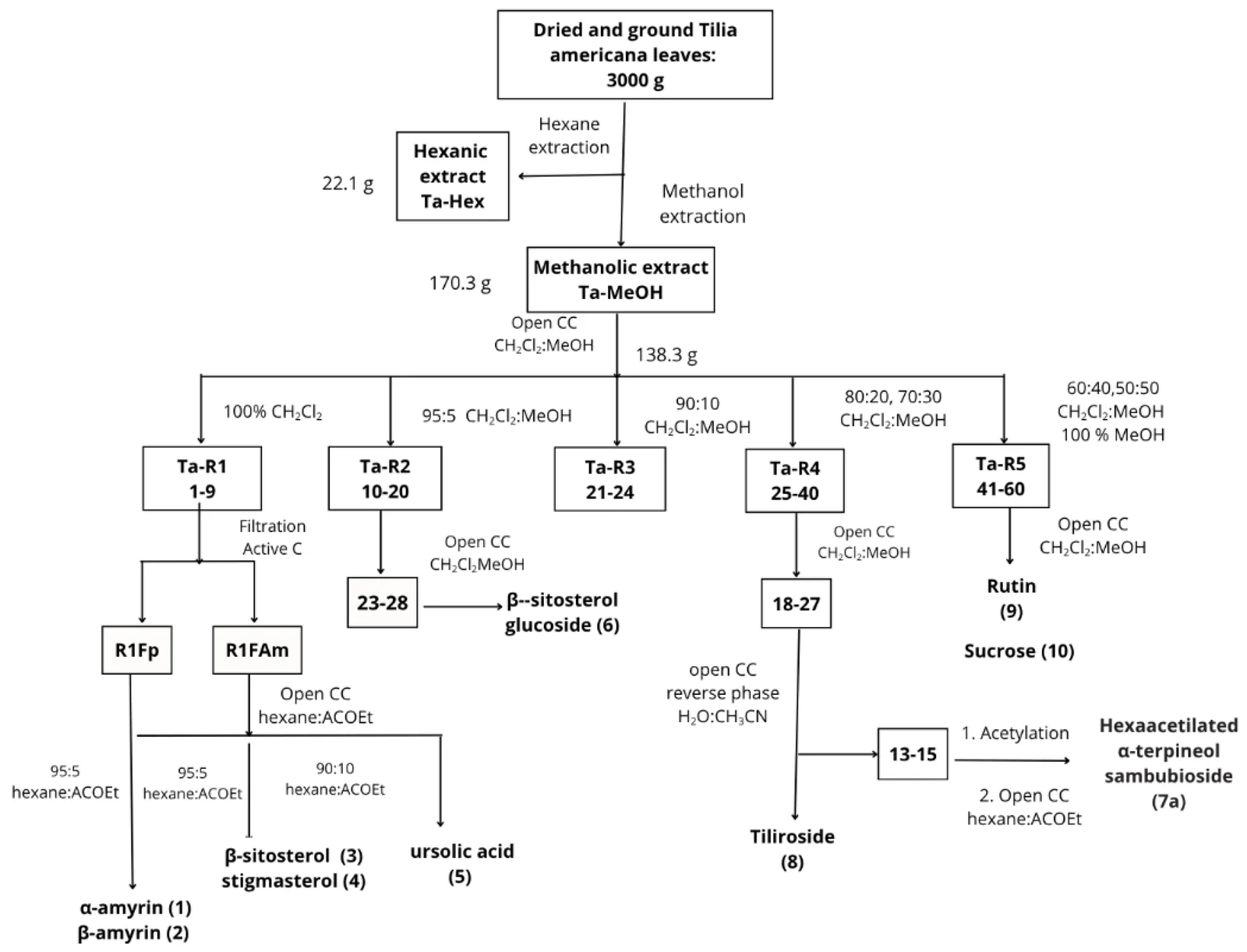
Strategy for the separation of compounds (**1**–**10**).

A 2.6 g quantity of the Ta-R5 mixture was adsorbed onto 4 g of silica gel and placed in a column (2 × 40 cm) packed with silica gel (12 g). Elution was initiated with dichloromethane, with the polarity gradually increased in 10% increments with methanol, yielding 50 fractions of 25 mL each. Fractions 18 to 25 (85:15 CH_2_Cl_2_:CH_3_OH) yielded a yellowish solid by precipitation, corresponding to rutin (**9**). In Ta-R5, fractions 56 to 60 (100% CH_3_OH) produced a slightly orange solid by precipitation and subsequent washing with methanol. This was identified as sucrose (**10**). Figure 8 summarizes this separation process.

#### 4.3.2. Isolation of Compounds (**11**–**14**)

**Figure 9:**
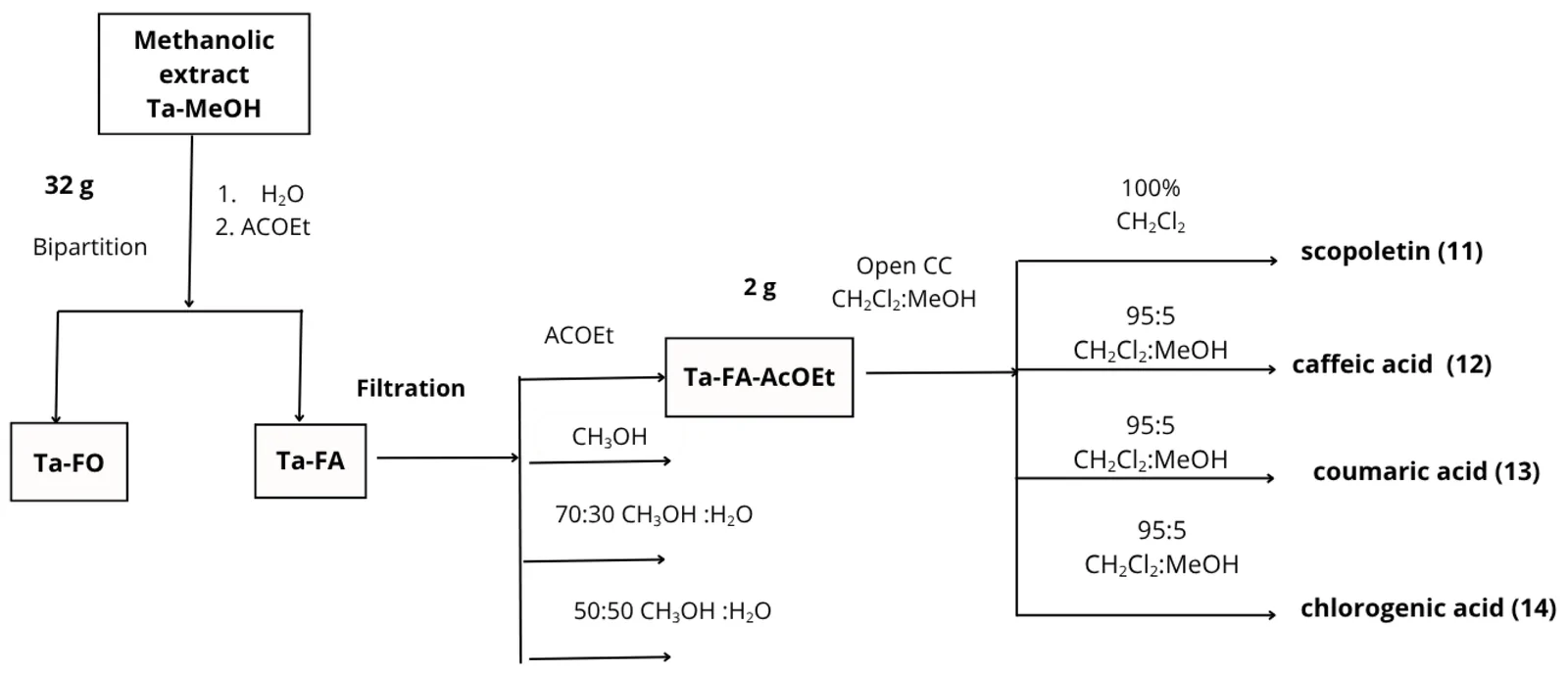
Strategy for the separation of compounds (**11**–**14**).

A total of 79.6 g of methanolic extract (Ta-MeOH) was dissolved in 200 mL of water and subjected to a bipartition using 200 mL of ethyl acetate to obtain two phases: the Ta-FO ethyl acetate phase and the Ta-FA aqueous phase. A 8 g quantity of Ta-FA was filtered using the following systems: 100% AcOEt, CH_3_OH:H_2_O 70:30, CH_3_OH:H_2_O 50:50. A 2 g quantity of the fraction was adsorbed onto 3 g of silica gel and loaded onto a 20 g normal phase column, yielding 36 fractions of 25 mL each. In fractions 2–5 (CH_2_Cl_2_, 100%), a white precipitate formed. This was washed and filtered with CH_3_OH and was found to be soluble in CH_2_Cl_2_. Under long-wave UV, a blue color characteristic of coumarins was observed, and it was confirmed to be scopoletin (**11**). In fractions 6–11 (CH_2_Cl_2_:CH_3_OH 95:5), compounds (**12**) to (**14**) were isolated. Fraction 6 yielded a white solid, caffeic acid (**12**), while fractions 7–8 yielded a white solid, which was isolated and characterized as coumaric acid (**13**). A white solid isolated from fractions 11–12 was identified as chlorogenic acid (**14**). This process is summarized in Figure 9.

#### 4.3.3. Acetylation Reaction

Reverse-phase chromatography of Ta-R4 fractions 18–27, corresponding to fractions 13–15 (50 mg, 90:10 H_2_O:CH_3_CN), showed similarity in their TLC analysis; to reduce its complexity, an acetylation reaction was therefore performed; 50 mg of the compound was taken, and 3 mL of acetic anhydride and 1 mL of pyridine were added. The mixture was stirred for 30 min. After this time, once the reaction had been confirmed complete by TLC analysis, the organic phase was extracted with 30 mL of ethyl acetate and washed 3 times with 30 mL of water. The organic phase (ethyl acetate) was then separated and dried to obtain 80 mg of the organic phase (ethyl acetate fraction). The result of this acetylation reaction after performing the chromatographic separation of the reaction crude was 4α-terpineol sambubioside hexaacetate (**7a**).

### 4.4. Molecular Docking

Molecular docking studies were carried out using the Molecular Operating Environment (MOE, version 2019.01). The crystal structures of MAO-A with harmine (PDB: 2Z5X, 2.20 Å) and MAO-B with safinamide (PDB: 2V5Z, 1.60 Å) [34] were retrieved from the Protein Data Bank. The protein structures were prepared using the QuickPrep module implemented in MOE, which assigns bond orders, adds hydrogen atoms, predicts protonation states, and performs a restrained energy minimization using the Amber10:EHT force field. The flavin adenine dinucleotide (FAD) cofactor and crystallographic water molecules were retained throughout the docking calculations because crystallographic water molecules are important for protein–ligand interactions [76,77,78].

Compounds (**1**–**9**) and (**11**–**14**) were drawn in ChemDraw Professional (Version 15.0.0.106, PerkinElmer Informatics, Inc.), exported as sdf files, imported into MOE, and energy minimized before docking. The binding site was defined using the coordinates of the co-crystallized ligand through the Ligand Atoms option. Docking calculations were performed using the Triangle Matcher placement method with the London dG scoring function for the initial ranking of poses. Pose refinement was carried out using the Rigid Receptor protocol, and the final poses were rescored using the GBVI/WSA dG scoring function. For each ligand, 100 docking poses were generated, and the 10 best-ranked poses were retained for further evaluation. The most appropriate binding pose was selected based on its docking score and binding orientation within the active site [79].

The docking protocol was validated by redocking the co-crystallized ligands into their respective binding sites. The protocol successfully reproduced the experimental binding modes, yielding RMSD values of 0.7673 Å for harmine in MAO-A and 0.9959 Å for safinamide in MAO-B, confirming the reliability of the docking procedure. Protein–ligand interactions were analyzed using the Ligand Interactions and Surface Maps tools implemented in MOE and The PyMOL Molecular Graphics System, Version 3.0 Schrödinger, LLC. Hydrogen bonds and other non-covalent interactions were identified according to the geometric criteria implemented in the software, and the interaction profiles were used to support the interpretation of the predicted binding modes.

### 4.5. Biological Model

#### 4.5.1. Reagents and Drugs

Reserpine (RES) and L-3,4-dihydroxyphenylalanine, Levodopa (L-DOPA) were purchased from Sigma Aldrich, Naucalpan de Juárez, Mexico. All other reagents were of analytical grade obtained from companies that certify their purity and quality.

#### 4.5.2. Animals

Two-month-old male CF-1 mice (weighing 25–35 g) were obtained from Productora Nacional de Biológicos Veterinarios S.A. (PRONABIVE), Mexico City, Mexico. The animals were kept in polycarbonate cages in a temperature-controlled room (22 ± 2 °C) with a 12 h light/dark cycle (lights on at 7 a.m.); food and water were provided ad libitum.

In accordance with this standard and international specifications, the animals underwent a health check. In compliance with current regulations, the reception and maintenance of the animals are supervised by a veterinary zootechnician, who ensures that all admitted animals are suitable for experimentation. This supervision period lasts 10 days after the mice arrive and before the experiment. Any animal showing a general decline in health is reported to the supplier and is not allowed access to the experimental laboratories. The study complies with national and international research regulations. This protocol was submitted to and approved by the Ethics Committee of the IMSS Biomedical Research Center; it was granted registration number R-CICUAL-2024-10 and registration with the local committee of investigation R-2025-1702-002. The handling of laboratory animals was carried out in accordance with the Mexican Official Standard NOM-062-ZOO-1999, on techniques for the care and use of laboratory animals. All animals arriving at the laboratory have been previously evaluated for their health. Those that do not meet the criteria of good weight, normal behavior, consistent fecal boluses, adequate skin color, among other health variables, were not considered in the trial and were also separated from the other animals without allowing access to the experimental laboratory.

#### 4.5.3. Experimental Design and Treatment

**Figure 10:**
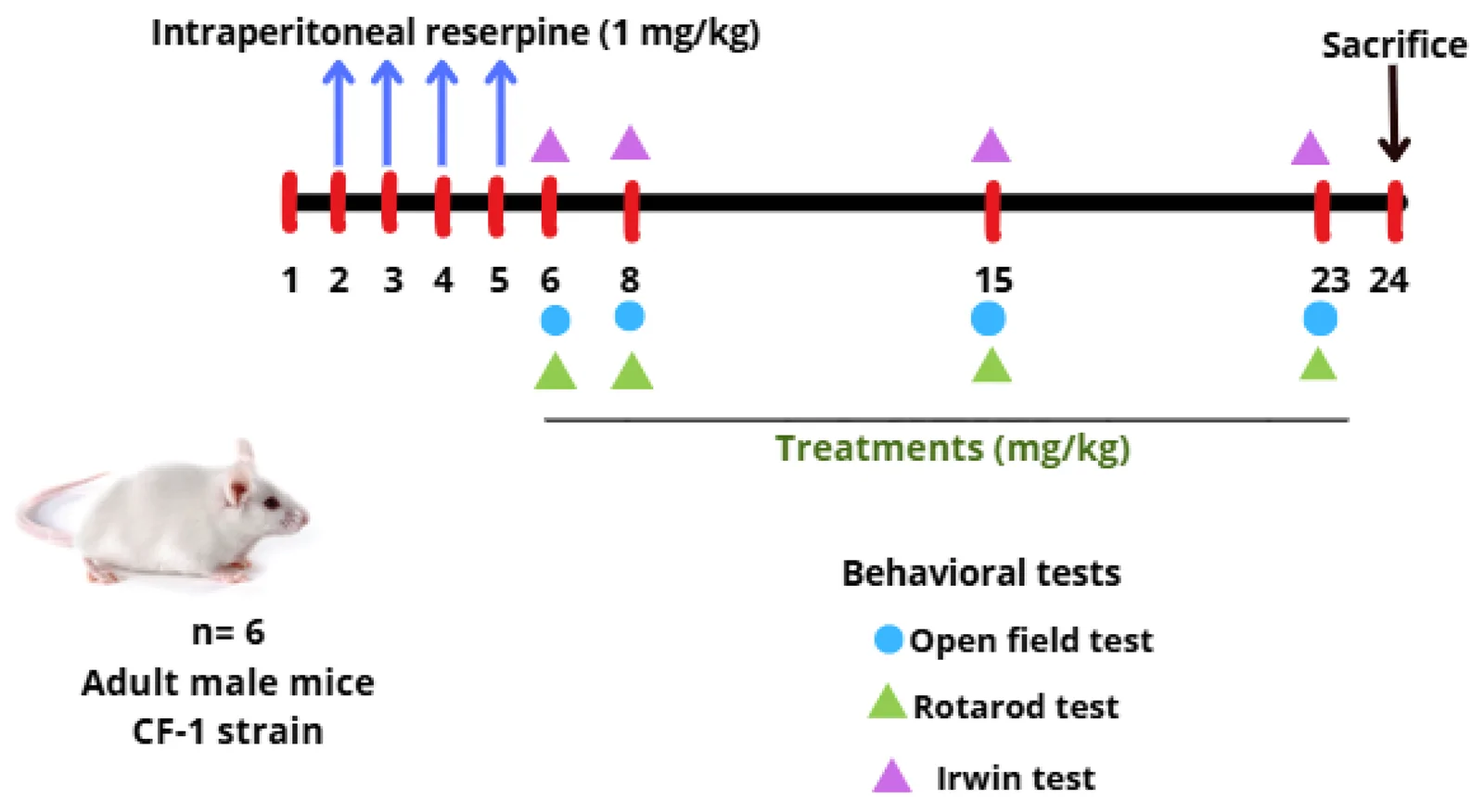
Experimental design of the experimental Parkinson’s model induced by reserpine (RES).

This protocol was adapted from [80] as shown in Figure 10. Mice were randomly assigned to different experimental groups (n = 6 per group). Mice were randomized, using the method in Excel with the RAND function (each subject was assigned a random number, and those numbers then sorted the list to eliminate selection bias), into groups (*n* = 6), the selection of the number of animals to be used was based on other publications made by the group, and taking into consideration the 3Rs: Replacement, Reduction, and Refinement, with in silico analysis first used to select the treatments for the in vivo model [81]. In this trial, males were selected, which is recognized as a limitation due to sex bias; the decision was based on the laboratory’s approach and the accessibility of these animals.

The damage group received reserpine (RES) intraperitoneally (1 mg/kg) every 24 h for four consecutive days (days 2, 3, 4, 5). All groups except the healthy group (BASAL) were damaged with RES, which was administered intraperitoneally (1 mg/kg) (days 2, 3, 4, 5). The negative control group (BASAL) received an intraperitoneal injection of 100 µL/10 g of weight of saline solution (0.9% NaCl). The positive control group was administered the standard treatment for Parkinson’s disease, L-DOPA (150 mg/kg), this dose was chosen according to what has been reported in the literature [82]. The L-DOPA doses were administered orally from day 6 to day 24; no other drug was co-administered with it. From day 6, the animals received daily treatment for 18 days with one of the experimental compounds: methanolic extract (Ta-MeOH, 100 mg/kg); fractions Ta-R1, Ta-R3, Ta-R4 (25 mg/kg each); or β-sitosterol glucoside (**6**) and tiliroside (**8**) (1 mg/kg). The doses of extracts and fractions were based on the research group’s experience in previous studies on the plant [24] and, for the pure compounds, on previous work [28] on optimal doses to observe activity. To improve sample solubility, 2% Tween was used; both the healthy and damaged groups orally received a solution containing only this vehicle.

The treatments were labeled with a code, concentration, preparation date, and the user who prepared them. The animal boxes were labeled with the following information: number of animals, strain, sex, date of arrival at the animal facility, treatment group, and registered protocol number. Each animal was marked in accordance with the official Mexican standard NOM-062-ZOO-1999 and international standards.

Behavioral tests were administered 1 h after treatment. The researcher responsible for the pharmacological component of the project, along with the researcher administering the treatments to the animals and the technician in charge of caring for, changing, and feeding the rodents, are familiar with the labeling, the type of experiment, and the project name and registration number associated with each design

Behavioral assessments were conducted on days 6, 8, 15, and 23. On day 24, all animals were sacrificed. Mice were anesthetized with sodium pentobarbital and euthanized by cervical dislocation.

#### 4.5.4. Behavioral Tests

The animals were housed and acclimated to the experimental room 1 h prior to testing. All tests were conducted in an experimentation room illuminated with the standard laboratory lighting, which was used in the animal housing/experimental room.

##### Irwin Test

This test is a systematic observational procedure of general behavior and allows for the assessment of neurotoxicity through various symptoms present in the experimental animals. The observed parameters were abdominal contortion, raised fur, palpebral ptosis, movement alterations, piling, loss of muscle tone, tremors, limb paralysis, salivary secretion, convulsions, constipation and stool retention.

##### Open Field Test (OFT)

The OFT test, conducted to measure spontaneous motor coordination, consisted of placing the rodent inside an acrylic box with transparent walls and a black floor (30 × 30 × 15 cm) divided into 9 squares. For 5 min, the following were recorded: total crossings (TC).

Because the response was assessed longitudinally, the area under the curve (AUC) was calculated for each animal using the trapezoidal method to provide a single quantitative measure of the overall locomotor response. For each animal, the observation days (6, 8, 15, and 23) were used as the x-axis, and the total number of crossings was used as the y-axis. The resulting AUC values were subsequently used as the individual values for statistical analysis. The results are presented as mean AUC ± SD for each experimental group.

##### Motor Coordination Test in Rota-Rod Apparatus

To evaluate the animals’ motor skills and motor coordination, the Rota-Rod apparatus was used. This equipment consists of a 3 cm diameter rotating rod divided into five 6 cm wide tracks, a timer that stopped when the mouse fell onto a track, and software that recorded the dwell time.

The mouse was placed on the moving cylinder for up to 1 min, during which it had to coordinate its lower limbs to maintain balance on the rotating cylinder; the time until the mouse fell was recorded. Before the first test, a training trial was conducted at 4 rpm/min. After this training, the Rota-Rod was used at 4 rpm/min, then increased to 10 and 20 rpm/min. The mice were allowed to rest for 5 min between each change in speed.

Because the response was evaluated over time, the area under the curve (AUC) was calculated for each animal to provide a single quantitative measure of the overall response. A separate value was obtained for each rotation speed (4, 10, and 20 rpm), with the observation days (6, 8, 15, and 23) used as the x-axis and latency to fall (s) as the y-axis. The trapezoidal method was used for the calculation. Individual values were subsequently used for statistical analysis, and the results are presented as mean AUC ± SD for each experimental group. The graphical presentation of latency to fall and its corresponding AUC was consistent with previously reported approaches in Rota-Rod studies [83].

### 4.6. Euthanasia

The animals were euthanized with an overdose of sodium pentobarbital (100 mg/kg i.p.), followed by cervical dislocation (NOM-062-ZOO-1999).

### 4.7. Statistical Analysis

The area under the curve (AUC) was calculated individually for each animal from the behavioral measurements obtained throughout the study. The resulting individual AUC values (*n* = 6 per group) were subjected to one-way ANOVA followed by Dunnett’s multiple comparisons test using SPSS.

The researcher, together with the data analyst, created a matrix that was interpreted using SPSS; the results of the model were subjected to a one-way ANOVA followed by a Dunnett’s post-test with a significance level of 0.05, comparing the experimental treatments, positive control, and healthy animals with the RES group (damage without treatment). All analyses were processed using the SPSS 11.0 statistical package.

## 5. Conclusions

The present study showed that *T. americana* attenuated behavioral alterations and motor deficits in a reserpine-induced mouse model of parkinsonism. Molecular docking served as a computational approach to prioritize compounds for biological evaluation, leading to the selection of β-sitosterol glucoside (**6**) and tiliroside (**8**), which exhibited distinct behavioral activity profiles in vivo.

Because compound (**7**), 4α-terpineol sambubioside, represents a novel diglycosylated monoterpene and this class of metabolites has received little attention in previous pharmacological studies, further efforts should be directed toward optimizing its isolation and biological evaluation. Although β-sitosterol glucoside (**6**) and tiliroside (**8**) displayed favorable predicted binding modes toward MAO-B and beneficial effects in the reserpine model, the molecular mechanisms underlying these responses remain to be established. Therefore, additional biochemical and pharmacological studies are warranted to clarify their molecular targets and to further explore the therapeutic potential of these metabolites and *T. americana* as a source of bioactive compounds for the future development of phytomedicines.

## Figures and Tables

**Table 1:** ^1^H (500 MHz, CDCl_3_) and ^13^C (125 MHz, CDCl_3_) NMR spectral data of compound (**7a**).

Position	δ ^13^C (ppm)	δ ^1^H (ppm, *J* in Hz)
1	134.32	
2	120.44	5.34 (s, br)
3a b	26.79	1.72 (m) 1.99 (m)
4	44.12	1.57 (tdd, 12.0, 4.7, 2.2 Hz)
5a b	23.56	1.84 (m) 1.86 (m)
6a b	31.00	1.90 (m) 1.92 (m)
7	23.47	1.62(s, br)
8	80.62	
9	22.04	1.11 (s)
10	25.22	1.18 (s)
Glucopyranosyl		
1′	95.20	δ 4.66 (d, 8.0)
2′	71.65	4.90 (m)
3′	73.20	5.19 (t, 9.5)
4′	68.79	4.92 (m)
5′	73.14	3.63 (dd, 9.8, 1.9).
6′a b	67.65	3.76 (dd, 10.7, 1.7) 3.59 (m)
Xylopyranosyl		
1″	100.17	4.55 (d, 6.5)
2″	70.54	4.87 (dd, 8.3, 6.5)
3″	71.25	5.10 (dd, 8.1, 8.2)
4″	69.25	4.91(m)
5″a b	61.89	3.34 (dd, 12.0, 8.4) 4.13 (dd,11.9, 4.9)

**Table 2:** Docking scores of the chemical compounds (**1**–**9**), (**11**–**14**) from the methanolic extract of *Tilia americana*.

Compound	Docking Scores (Kcal/mol) MAO-A	Docking Scores (Kcal/mol MAO-B)
α-amyrine (**1**)	−4.4852	−3.4841
β-amyrine (**2**)	−3.5883	−1.7231
β-sitosterol (**3**)	−5.7531	−8.0385
Stigmasterol (**4**)	−5.7954	−7.2364
Ursolic acid (**5**)	−4.1396	−3.463
β-sitosterol glucoside (**6**)	−2.2351	−10.8733
4α-terpineol sambubioside (**7**)	−7.8239	−11.1434
Tiliroside (**8**)	−7.3688	−12.6986
Rutin (**9**)	−6.996	−12.9474
Scopoletin (**11**)	−6.4344	−6.2714
Caffeic acid (**12**)	−6.1557	−6.2535
Coumaric acid (**13**)	−5.7898	−5.855
Chlorogenic acid (**14**)	−7.0579	−8.6505
Reference compounds		
Harmine	−8.2358	
Safinamide		−7.2729

**Table 3:** Interaction/amino acid with MAO-B diagrams for compounds (**6**) and (**8**).

Compound Name	Interaction/Amino Acid
Safinamide (Reference compound)	Polar: Cys397, Gly57, Cyst172, Gly58. Greasy: Trp388, Phe343, Ile198, Phe168, Leu56, Basic: Arg42, Lys296 Receptor exposure and polar: Tyr398, Gly,58, Tyr435, Gln206 Receptor exposure and greasy: Ile199, Leu171, Phe 343 Side chain acceptor: Cys172 Backbone chain acceptor: Ile198 Arene-H: Leu171
β-sitosterol glucoside (**6**)	Polar: Tyr 326, Cys172, Gly434, Ser59, Gly57, Thr43, Cys397, Tyr60. Greasy: Pro102, Pro103, Pro 104, Leu164, Ile316, Trp119, Phe168, Leu167, Met436. Basic: Arg42. Receptor exposure and polar: Gln206, Tyr435, Gly58, Tyr398. Receptor exposure and greasy: Ile199, Leu171. Backbone donor: Pro102
Tiliroside (**8**)	Polar: Cys397, Tyr326, Gly205, Cys172, Gly57, Thr426, Ser59, Thr43. Greasy: Val 294, Trp388, Leu56, Val61, Ala439, Met436, Phe168, Ile198. Receptor exposure and Basic: Lys 296. Receptor exposure and polar: Gln205, Tyr398, Tyr435, Tyr60, Gly58. Receptor exposure and greasy: Ile199, Leu171. Backbone donor: Tyr60. Solvent contact: Lys296. Arene-H: Ile199.

**Table 4:** Irwin test: Behavioral changes induced with RES in control groups.

Treatments (mg/kg)	BASAL				RES (1.0)				L-DOPA (150) + RES (1.0)			
	Observation Days											
Evaluated Parameter	6	8	15	23	6	8	15	23	6	8	15	23
Abdominal contortion	-	-	-	-	+++	+++	++	++	+++	++	+	-
Rising hair	-	-	-	-	+++	+++	++	++	+++	++	+	-
Palpebral ptosis	-	-	-	-	+++	+++	++	++	+++	++	+	-
Altered movements	-	-	-	-	+++	+++	++	++	+++	++	++	-
Huddling	-	-	-	-	+++	+++	+++	+++	+++	++	+	-
Loss of muscle tone	-	-	-	-	+++	+++	++	++	+++	+++	++	-
Tremors	-	-	-	-	-	-	-	-	-	-	-	-
Limb paralysis	-	-	-	-	-	-	-	-	-	-	-	-
Salivary secretion	-	-	-	-	-	-	-	-	-	-	-	-
Seizures	-	-	-	-	-	-	-	-	-	-	-	-
Stomach distension	-	-	-	-	+++	++	+	-	+++	++	+	-
Constipation	-	-	-	-	+++	+++	++	++	+++	++	+	-
Stereotyped behaviors	-	-	-	-	-	-	-	-	-	-	-	+

Absence -; Mild +; Moderate ++; Severe +++. BASAL = healthy group; RES = damage group; L-DOPA = drug control group.

**Table 5:** Irwin test: Behavioral changes induced with RES in treated groups (extract and fractions).

Treatments (mg/kg)	RES (1.0)				Ta-MeOH (100)				Ta-R1 (25)				Ta-R3 (25)				Ta-R4 (25)			
Evaluated Parameter					Observation Days															
	6	8	15	23	6	8	15	23	6	8	15	23	6	8	15	23	6	8	15	23
Abdominal contortion	+++	+++	++	++	+++	++	++	+	+++	+++	++	-	+++	+++	+	-	+++	+++	++	+
Rising hair	+++	+++	++	++	+++	++	+	+	+++	++	+	-	+++	+++	+	+	+++	++	++	+
Palpebral ptosis	+++	+++	++	++	+++	++	+	-	+++	++	+	-	+++	+++	+	-	+++	++	+	-
Altered movements	+++	+++	++	++	+++	+++	+	+	+++	+++	++	+	+++	+++	++	+	+++	+++	+	-
Huddling	+++	+++	+++	+++	+++	+++	++	+	+++	+++	++	-	+++	+++	++	+	+++	+++	++	+
Loss of muscle tone	+++	+++	++	++	+++	+++	++	+	+++	+++	++	+	+++	+++	++	+	+++	+++	++	+
Tremors	-	-	-	-	-	-	-	-	-	-	-	-	-	-	-	-	-		-	-
Limb paralysis	-	-	-	-	-	-	-	-	-	-	-	-	-	-	-	-	-	-	-	-
Salivary secretion	-	-	-	-	-	-	-	-	-	-	-	-	+++	+++	++	+	-	-	-	-
Seizures	-	-	-	-	-	-	-	-	-	-	-	-	+++	+++	++	+	-	-	-	-
Stomach distension	+++	++	+	-	+++	++	+	-	+++	+++	++	-	-	-	-	-	+++	++	+	-
Constipation	+++	+++	++	++	+++	++	+	-	+++	++	+	-	-	-	-	-	+++	++	+	-
Stereotyped behaviors	-	-	-	-	-	-	-	-	-	-	-	-	-	-	-	-	-	-	-	-

Absence -; Mild +; Moderate ++; Severe +++. Experimental treatment groups: RES = damage group; Ta-MeOH = methanolic extract from *T. americana*; Ta-R1 = Fraction 1; Ta-R3 = Fraction 3; Ta-R4 = all from Ta-MeOH. **Note:** All treatment groups received RES (1.0 mg/kg).

**Table 6:** Irwin test: Behavioral changes induced with RES in treated groups (compounds 6 and 8).

Treatments (mg/kg)	RES (1.0)				6 (1.0)				8 (1.0)			
Evaluated Parameter	Observation Days											
	6	8	15	23	6	8	15	23	6	8	15	23
Abdominal contortion	+++	+++	++	++	+++	++	++	+	+++	++	-	-
Rising hair	+++	+++	++	++	+++	++	+	+	+++	++	-	-
Palpebral ptosis	+++	+++	++	++	+++	++	-	-	+++	++	-	-
Altered movements	+++	+++	++	++	+++	+++	+	-	+++	++	+	+
Huddling	+++	+++	+++	+++	+++	+++	+	-	+++	+++	+	-
Loss of muscle tone	+++	+++	++	++	+++	+++	+	-	+++	+++	++	+
Tremors	-	-	-	-	-	-	-	-	-	-	-	-
Limb paralysis	-	-	-	-	-	-	-	-	-	-	-	-
Salivary secretion	-	-	-	-	-	-	-	-	-	-	-	-
Seizures	-	-	-	-	-	-	-	-	-	-	-	-
Stomach distension	+++	++	+	-	+++	++	+	-	+++	++	-	-
Constipation	+++	+++	++	++	+++	++	+	-	+++	++	-	-
Stereotyped behaviors	-	-	-	-	-	-	-	-	-	-	-	-

Absence -; Mild +; Moderate ++; Severe +++. Experimental treatment groups: RES= damage group; (**6**) = β-Sitosterol glucoside; (**8**) = Tiliroside. **Note:** All treatment groups received RES (1.0 mg/kg).

## Data Availability

The data supporting the findings of this study are available from the corresponding author upon reasonable request.

## Supplementary Materials

The following supporting information can be downloaded at: https://www.mdpi.com/article/10.3390/molecules31162874/s1, Figure S1. ^1^H NMR (CDCl_3_, 600 MHz) of a mixture of α- and β-amyrine (**1**) and (**2**); Figure S2. ^13^C NMR (CDCl_3_, 150 MHz) of a mixture of α- and β-amyrine (**1**) and (**2**); Figure S3. Mass Spectrum of a mixture of α- and β-amyrine (**1**) and (**2**); Figure S4. ^1^H NMR (CDCl_3_, 600 MHz) of a mixture of β-sitosterol (**3**) and stigmasterol (**4**); Figure S5. ^13^C NMR (CDCl_3_, 150 MHz) of a mixture of β-sitosterol (**3**) and stigmasterol (**4**); Figure S6. Mass Spectrum of a mixture of β-sitosterol (**3**) and stigmasterol (**4**); Figure S7. ^1^H NMR (CDCl_3_, 600 MHz) of ursolic acid (**5**); Figure S8. ^13^C NMR (CDCl_3_, 150 MHz) of ursolic acid (**5**); Figure S9. Mass Spectrum of ursolic acid (**5**); Figure S10. ^1^H NMR (DMSO-d6, 600 MHz) of β-sitosterol glucoside (**6**); Figure S11. ^13^C NMR (DMSO-d6, 150 MHz) of β-sitosterol glucoside (**6**); Figure S12. Mass Spectrum of β-sitosterol glucoside (**6**); Figure S13. ^1^H NMR (CD_3_OD, 600 MHz) of 4α-terpineol sambubioside hexaacetate (**7a**); Figure S14. DEPT NMR (CD_3_OD, 150 MHz) of 4α-terpineol sambubioside hexaacetate (**7a**); Figure S15. COSY NMR (CD_3_OD, 600 MHz) of 4α-terpineol sambubioside hexaacetate (**7a**); Figure S16. TOCSY NMR (CD_3_OD, 600 MHz) of 4α-terpineol sambubioside hexaacetate (**7a**); Figure S17. HSQC NMR (CD_3_OD, 600 MHz) of 4α-terpineol sambubioside hexaacetate (**7a**); Figure S18. HMBC NMR (CD_3_OD, 600 MHz) of 4α-terpineol sambubioside hexaacetate (**7a**); Figure S19. Mass Spectrum of 4α-terpineol sambubioside hexaacetate (**7a**); Figure S20. Mass Spectrum of 4α-terpineol sambubioside hexaacetate (**7a**); Figure S21. HR-ESI-MS of 4α- terpineol sambubioside hexaacetate (**7a**); Figure S22. ^1^H NMR (MeOD, 600 MHz) of tiliroside (**8**); Figure S23. DEPT NMR (MeOD, 150 MHz) of tiliroside (**8**); Figure S24. COSY NMR (MeOD, 600 MHz) of tiliroside (**8**); Figure S25. HSQC NMR (MeOD, 600 MHz) of tiliroside (**8**); Figure S26. HSQC NMR (MeOD, 600 MHz) of tiliroside (**8**); Figure S27. HSQC NMR (MeOD, 600 MHz) of tiliroside (**8**); Figure S28. Mass Spectrum of tiliroside **(8)**; Figure S29. ^1^H NMR (CD_3_OD, 600 MHz) of rutine (**9**); Figure S30. ^13^C NMR (CD_3_OD, 600 MHz) of rutine (**9**); Figure S31. Mass Spectrum of rutine (**9**); Figure S32. ^1^H NMR (DMSO-d6, 600 MHz) of sucrose (**10**); Figure S33. ^13^C NMR (DMSO-d6, 600 MHz) of sucrose (**10**); Figure S34. Mass Spectrum of sucrose (**10**); Figure S35. Mass Spectrum of scopoletin (**11**); Figure S36. Mass Spectrum of caffeic acid (**12**); Figure S37. Mass Spectrum of coumaric acid **(13**); Figure S38. ^1^H NMR (CD_3_OD, 600 MHz) of chlorogenic acid (**14**); Figure S39. ^13^C NMR (CD_3_OD, 600 MHz) of chlorogenic acid (**14**); Figure S40. Mass Spectrum of chlorogenic acid (**14**); Table S1. 3D protein-ligand interaction diagrams of the active site of MAO-A and MAO-B with compounds isolated from *Tilia americana* var. *mexicana.*; Table S2. Protein–ligand interaction diagrams of the active site 3D view of the binding interacting with MAO-B and 2D view of binding site interactions diagrams for compounds (**1**–**9**); (**11**–**14**).; Table S3. Interaction/Amino Acid with MAO-B diagrams for compounds from *Tilia americana* var. *mexicana*.

## Abbreviations

The following abbreviations are used in this manuscript:

AcOEt	ethyl acetate
COSY	Correlation Spectroscopy
DEPT	Distortionless Enhancement by Polarization Transfer
ESI-MS	Electrospray Ionization Mass Spectrometry.
FAD	Flavin Adenine Dinucleotide
HMBC	Heteronuclear Multiple Bond Correlation,
HSQC	Heteronuclear Single Quantum Coherence
L-DOPA	Levodopa
MAO-A	Monoamino oxidase A
MAO-B	Monoamino oxidase B
MAOs	Monoamino oxidases
MOE	Molecular Operating Environment
NMR	Nuclear Magnetic Resonance
OFT	Open Field Test
PD	Parkinson Disease
PDB	Protein Data Bank
PRONAVIBE	Productora Nacional de Biológicos Veterinarios S.A.
RES	Reserpine
TC	Total Crossing
TLC	Thin-Layer Chromatography
TOCSY	Totally Correlated Spectroscopy
UV	Ultraviolet
