# Supplementary material for: Phytochemical Characterization of Tilia americana var. mexicana, Exploratory Study of MAO Molecular Docking and Evaluation of Behavior in Mice in a Reserpine-Induced Parkinson Model
Source: Molecules. 2026 Aug 17;31(16):2874. doi: 10.3390/molecules31162874 (PMC13515478; doi:10.3390/molecules31162874)
Supplement: Supplementary file 1 [file molecules-31-02874-s001.zip › molecules-4437551-supplementary.pdf]

molecules

Article

Phytochemical Characterization of *Tilia Americana* var. *mexicana*, Exploratory Study of MAO Molecular Docking and Evaluation of Behavior in Mice in a Reserpine-Induced Parkinson Model

Supplementary data

Contents

Figure S1. ^{1}H NMR ($CDCl_3$, 600 MHz) of a mixture of α- and β-amyrine (**1**) and (**2**)

Figure S2. ^{13}C NMR ($CDCl_3$, 150 MHz) of a mixture of α- and β-amyrine (**1**) and (**2**)

Figure S3. Mass Spectrum of a mixture of α- and β-amyrine (**1**) and (**2**)

Figure S4. ^{1}H NMR ($CDCl_3$, 600 MHz) of a mixture of β-sitosterol (**3**) and stigmasterol (**4**)

Figure S5. ^{13}C NMR ($CDCl_3$, 150 MHz) of a mixture of β-sitosterol (**3**) and stigmasterol (**4**)

Figure S6. Mass Spectrum of a mixture of β-sitosterol (**3**) and stigmasterol (**4**)

Figure S7. ^{1}H NMR ($CDCl_3$, 600 MHz) of ursolic acid (**5**)

Figure S8. ^{13}C NMR ($CDCl_3$, 150 MHz) of ursolic acid (**5**)

Figure S9. Mass Spectrum of ursolic acid (**5**)

Figure S10. ^{1}H NMR (DMSO-d6, 600 MHz) of β-sitosterol glucoside (**6**)

Figure S11. ^{13}C NMR (DMSO-d6, 150 MHz) of β-sitosterol glucoside (**6**)

Figure S12. Mass Spectrum of β-sitosterol glucoside (**6**)

Figure S13. ^{1}H NMR ((CD_3OD, 600 MHz) of 4α-terpineol sambubioside hexaacetate (**7a**)

Figure S14. DEPT NMR (CD_3OD, 150 MHz) of 4α-terpineol sambubioside hexaacetate (**7a**)

Figure S15. COSY NMR (CD_3OD, 600 MHz) of 4α-terpineol sambubioside hexaacetate (**7a**)

Figure S16. TOCSY NMR (CD_3OD, 600 MHz) of 4α-terpineol sambubioside hexaacetate (**7a**)

Figure S17. HSQC NMR (CD_3OD, 600 MHz) of 4α-terpineol sambubioside hexaacetate (**7a**)

Figure S18. HMBC NMR (CD_3OD, 600 MHz) of 4α-terpineol sambubioside hexaacetate (**7a**)

Figure S19. Mass Spectrum of 4α-terpineol sambubioside hexaacetate (**7a**)

Figure S20. Mass Spectrum of 4α-terpineol sambubioside hexaacetate (**7a**)

Figure S21. HR-ESI-MS of 4α- terpineol sambubioside hexaacetate (**7a**)

Figure S22. ^{1}H NMR (MeOD, 600 MHz) of tiliroside (**8**)

Figure S23. DEPT NMR (MeOD, 150 MHz) of tiliroside (**8**)
Figure S24. COSY NMR (MeOD, 600 MHz) of tiliroside (**8**)
Figure S25. HSQC NMR (MeOD, 600 MHz) of tiliroside (**8**)
Figure S26. HSQC NMR (MeOD, 600 MHz) of tiliroside (**8**)
Figure S27. HSQC NMR (MeOD, 600 MHz) of tiliroside (**8**)
Figure S28. Mass Spectrum of tiliroside **(8)**
Figure S29. ^{1}H NMR (CD_3OD, 600 MHz) of rutine (**9**)
Figure S30. ^{13}C NMR (CD_3OD, 600 MHz) of rutine (**9**)
Figure S31. Mass Spectrum of rutine (**9**)
Figure S32. ^{1}H NMR (DMSO-d6, 600 MHz) of sucrose (**10**)
Figure S33. ^{13}C NMR (DMSO-d6, 600 MHz) of sucrose (**10**)
Figure S34. Mass Spectrum of sucrose (**10**)
Figure S35. Mass Spectrum of scopoletin (**11**)
Figure S36. Mass Spectrum of caffeic acid (**12**)
Figure S37. Mass Spectrum of coumaric acid **(13**)
Figure S38. ^{1}H NMR (CD_3OD, 600 MHz) of chlorogenic acid (**14**)
Figure S39. ^{13}C NMR (CD_3OD, 600 MHz) of chlorogenic acid (**14**)
Figure S40. Mass Spectrum of chlorogenic acid (**14**)
Table S1. 3D protein-ligand interaction diagrams of the active site of MAO-A and MAO-B with compounds isolated from *Tilia americana* var. *mexicana.*
Table S2. Protein–ligand interaction diagrams of the active site 3D view of the binding interacting with MAO-B and 2D view of binding site interactions diagrams for compounds (**1 - 9**); (**11 - 14**).
Table S3. Interaction/Amino Acid with MAO-B diagrams for compounds from *Tilia americana* var. *mexicana*

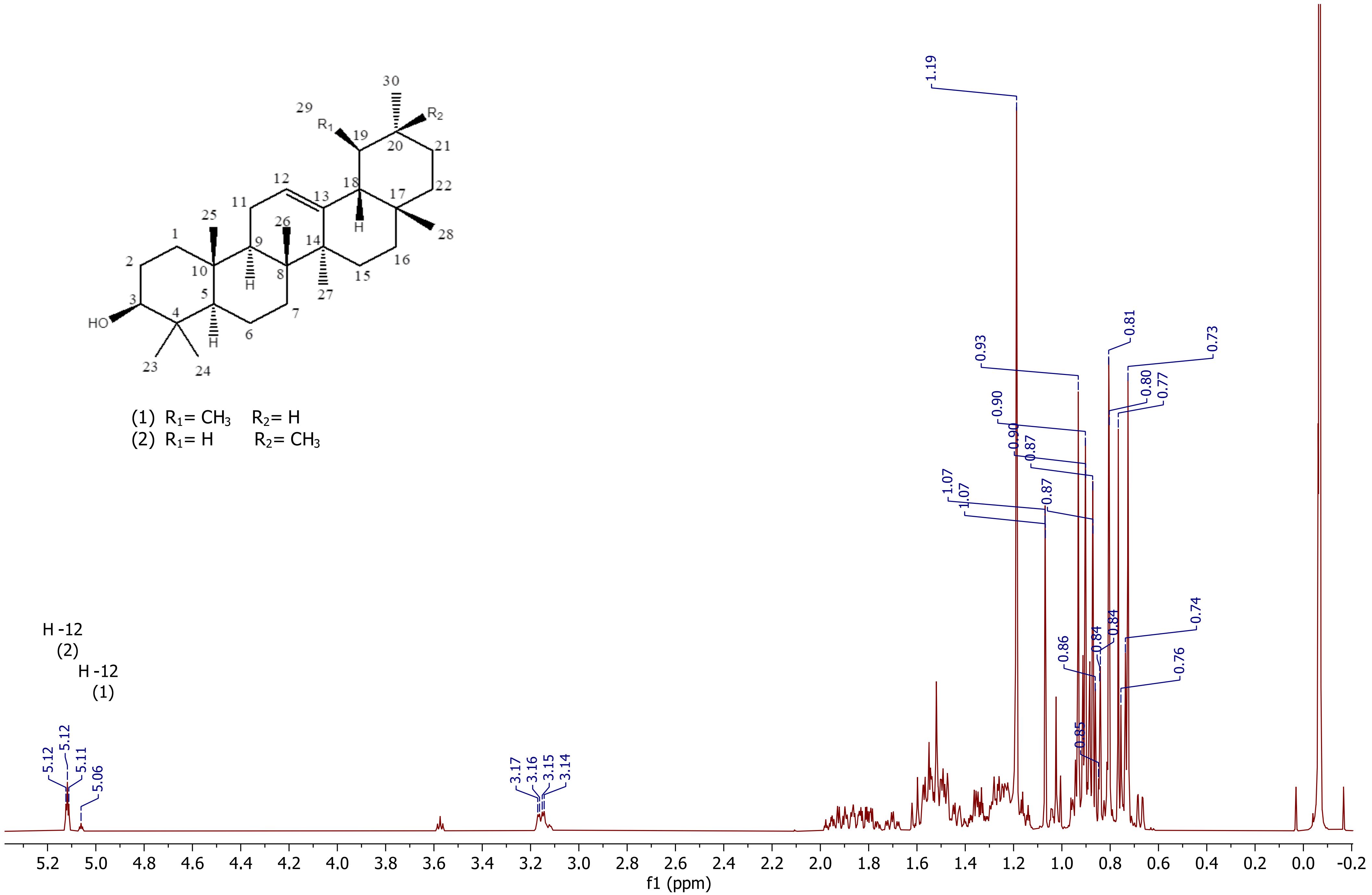

Figure S1. ^{1}H NMR ($CDCl_3$, 600 MHz) of a mixture of α- and β-amyrine (1) y (2)

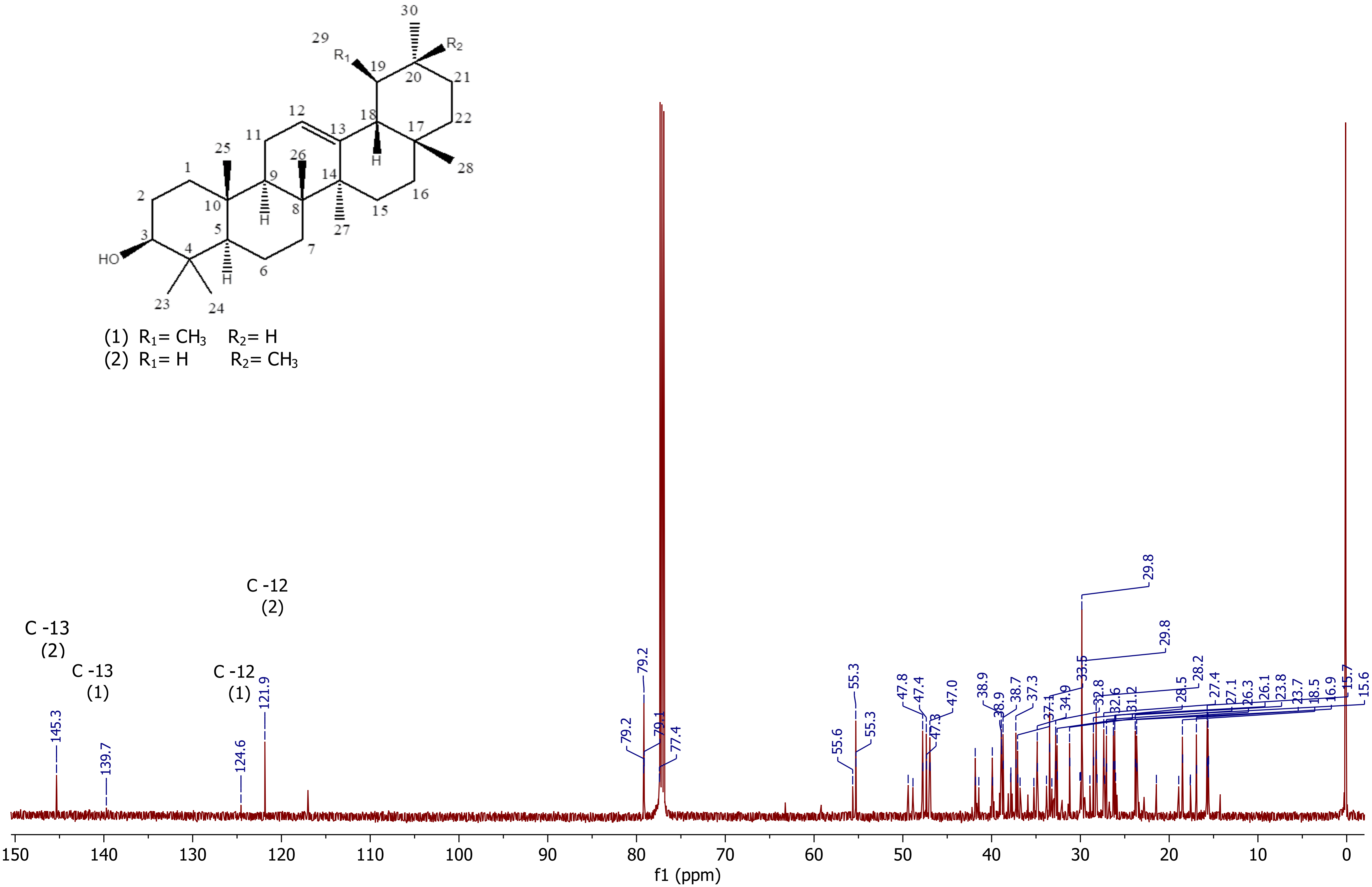

Figure S2. ^{13}C NMR ($CDCl_3$, 150 MHz) of a mixture of α- and β-amyrine (1) y (2)

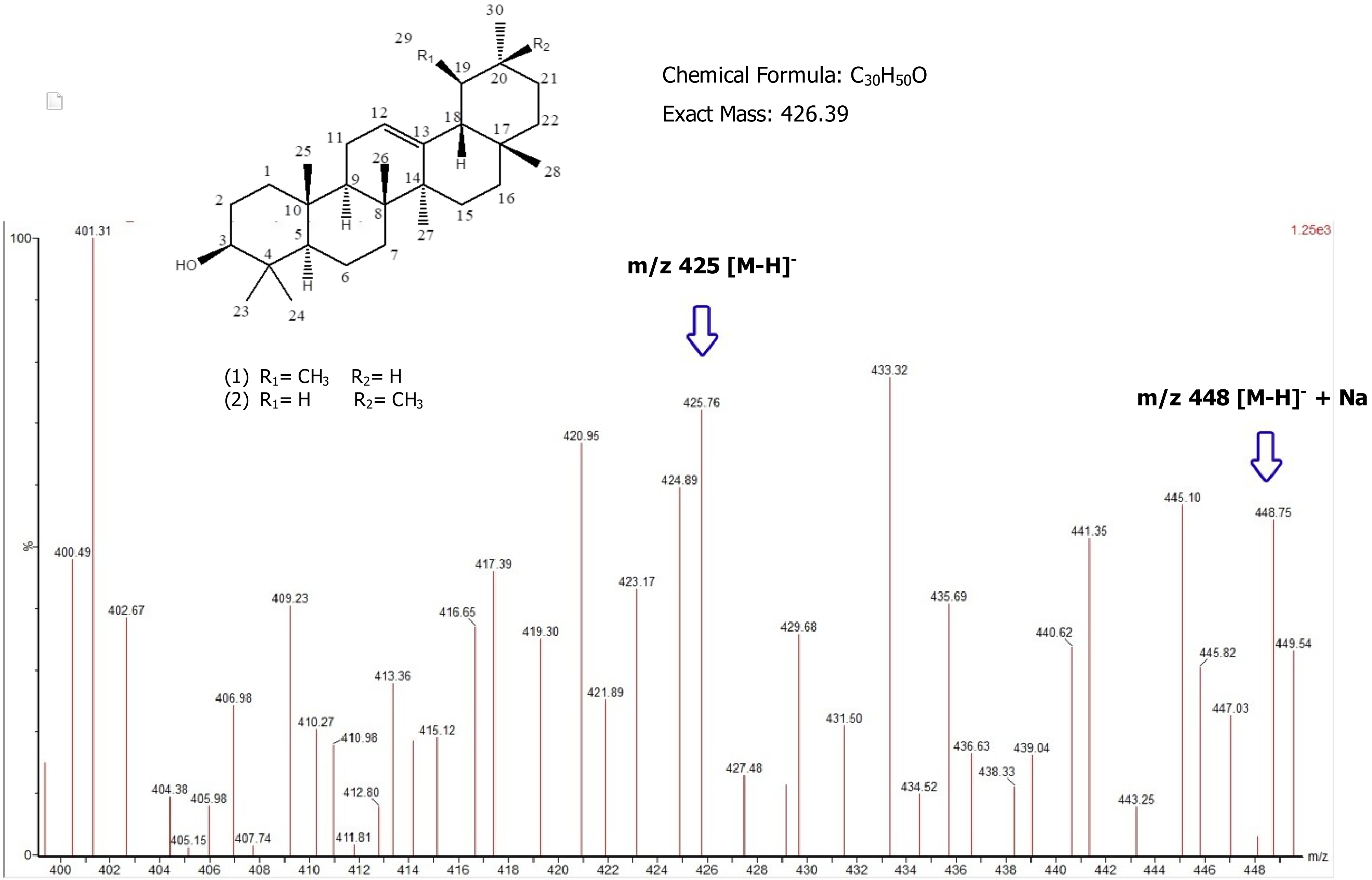

Figure S3. Mass Spectrum of a mixture of α- and β-amyrine (1) and (2)

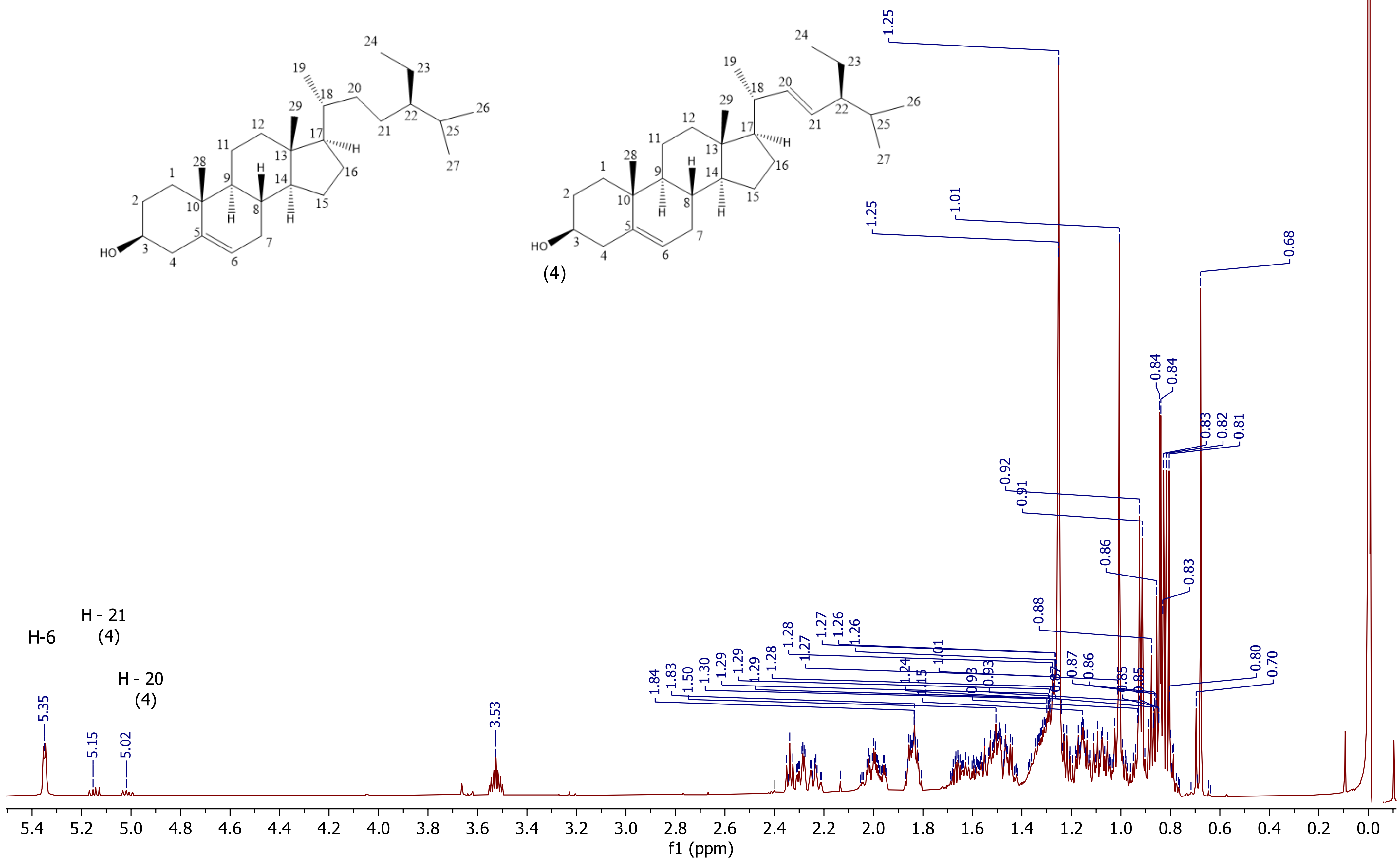

Figure S4. ^{1}H NMR ($CDCl_3$, 600 MHz) of a mixture of β-sitosterol (3) and stigmasterol (4)

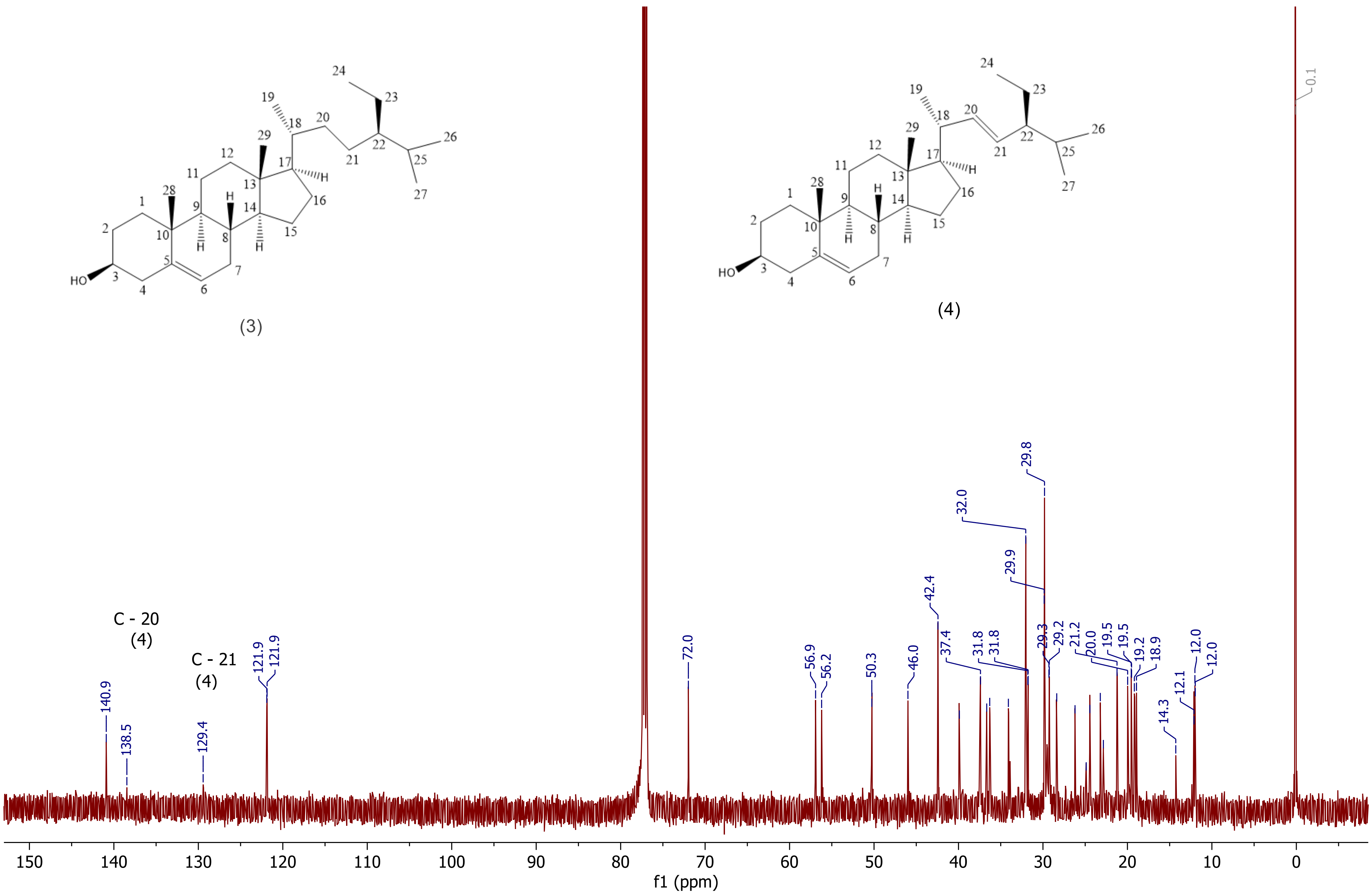

Figure S5. ^{13}C NMR ($CDCl_3$, 150 MHz) of a mixture of β-sitosterol (3) and stigmasterol (4)

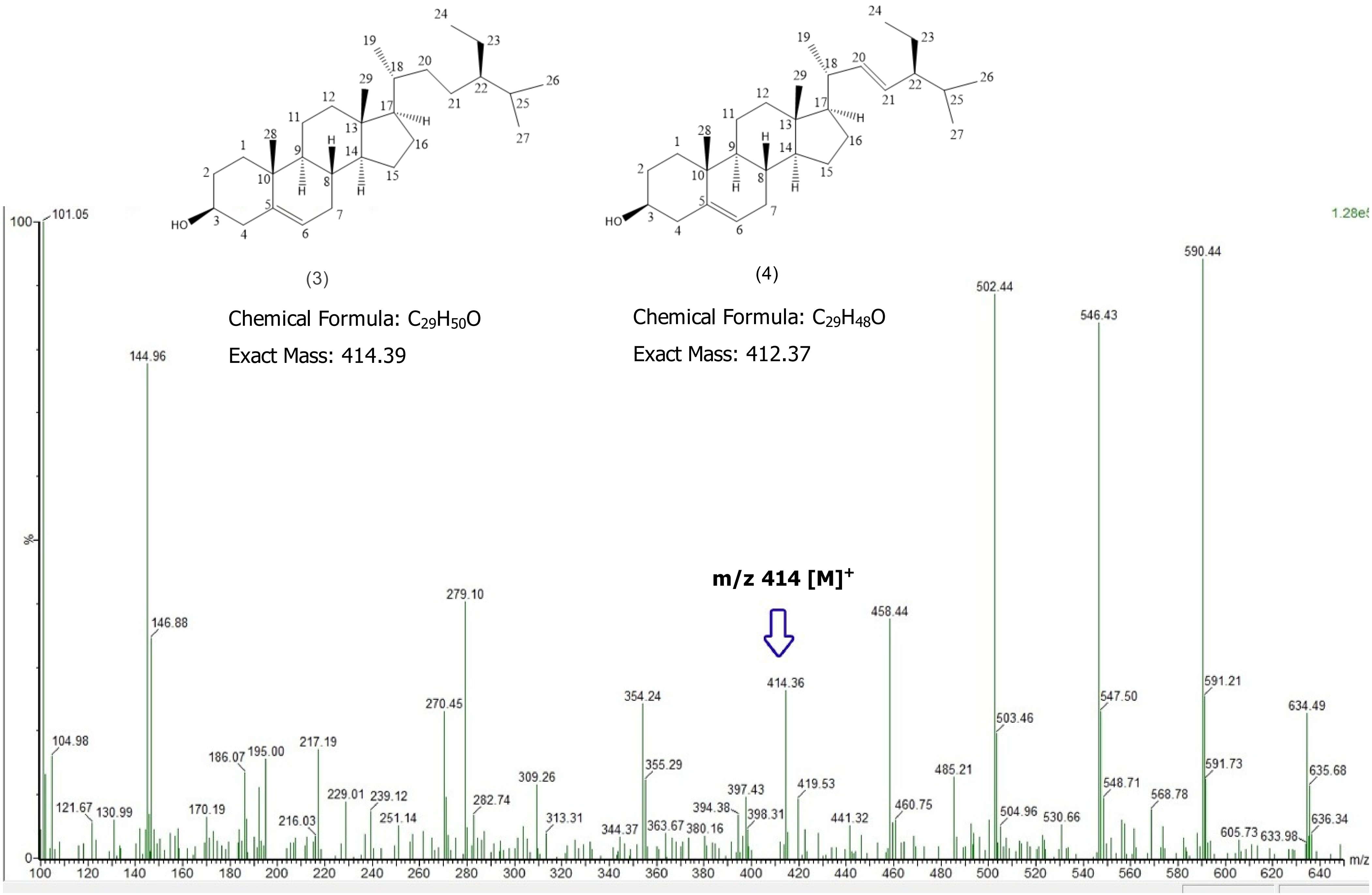

Figure S6. Mass Spectrum of a mixture of β-sitosterol (3) and stigmasterol [illegible]

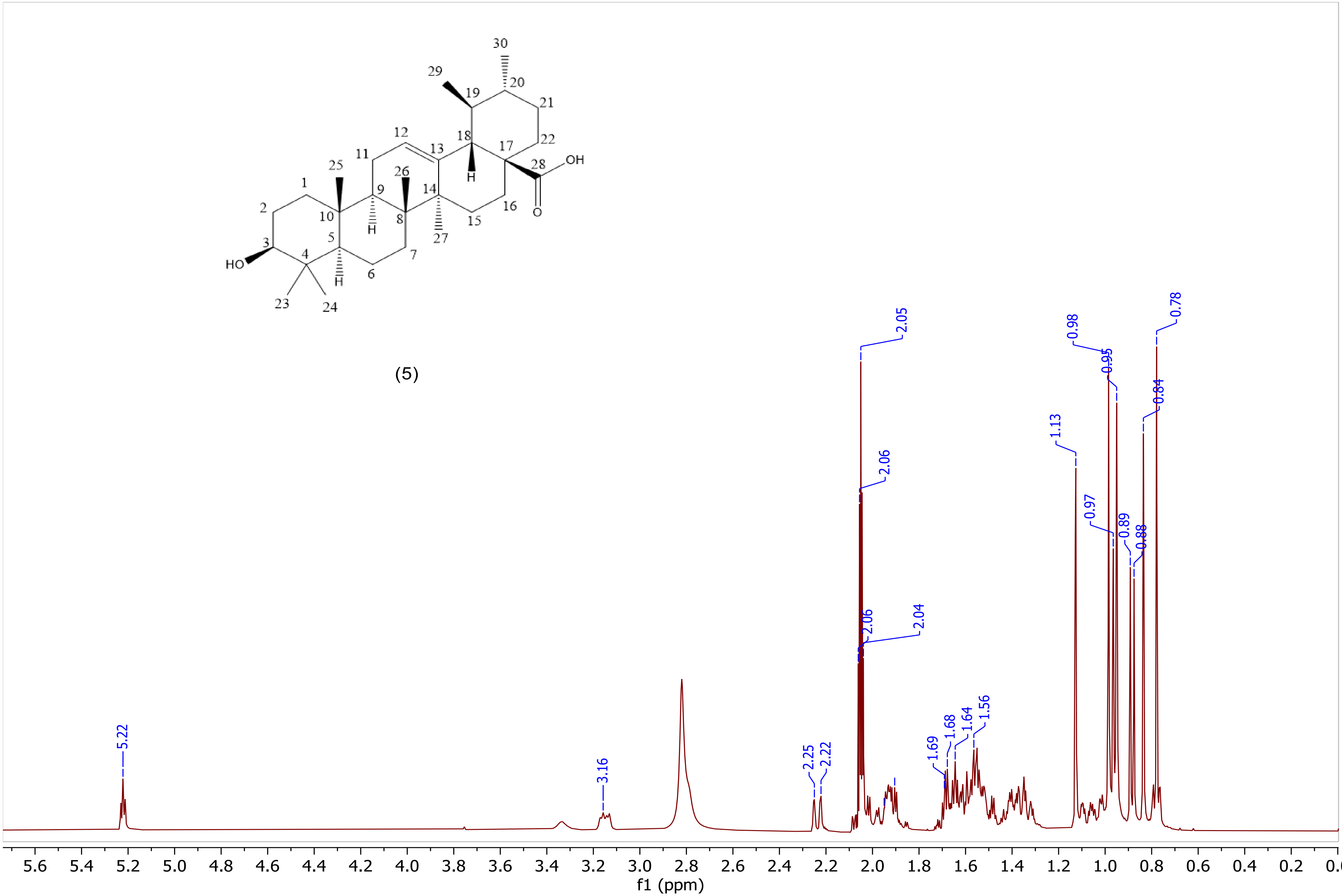

Figure S7. ^{1}H NMR ($CDCl_3$, 600 MHz) of ursolic acid (5)

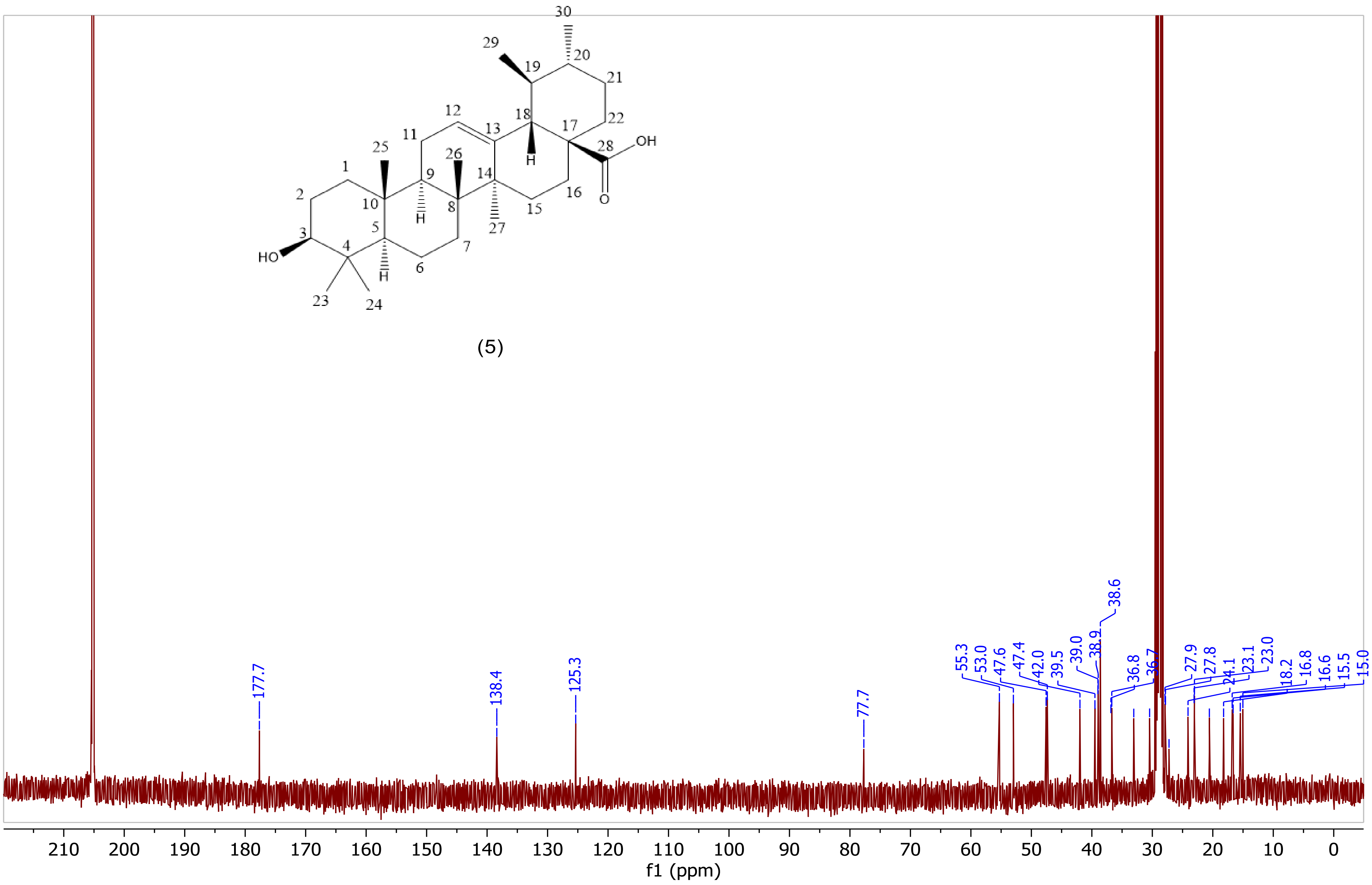

(5)

Figure S8. ^{13}C NMR ($CDCl_3$, 150 MHz) of ursolic acid (5)

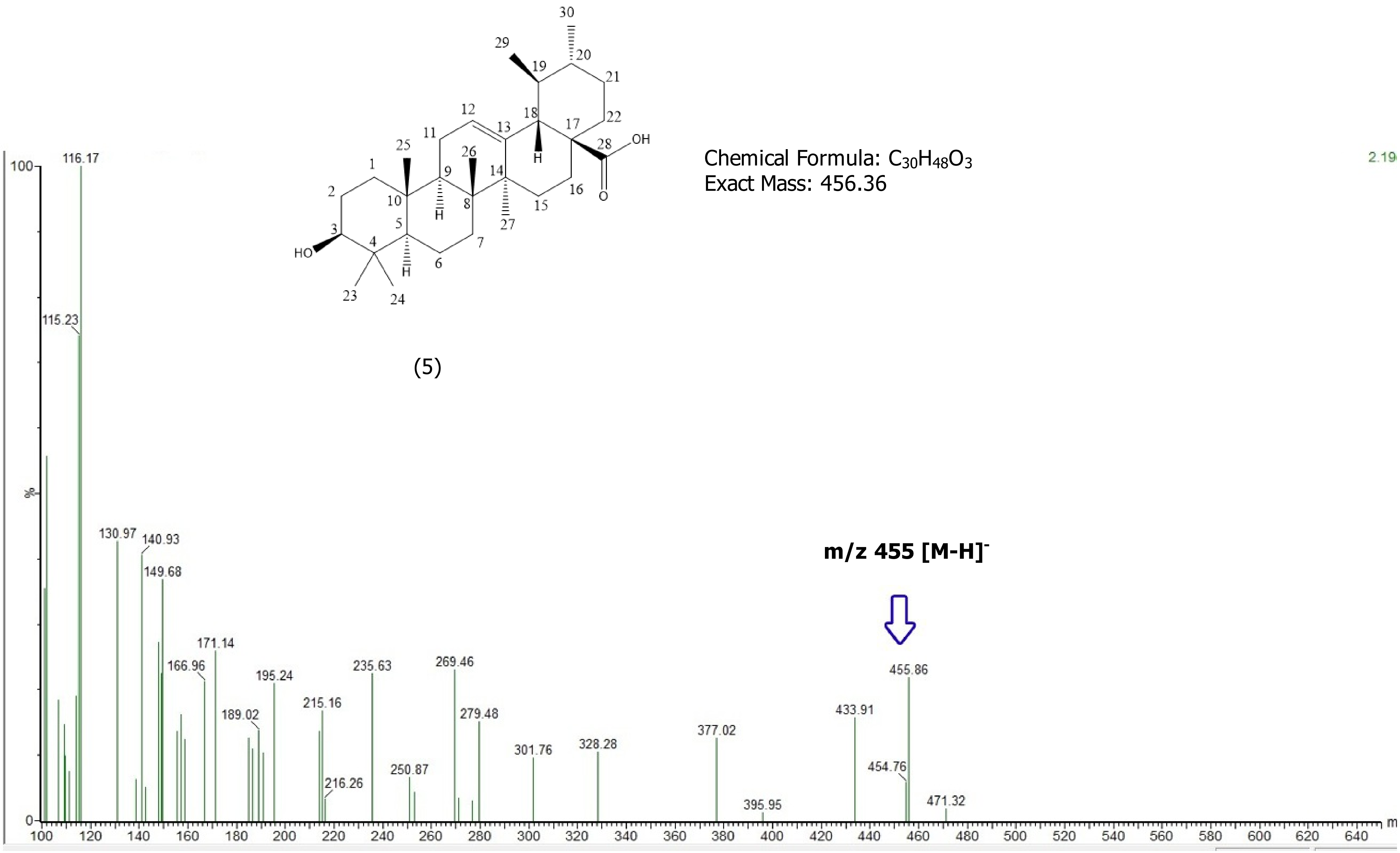

Figure S9. Mass Spectrum of ursolic acid (5)

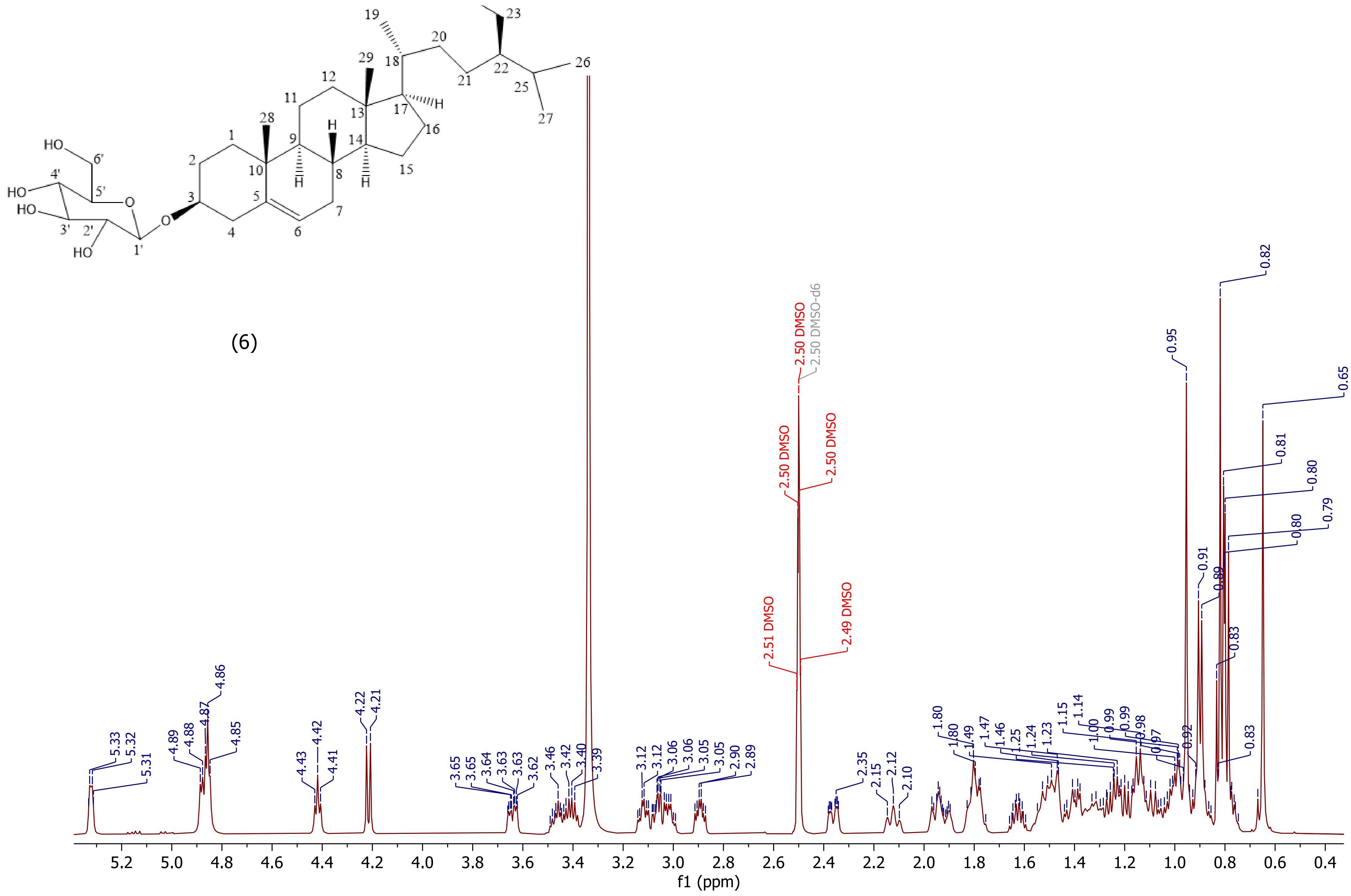

Figure S10. ^{1}H NMR (DMSO-d_6, 600 MHz) of β-sitosterol glucoside (6)

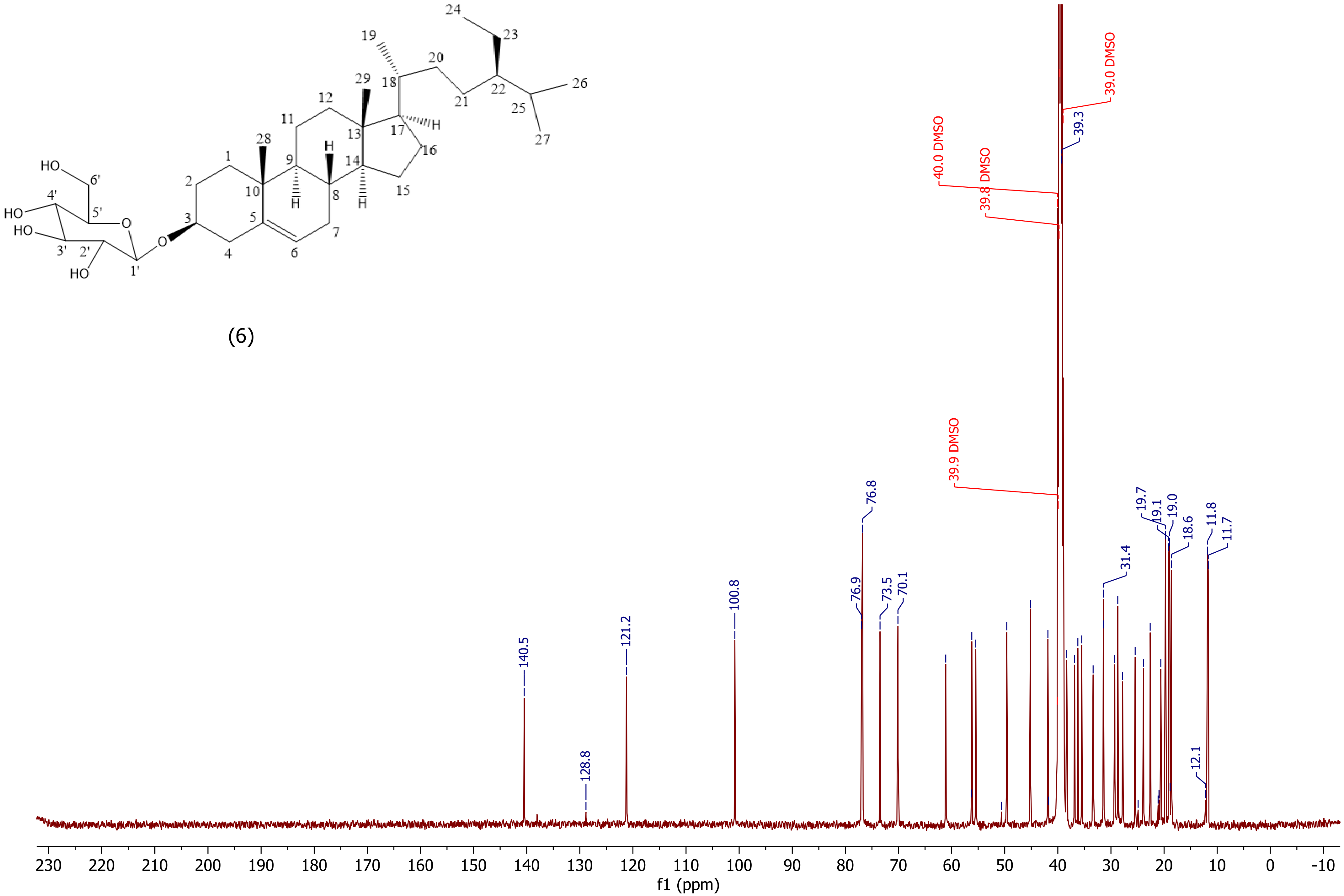

Figure S11. ^{13}C NMR (DMSO-d_6, 150 MHz) of β-sitosterol glucoside (6)

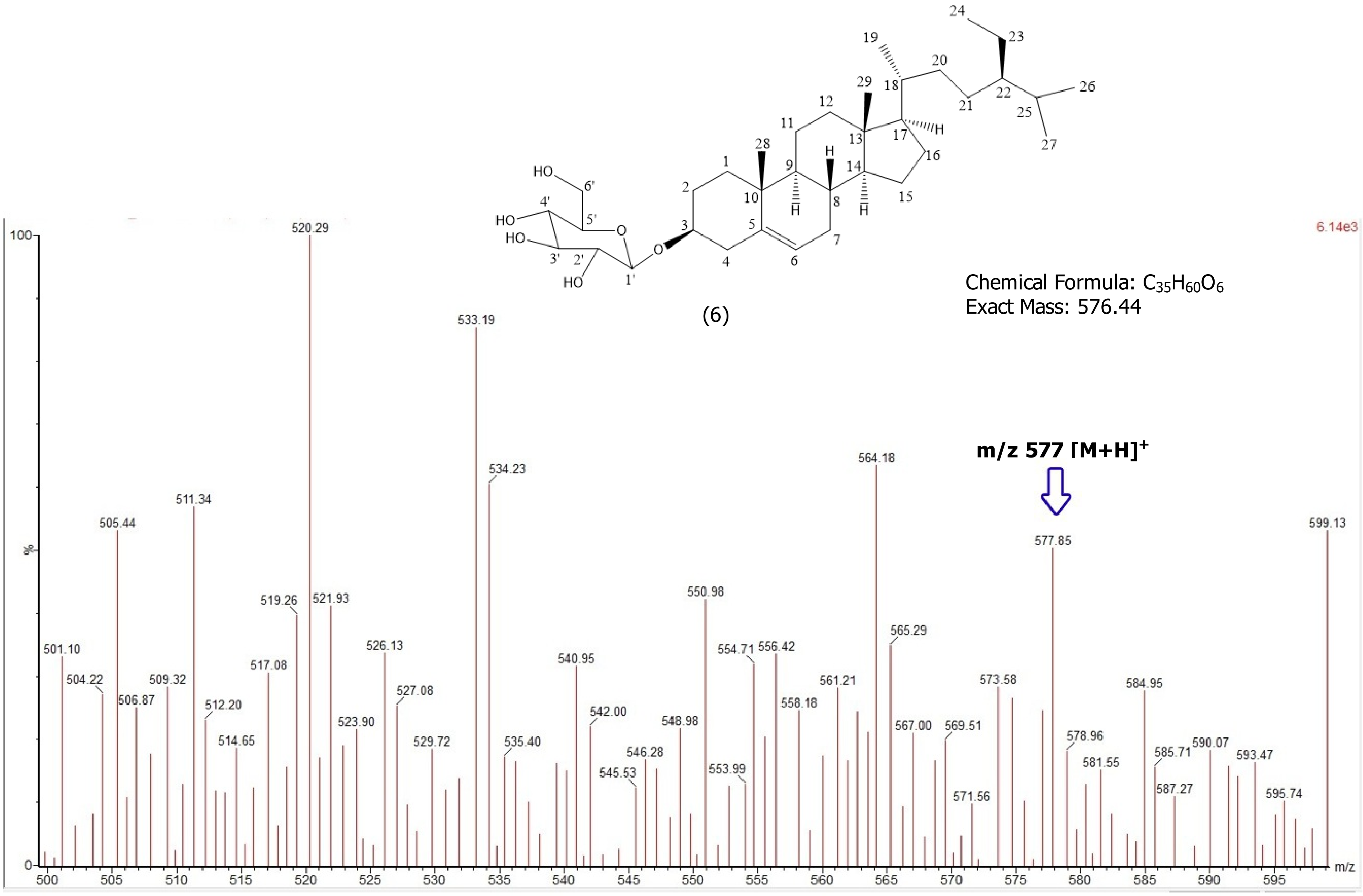

Figure S12. Mass Spectrum of β-sitosterol glucoside (6)

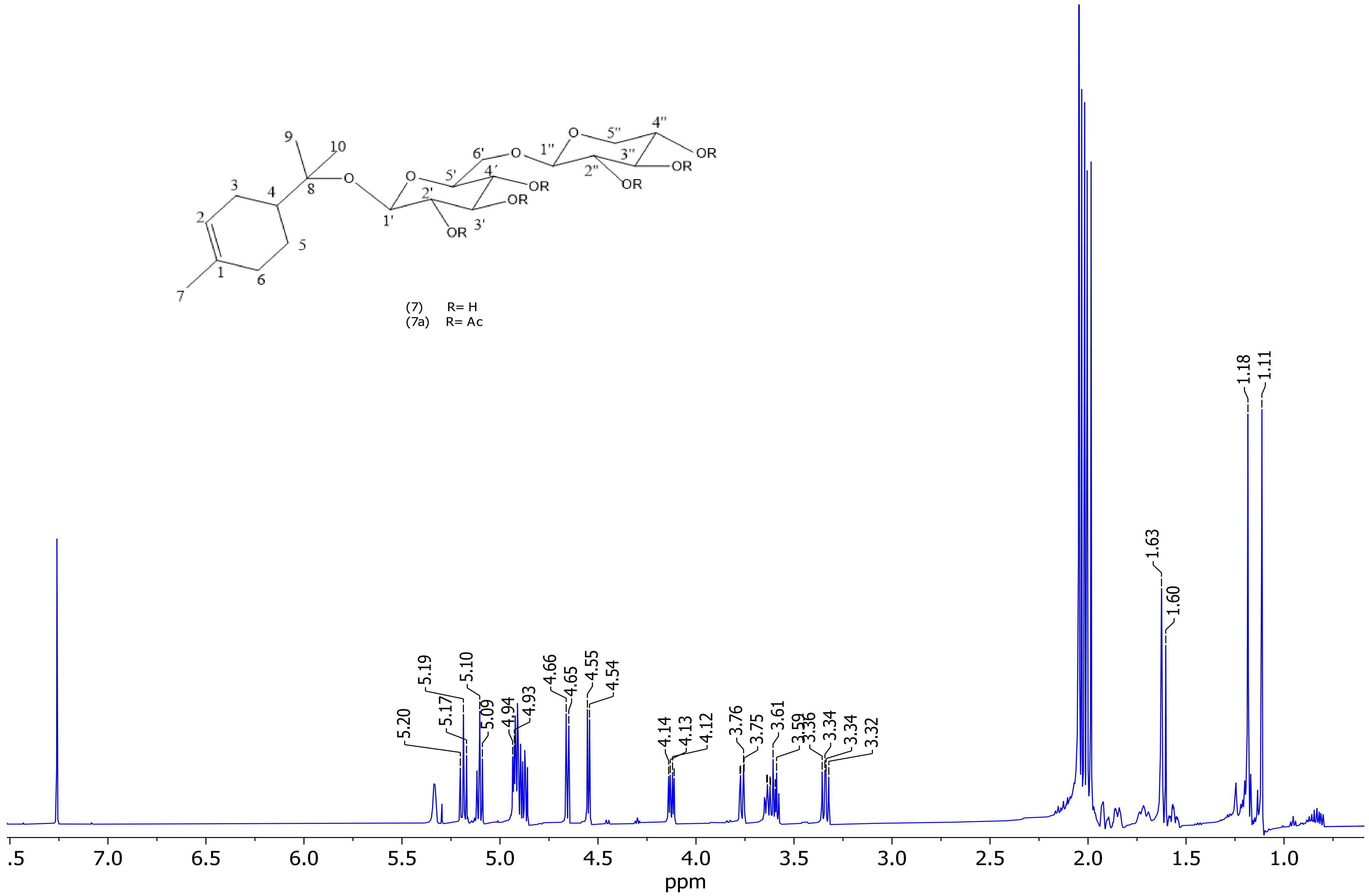

Figure S13. ^{1}H NMR ((CD_3OD, 600 MHz) of 4α-terpineol sambubioside hexaacetate (7a)

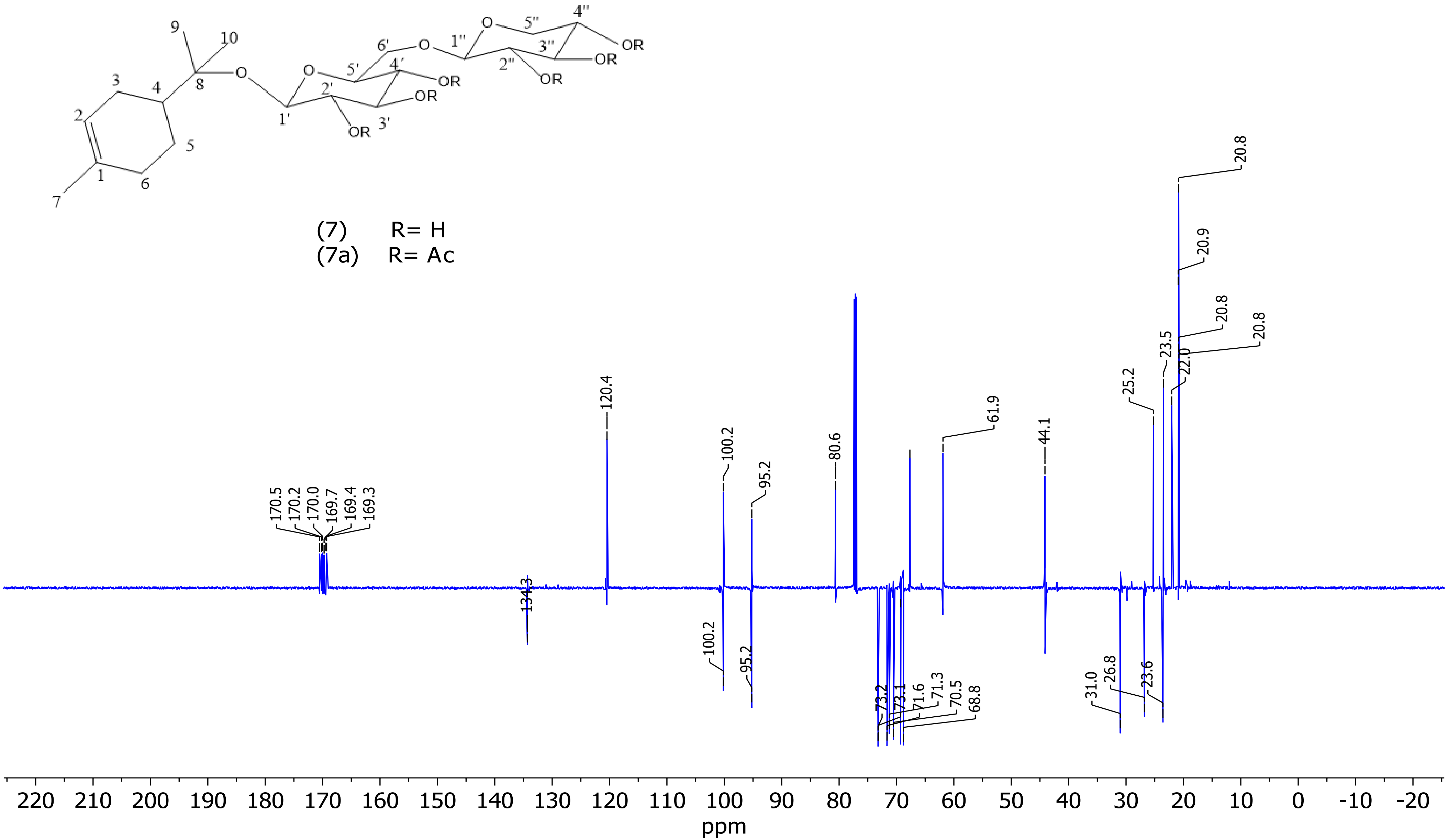

Figure S14. DEPT NMR(CD_3OD, 150 MHz) of 4α-terpineol sambubioside hexaacetate (7a)

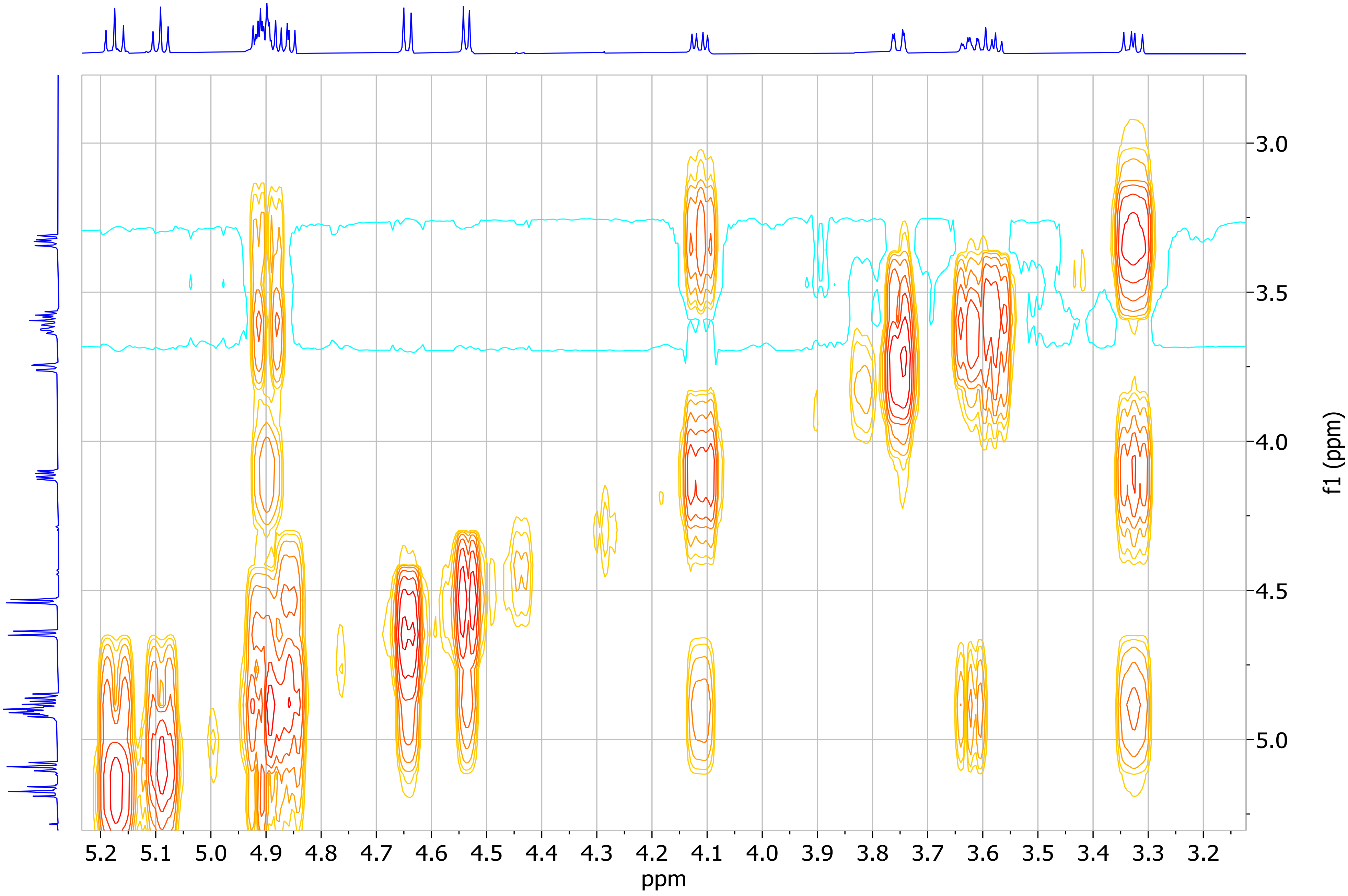

Figure S15. COSY NMR (CD_3OD, 600 MHz) of 4α-terpineol sambubioside hexaacetate (7a)

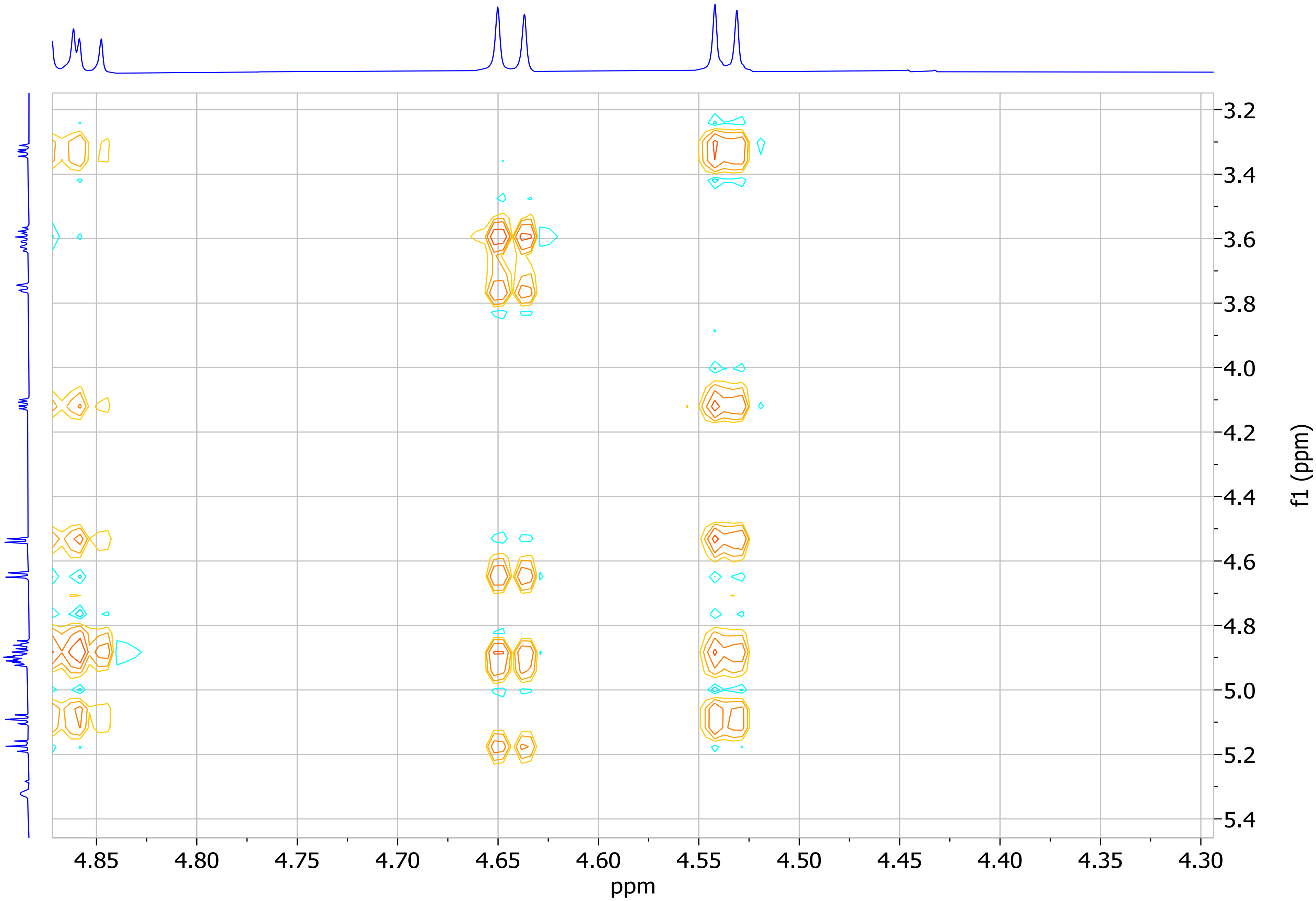

Figure S16. TOCSY NMR (CD_3OD, 600 MHz) of 4α-terpineol sambubioside hexaacetate (7a)

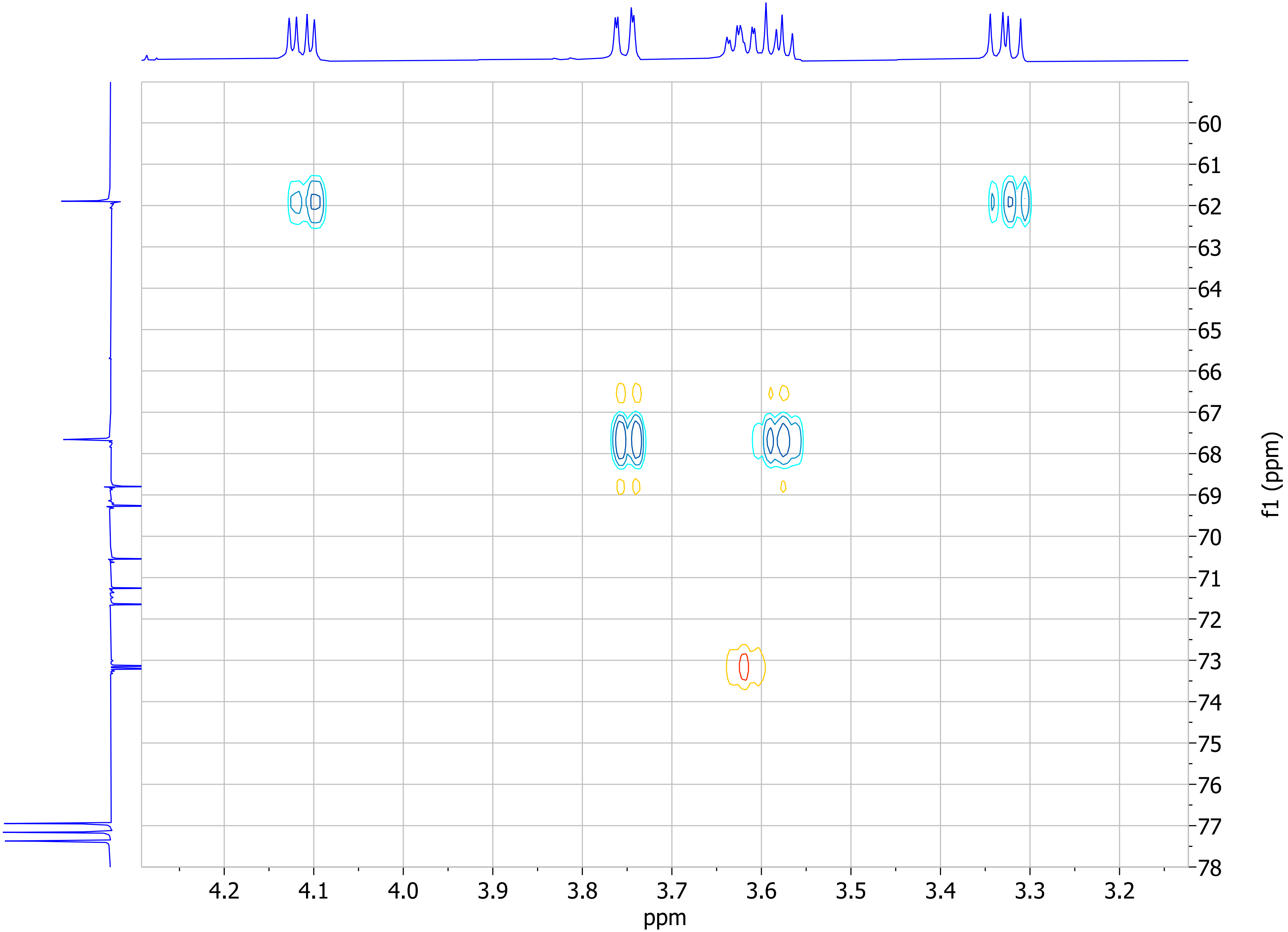

Figure S17. HSQC NMR (CD_3OD, 600 MHz) of he 4α-terpineol sambubioside hexaacetate (7a)

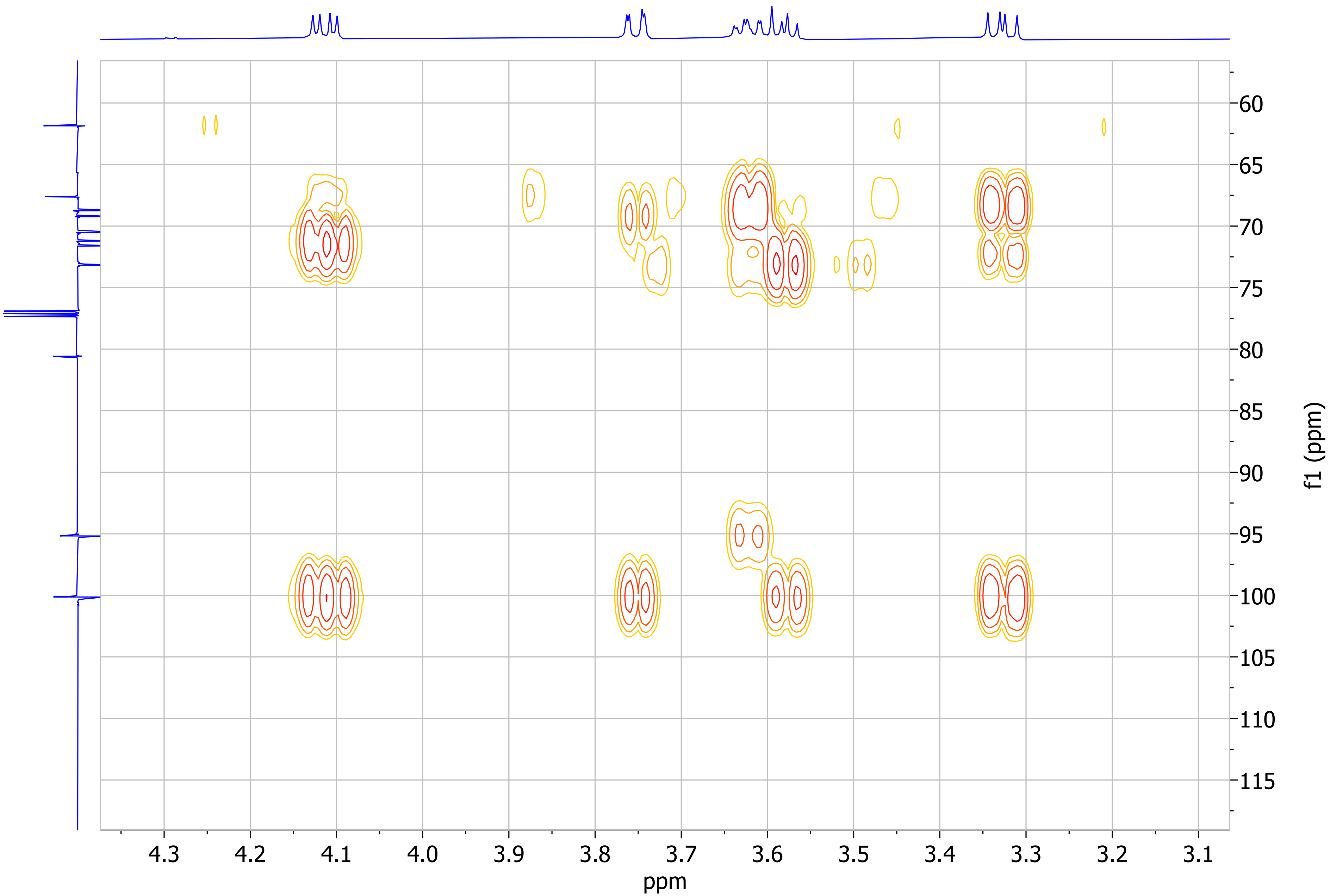

Figure S18. HMBC NMR (CD_3OD, 600 MHz) of 4α-terpineol sambubioside hexaacetate

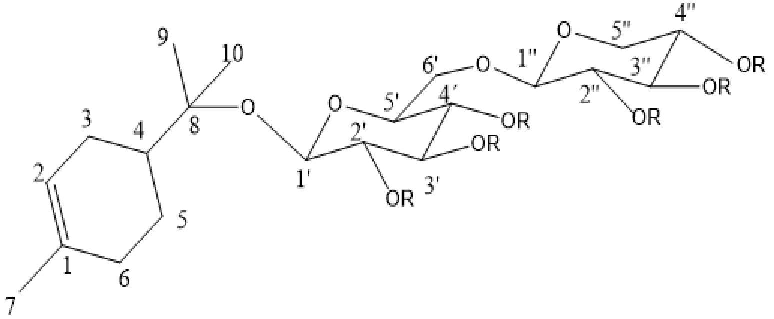

(7) R= H
(7a) R= Ac

(7a)
Chemical Formula: $C_{33}H_{48}O_{16}$
Exact Mass: 700.29

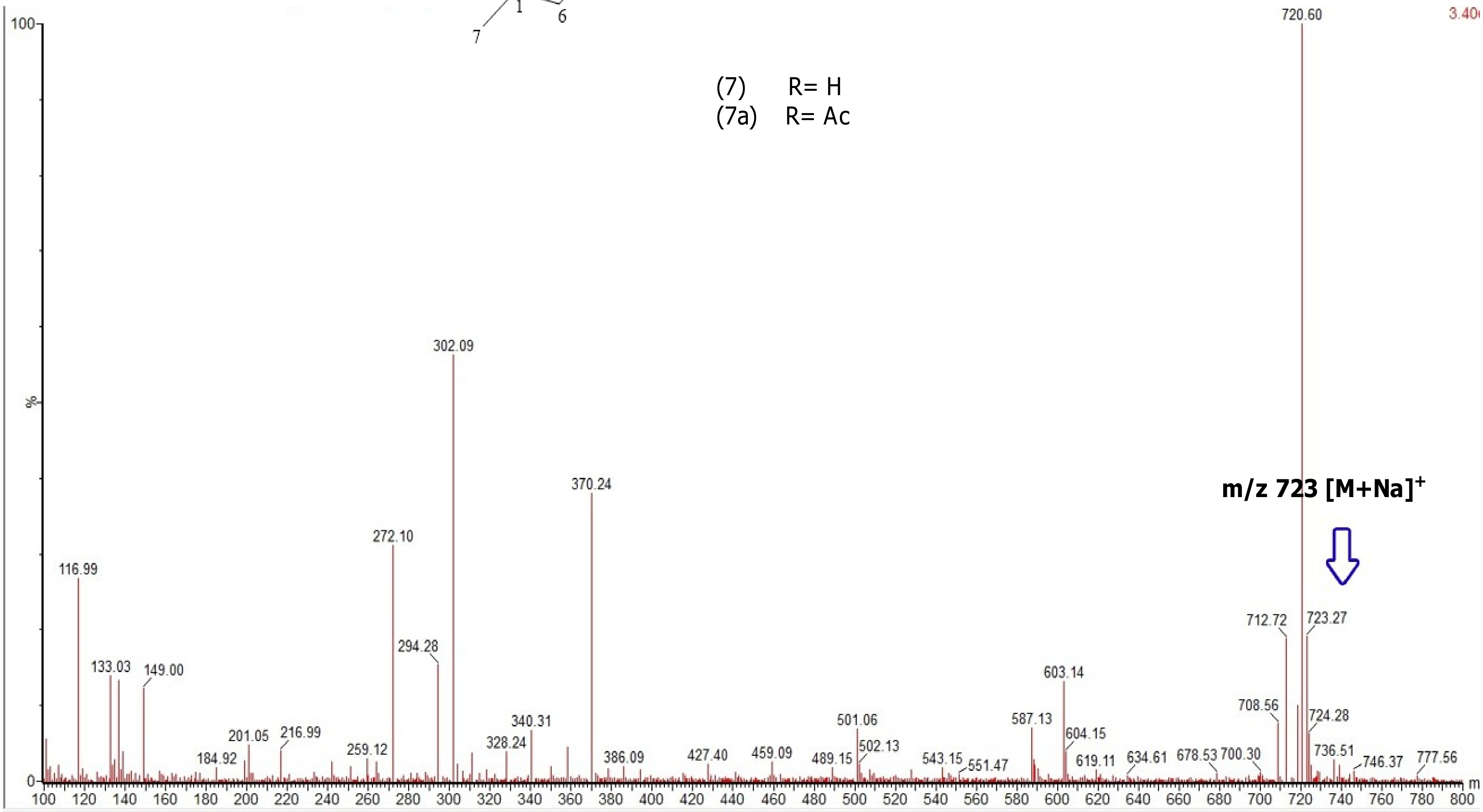

Figure S19. Mass Spectrum of 4α- terpineol sambubioside hexaacetate (7a)

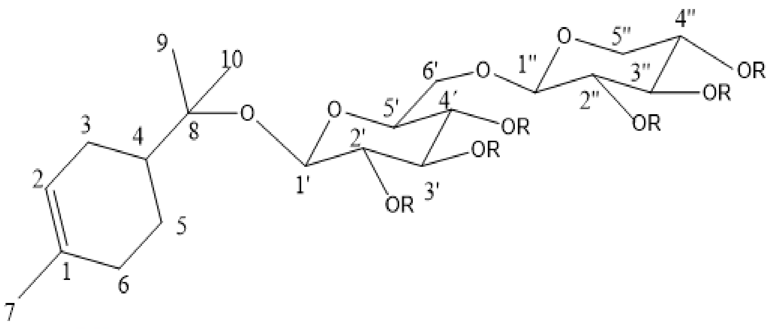

(7a)
Chemical Formula: $C_{33}H_{48}O_{16}$
Exact Mass: 700.29

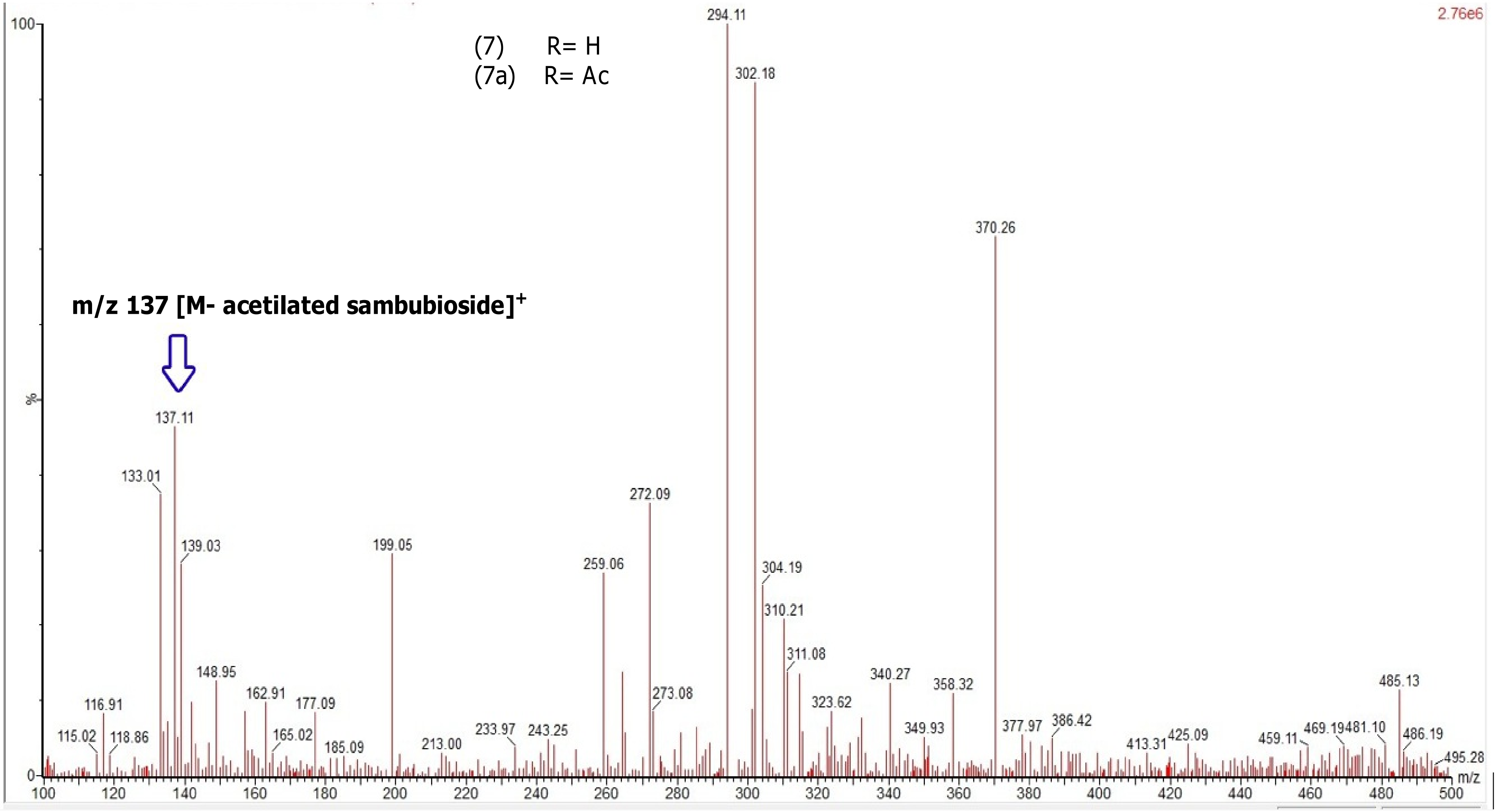

Figure S20. Mass Spectrum of 4α- terpineol sambubioside hexaacetate (7a)

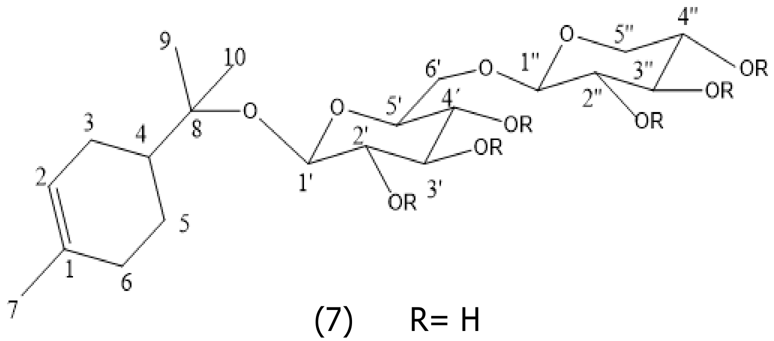

(7) R= H
(7a) R= Ac

(7a)
Chemical Formula: $C_{33}H_{48}O_{16}$
Exact Mass: 700.29

m/z 723 [M+Na]$^+$

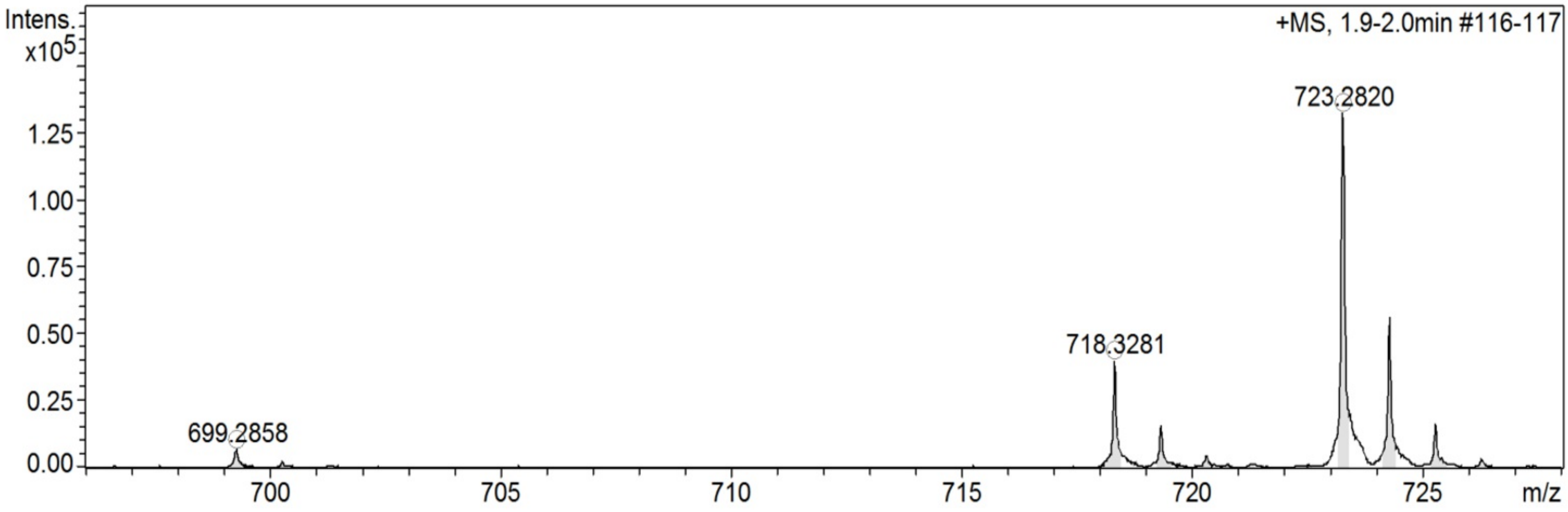

Meas. m/z	#	Ion Formula	Sum Formula	m/z	err [ppm]	|err| [mDa]	mSigma	# mSigma	elem. comp.
699.2858	1	C33H47O16	C33H47O16	699.2859	0.1	0.0	52.2	1	C 56.64 % H 6.77 % O 36.58 %
718.3281	1	C33H52NO16	C33H48O16	718.3281	-0.1	0.0	17.5	1	C 55.14 % H 7.29 % N 1.95 % O 35.62 %
723.2820	1	C33H48NaO16		723.2835	2.0	1.5	31.4	1	C 54.77 % H 6.69 % Na 3.18 % O 35.37 %

Figure S21. HR-ESI-MS of 4α- terpineol sambubioside hexaacetate (7a)

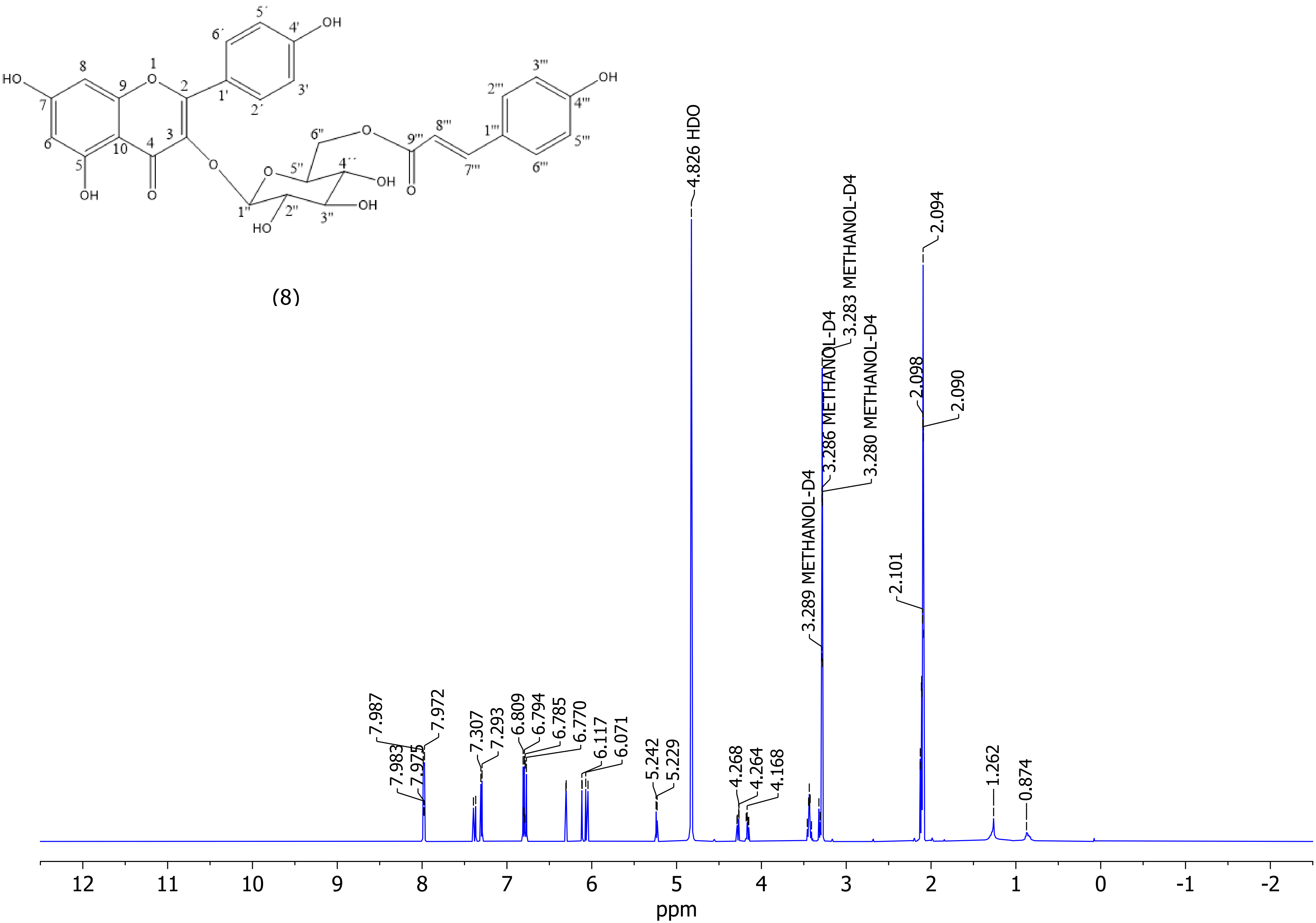

Figure S22. ^{1}H NMR (MeOD, 600 MHz) of tiliroside (8)

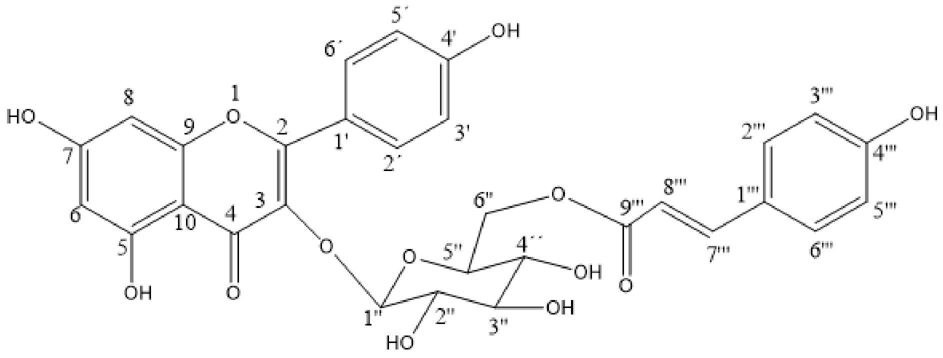

(8)

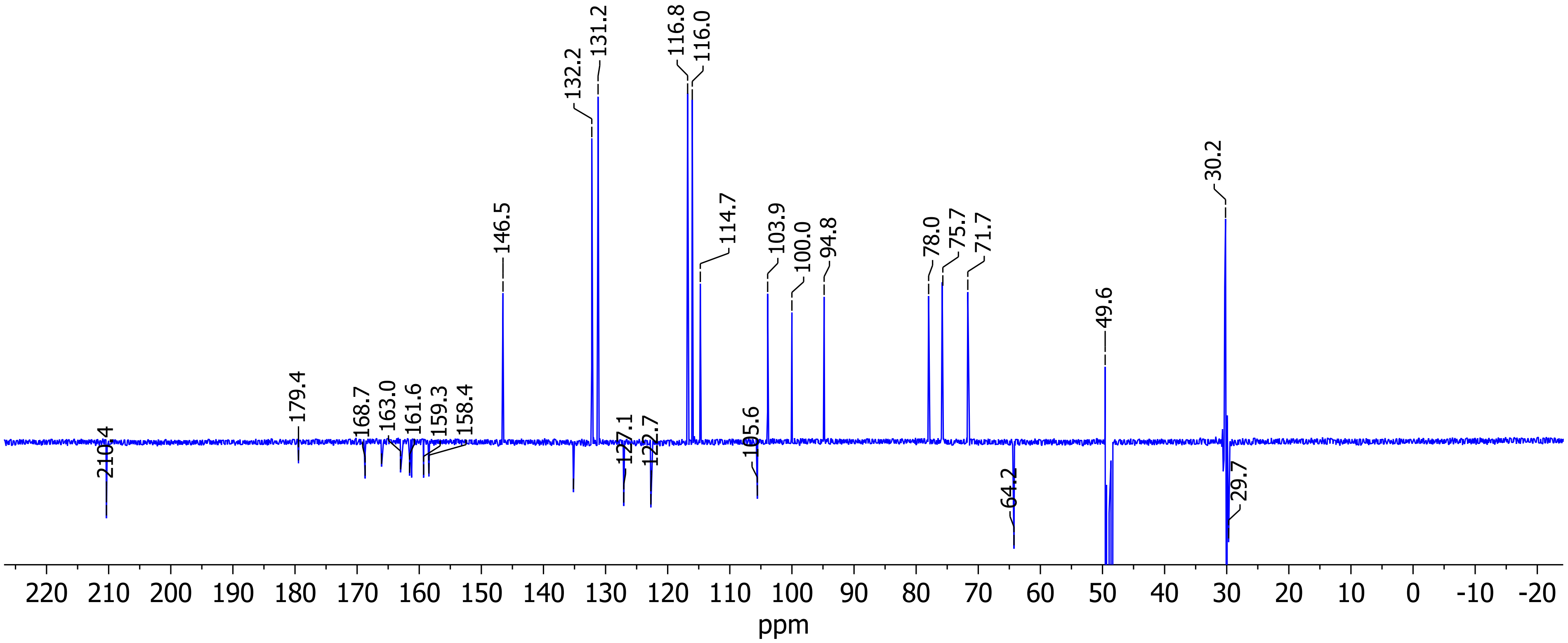

Figure S23. DEPT NMR (MeOD, 150 MHz) of tiliroside (8)

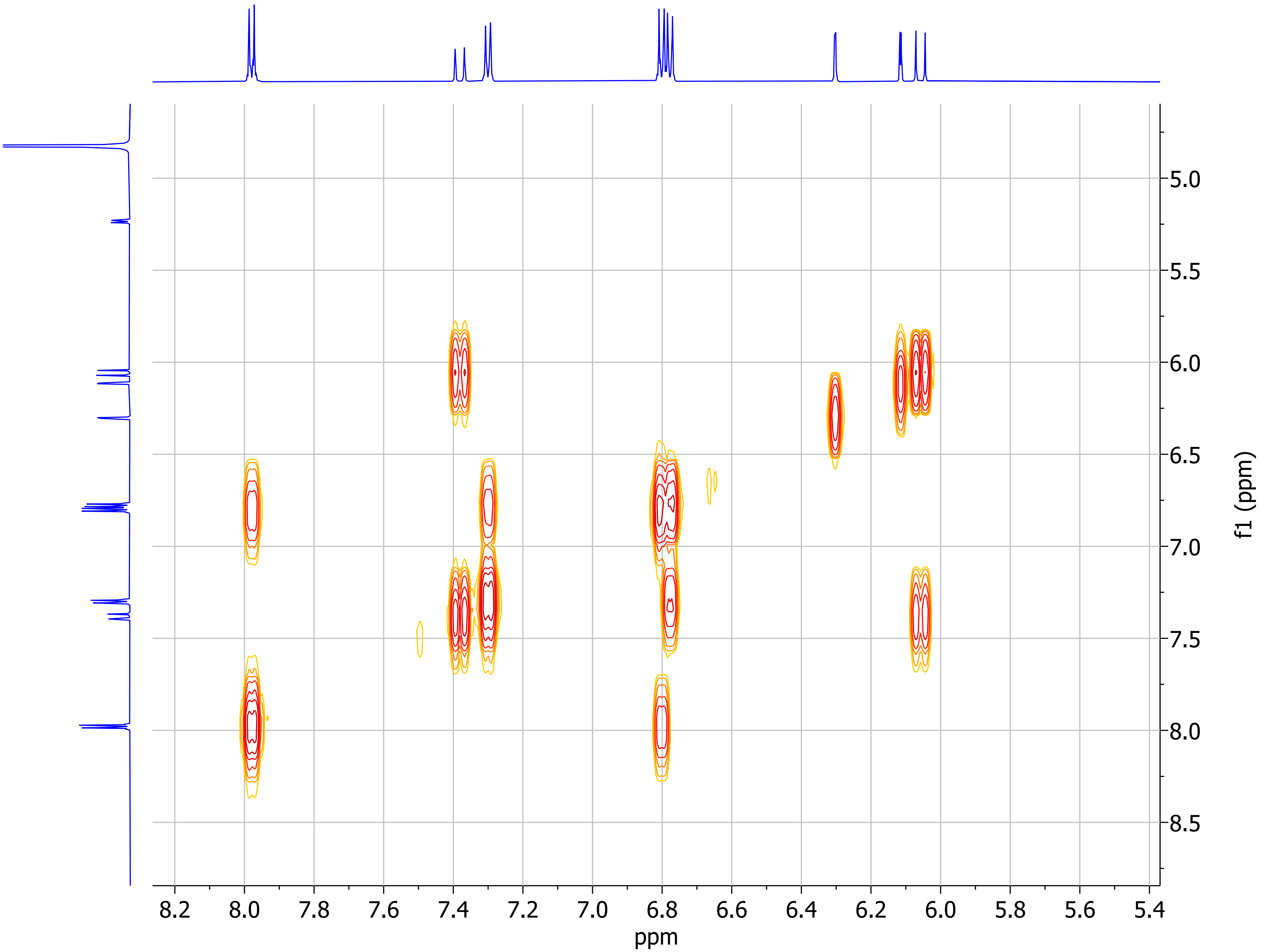

Figure S24. COSY NMR (MeOD, 600 MHz) of tiliroside (8)

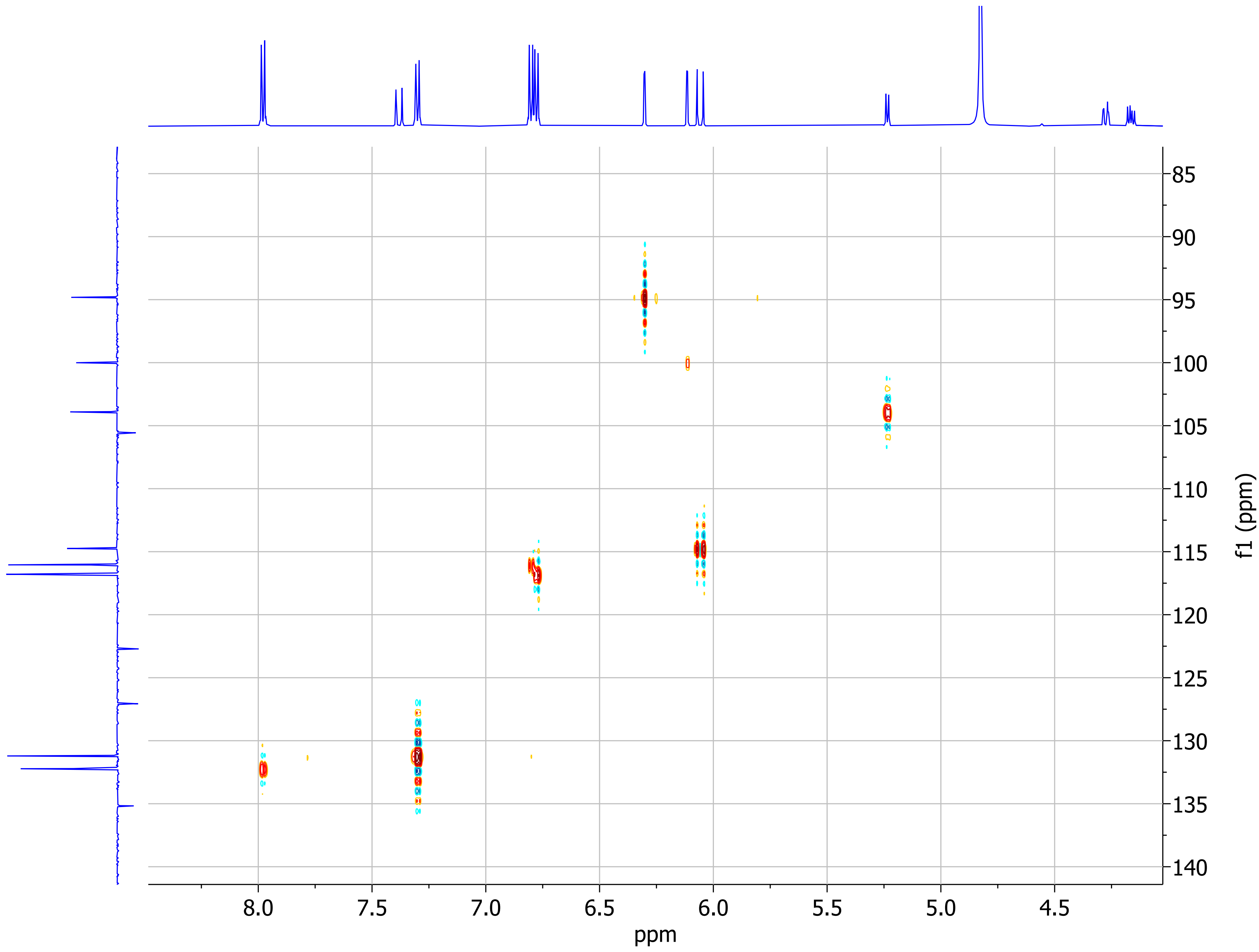

Figure S25. HSQC NMR (MeOD, 600 MHz) of tiliroside (8)

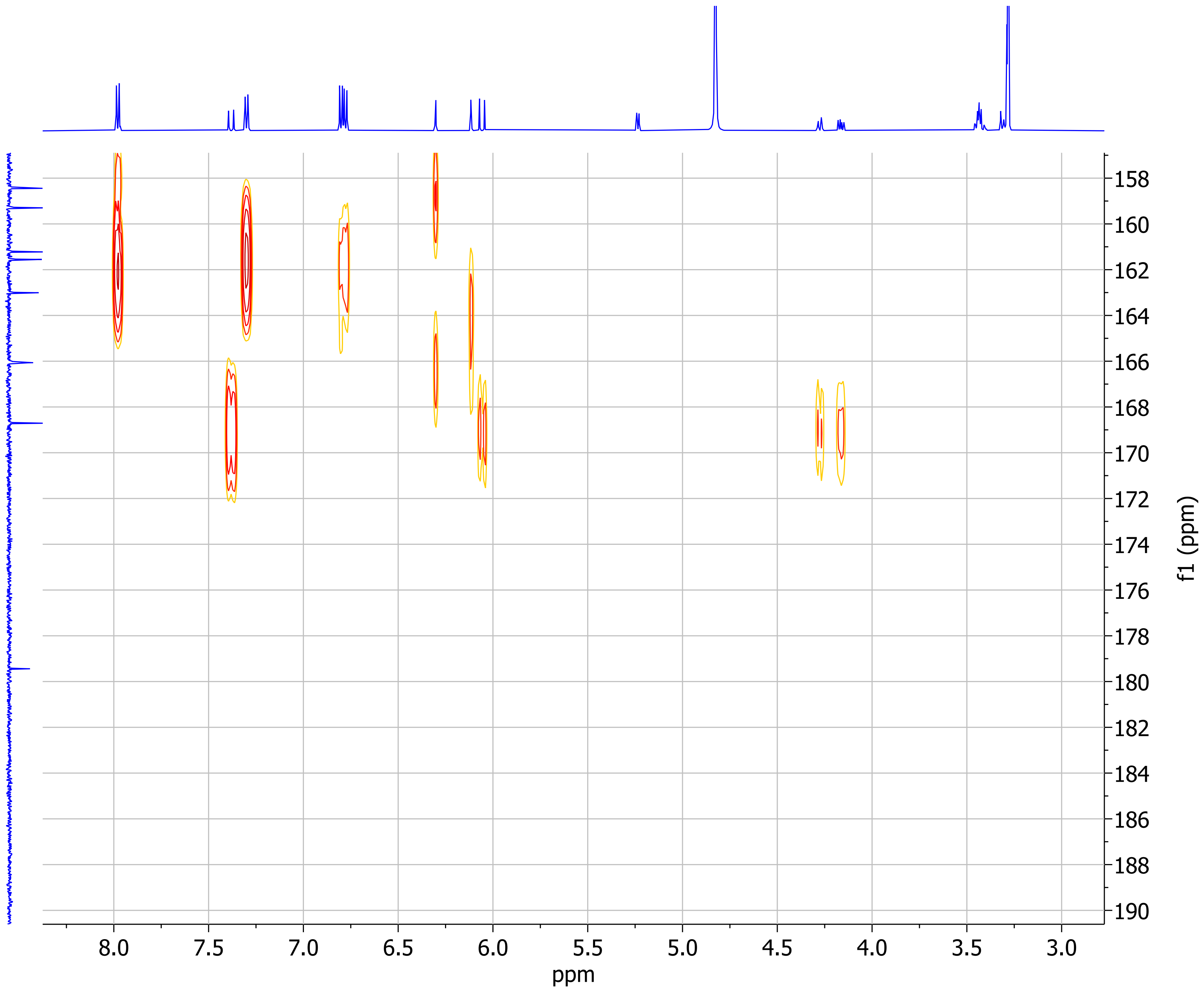

Figure S26. HSQC NMR (MeOD, 600 MHz) of tiliroside (8)

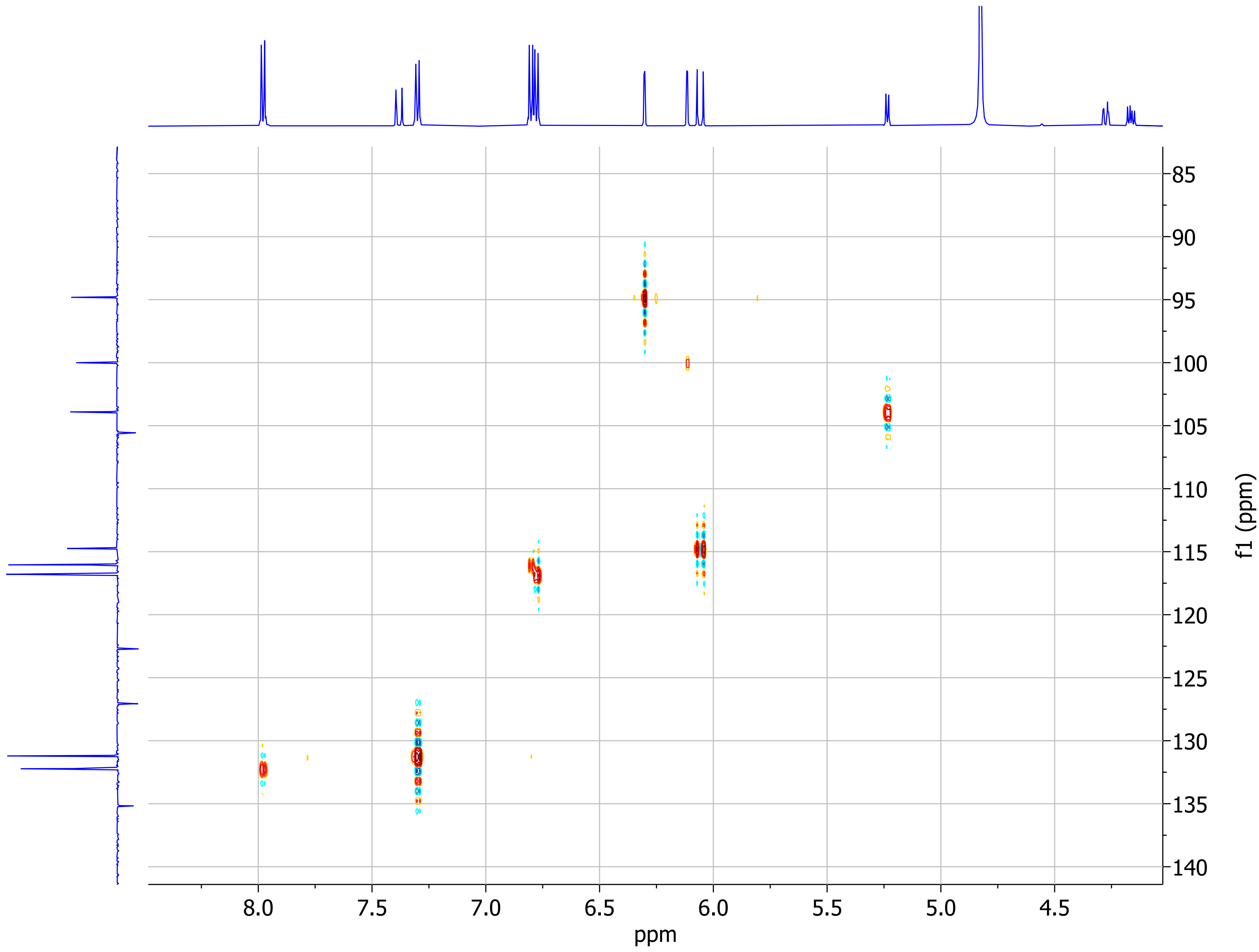

Figure S27. HSQC NMR (MeOD, 600 MHz) of tiliroside (8)

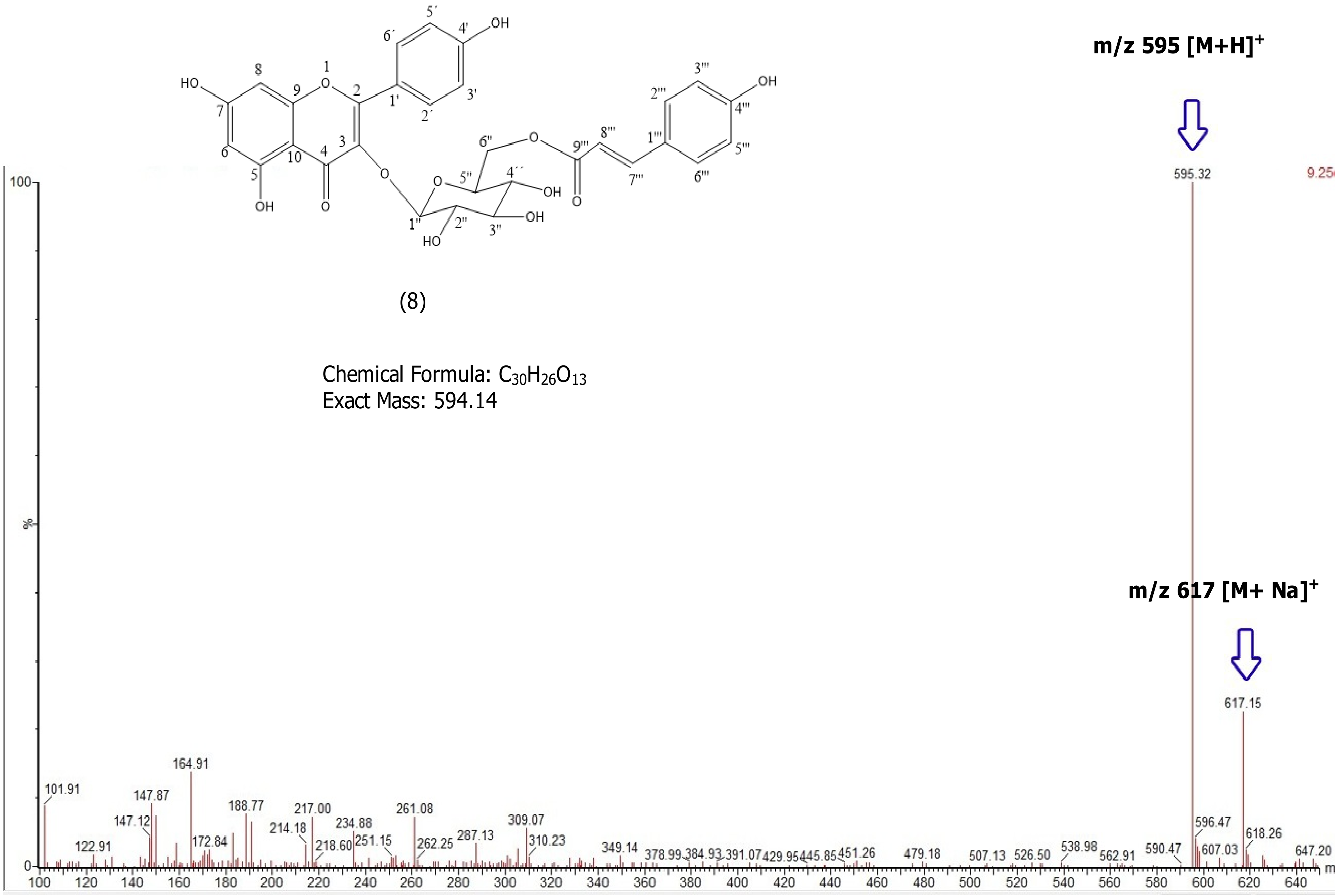

Figure S28. Mass Spectrum of tiliroside (8)

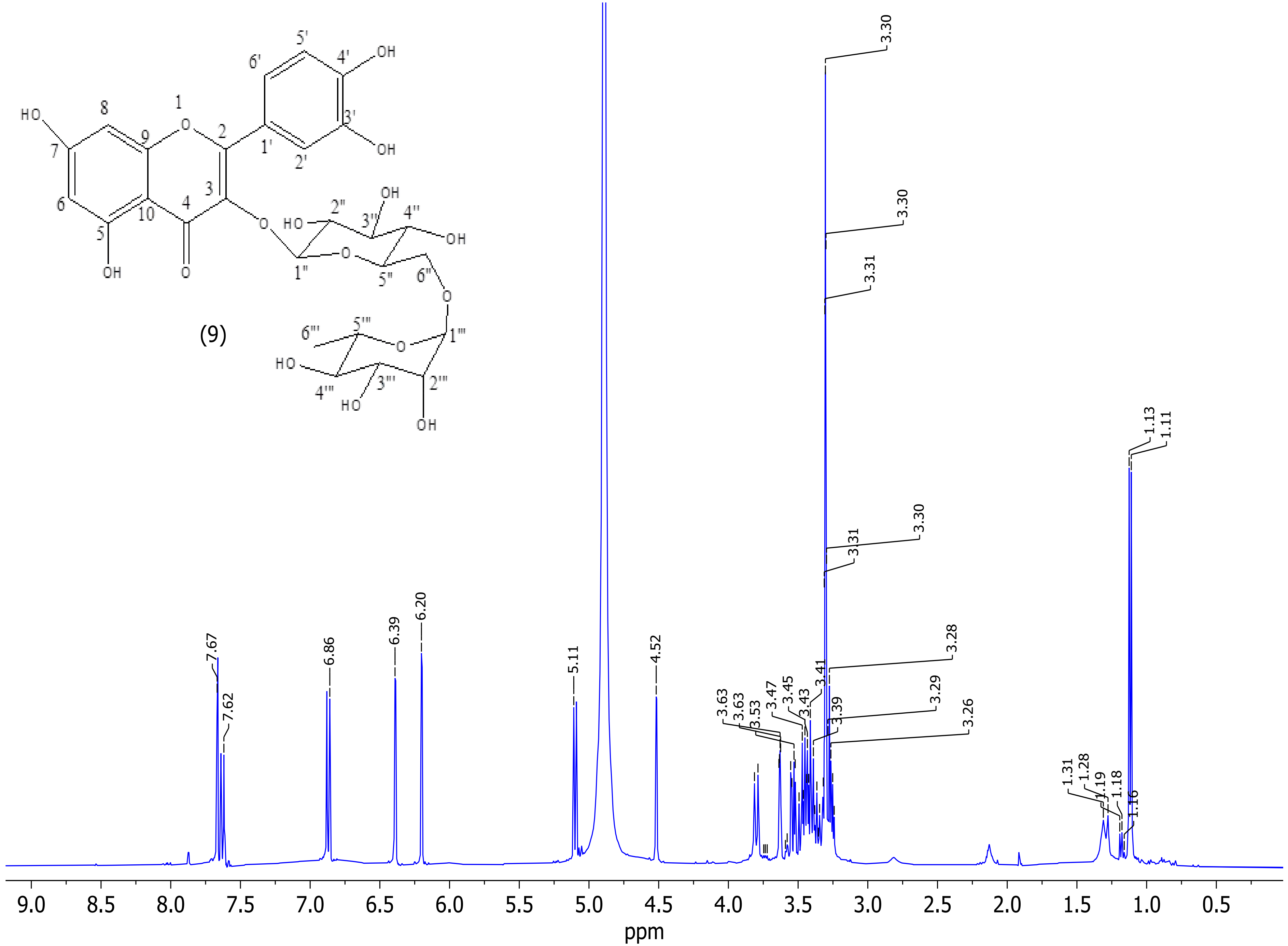

Figure S29. ^{1}H NMR (CD_3OD, 600 MHz) of rutine (9)

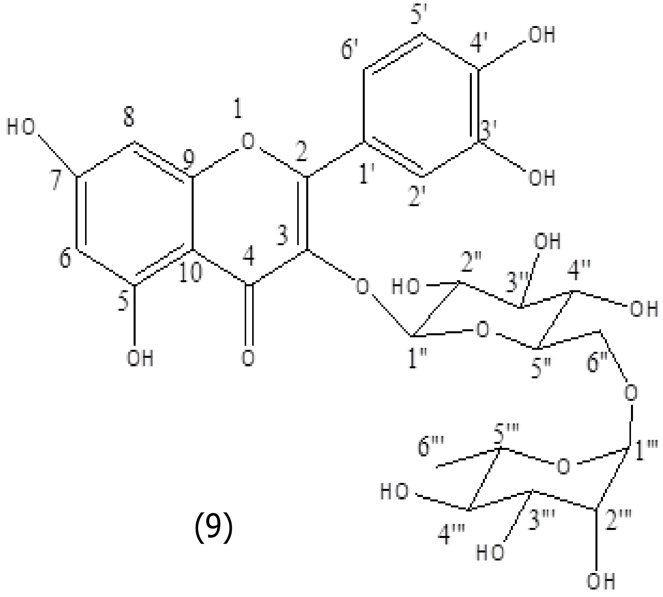

(9)

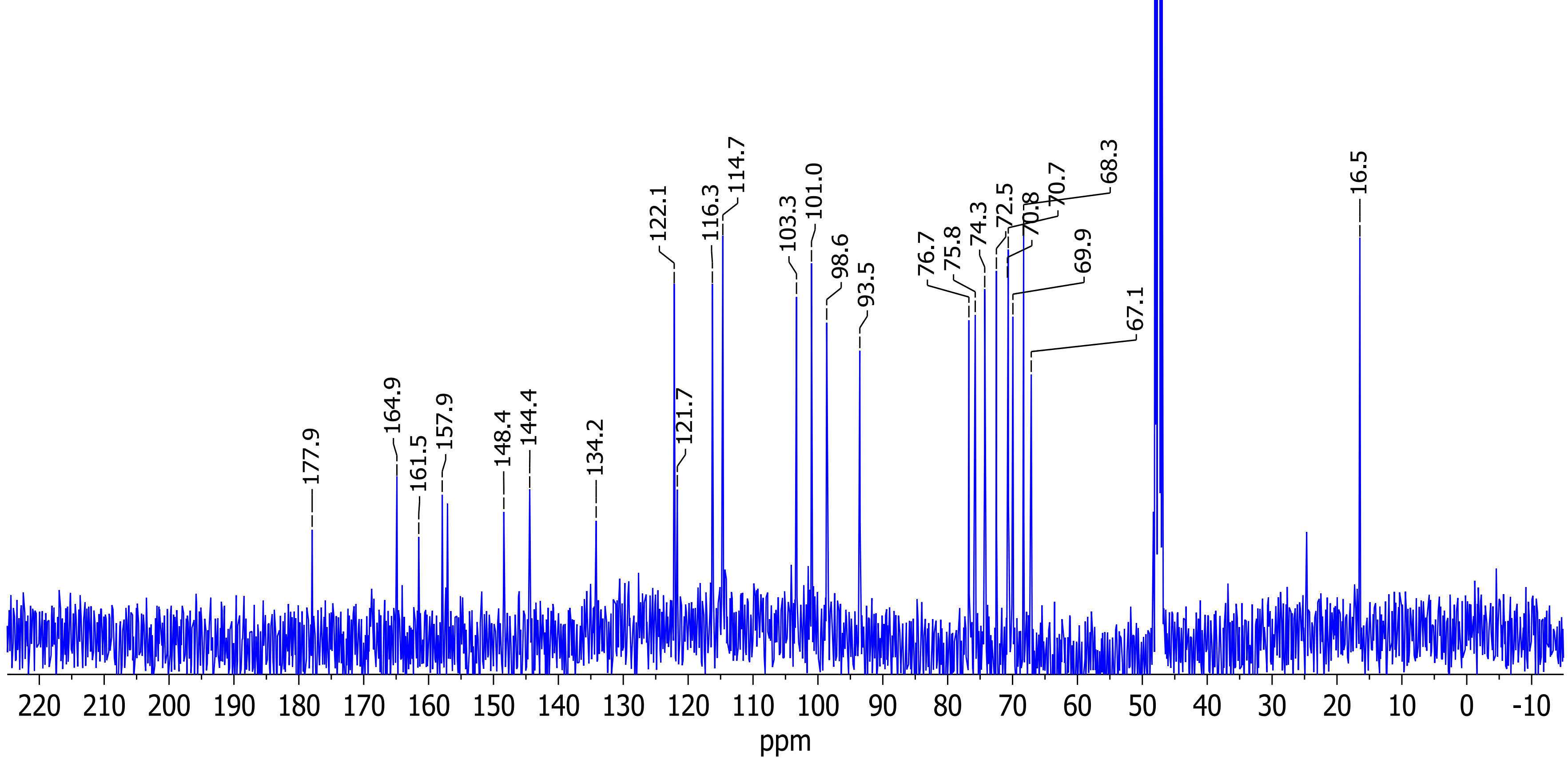

Figure S30. ^{13}C NMR (CD_3OD, 600 MHz) of rutine (9)

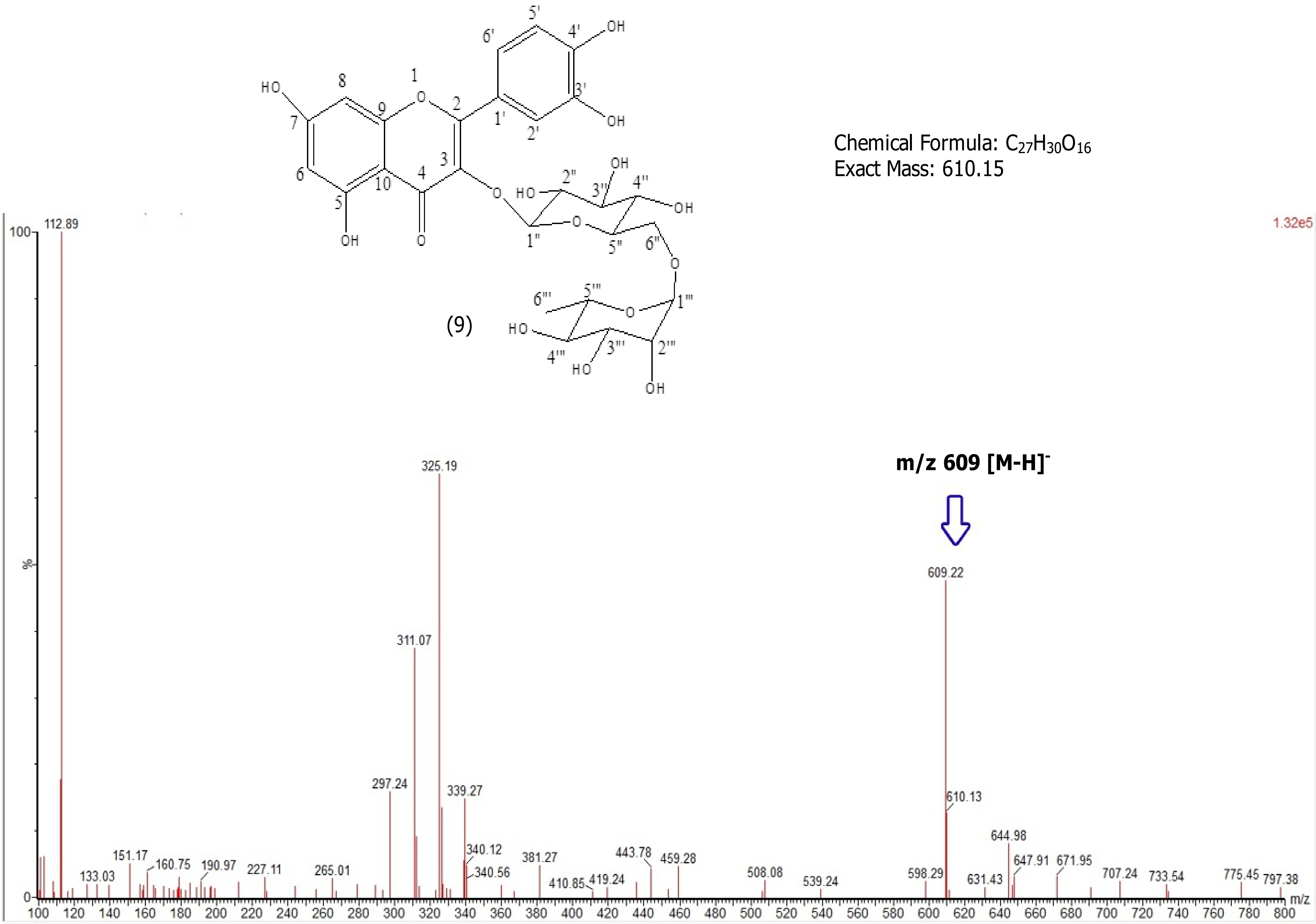

Figure S31. Mass Spectrum of rutine (9)

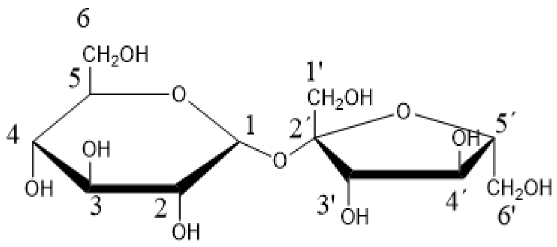

(10)

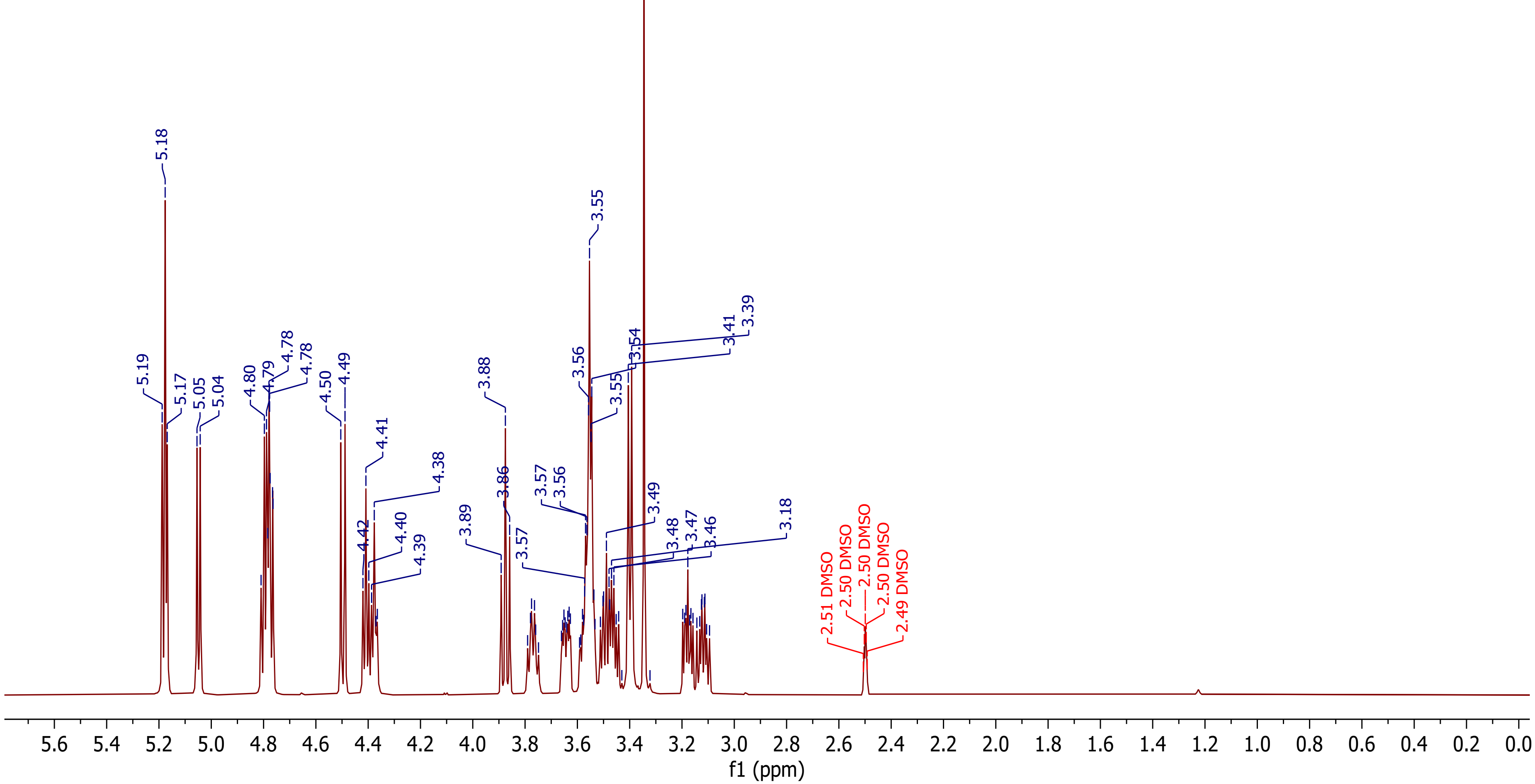

Figure S32. ^{1}H NMR (DMSO-d_6, 600 MHz) of sucrose (10)

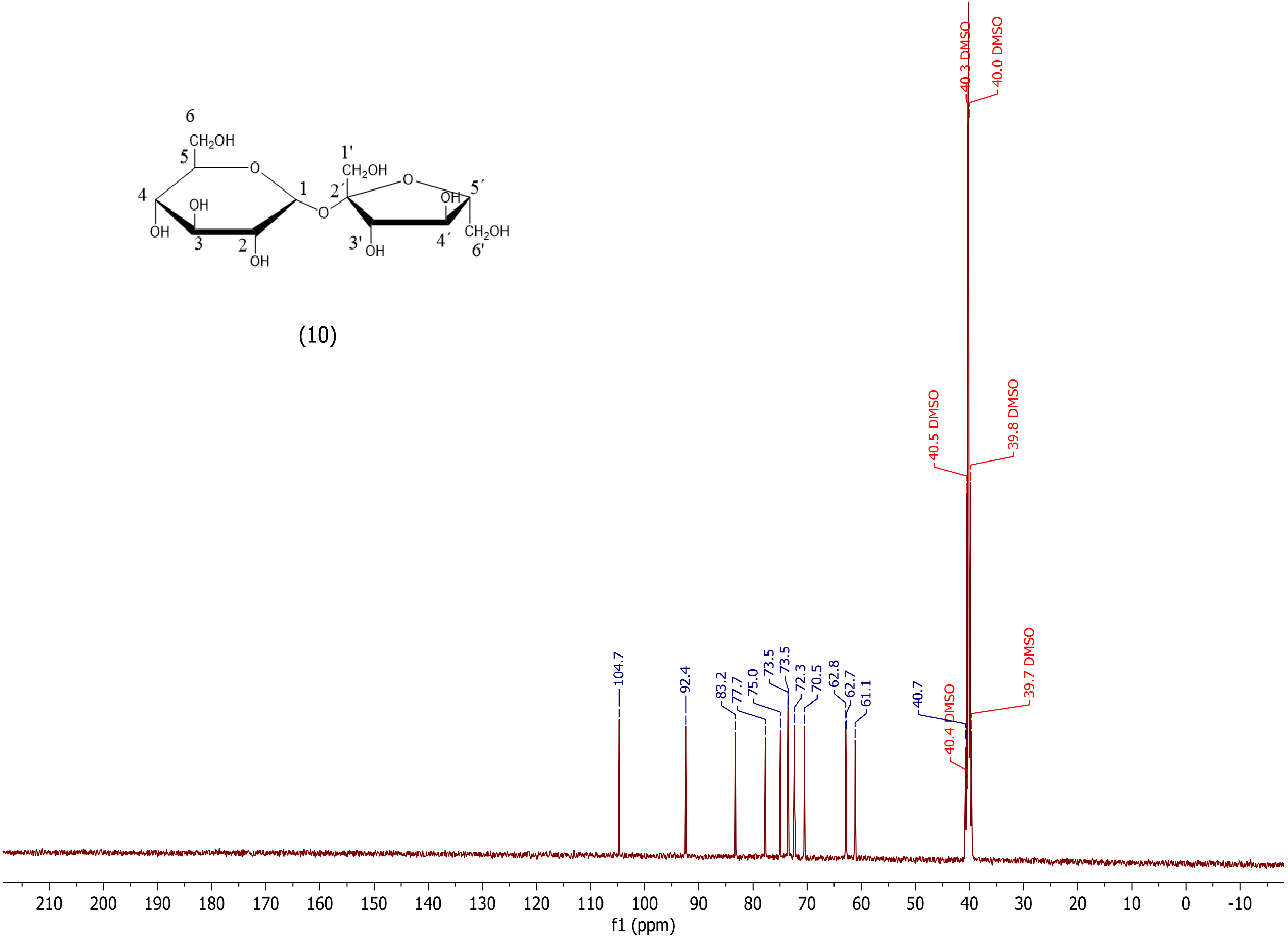

Figure S33. ^{13}C NMR (DMSO-d_6, 600 MHz) of sucrose (10)

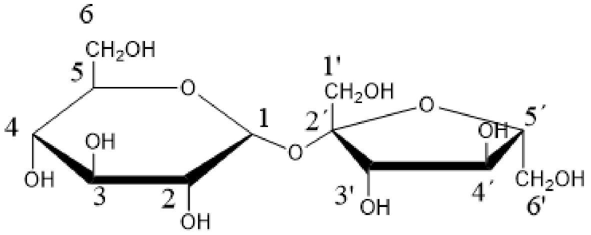

(10)

Chemical Formula: $C_{12}H_{22}O_{11}$
Exact Mass: 342.12

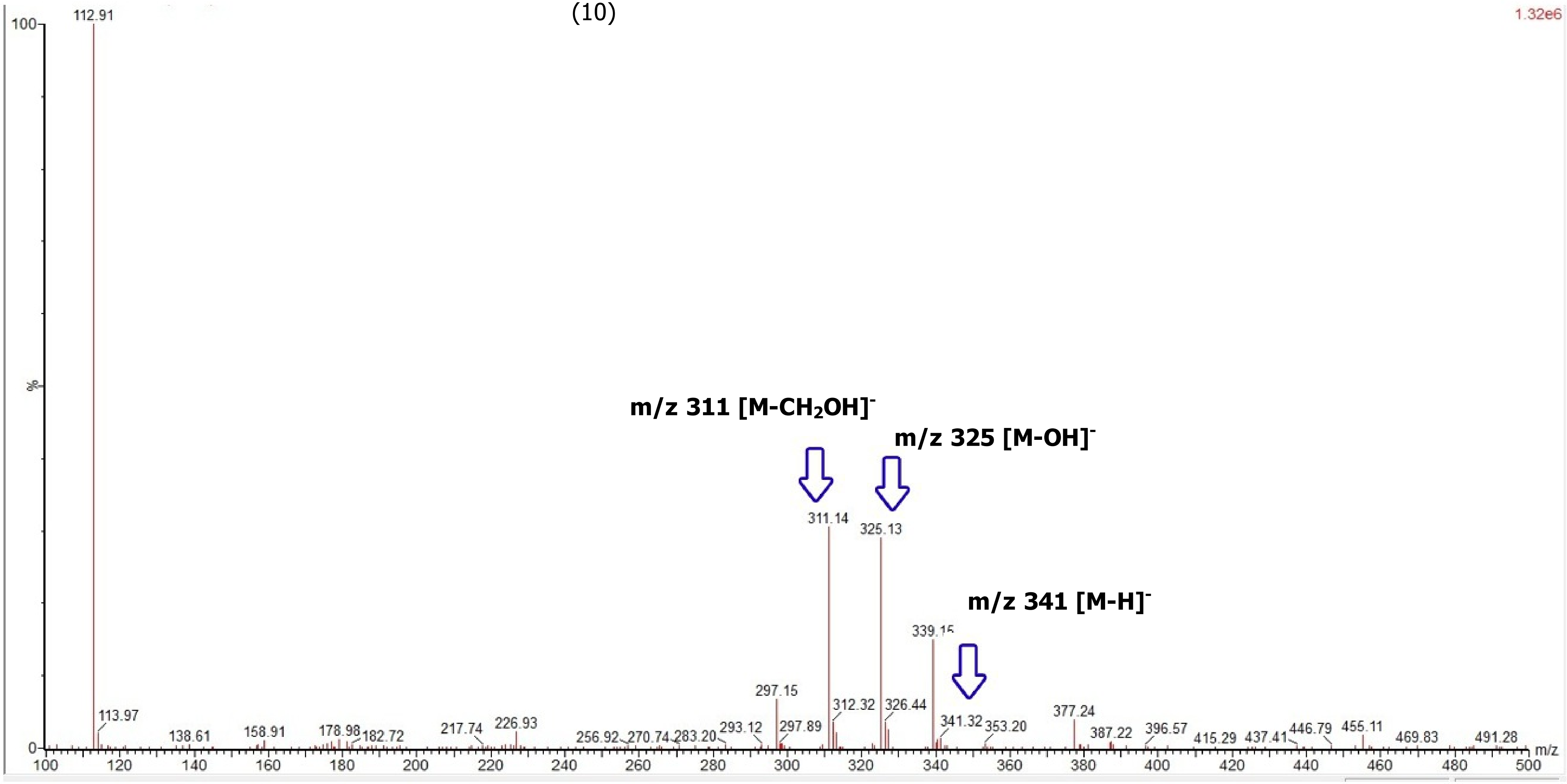

Figure S34. Mass Spectrum of sucrose (10)

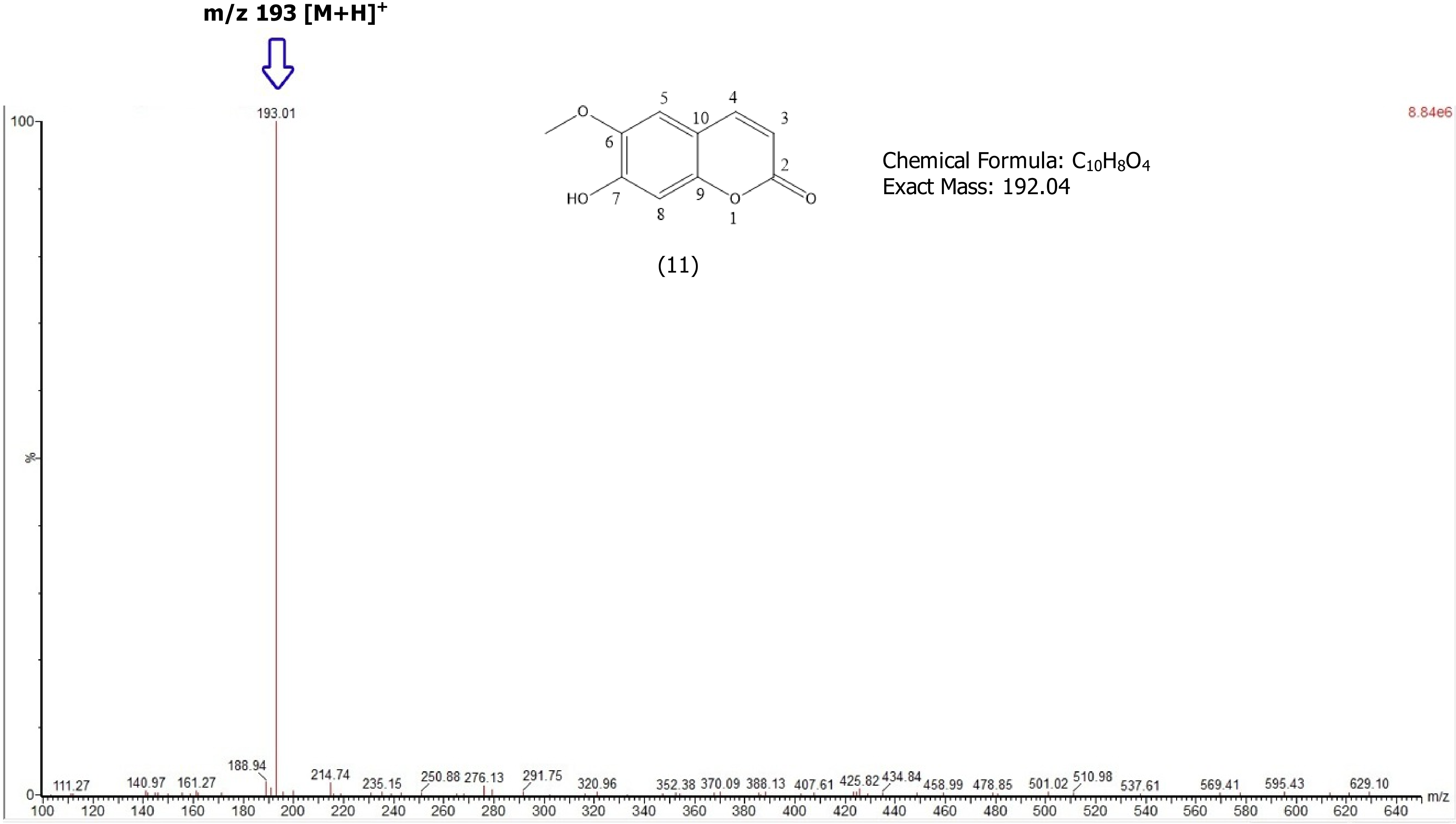

Figure S35. Mass Spectrum of scopoletin (11)

m/z 180 [M]$^+$

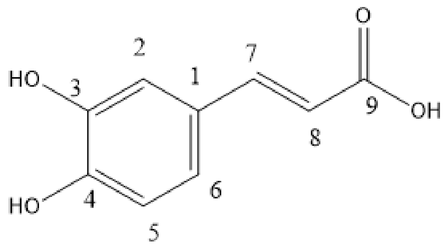

(12)

Chemical Formula: $C_9H_8O_4$
Exact Mass: 180.04

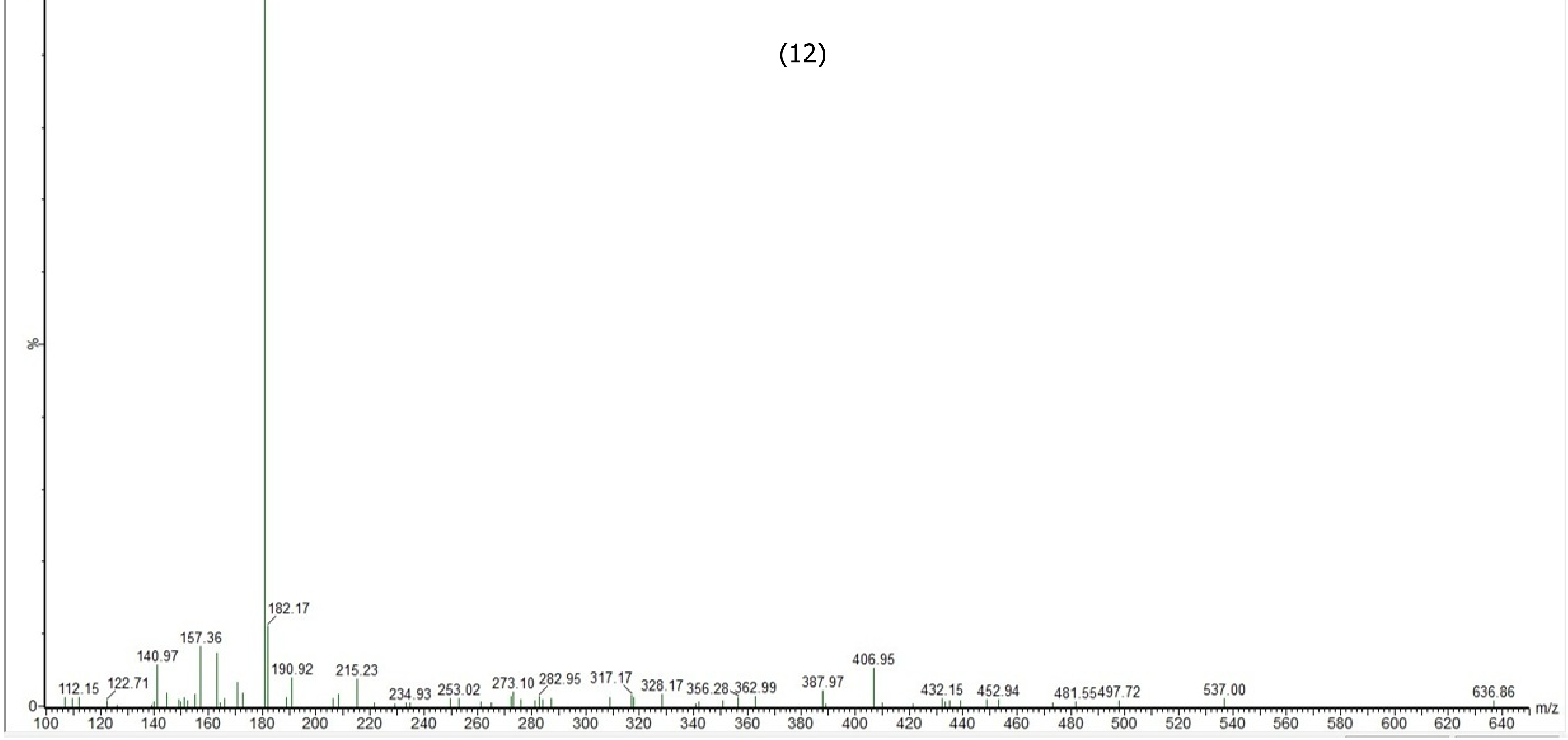

Figure S36. Mass Spectrum of caffeic acid (12)

m/z 163 [M-H]⁻

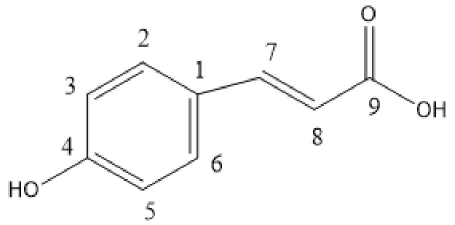

(13)

Chemical Formula: $C_9H_8O_3$
Exact Mass: 164.05

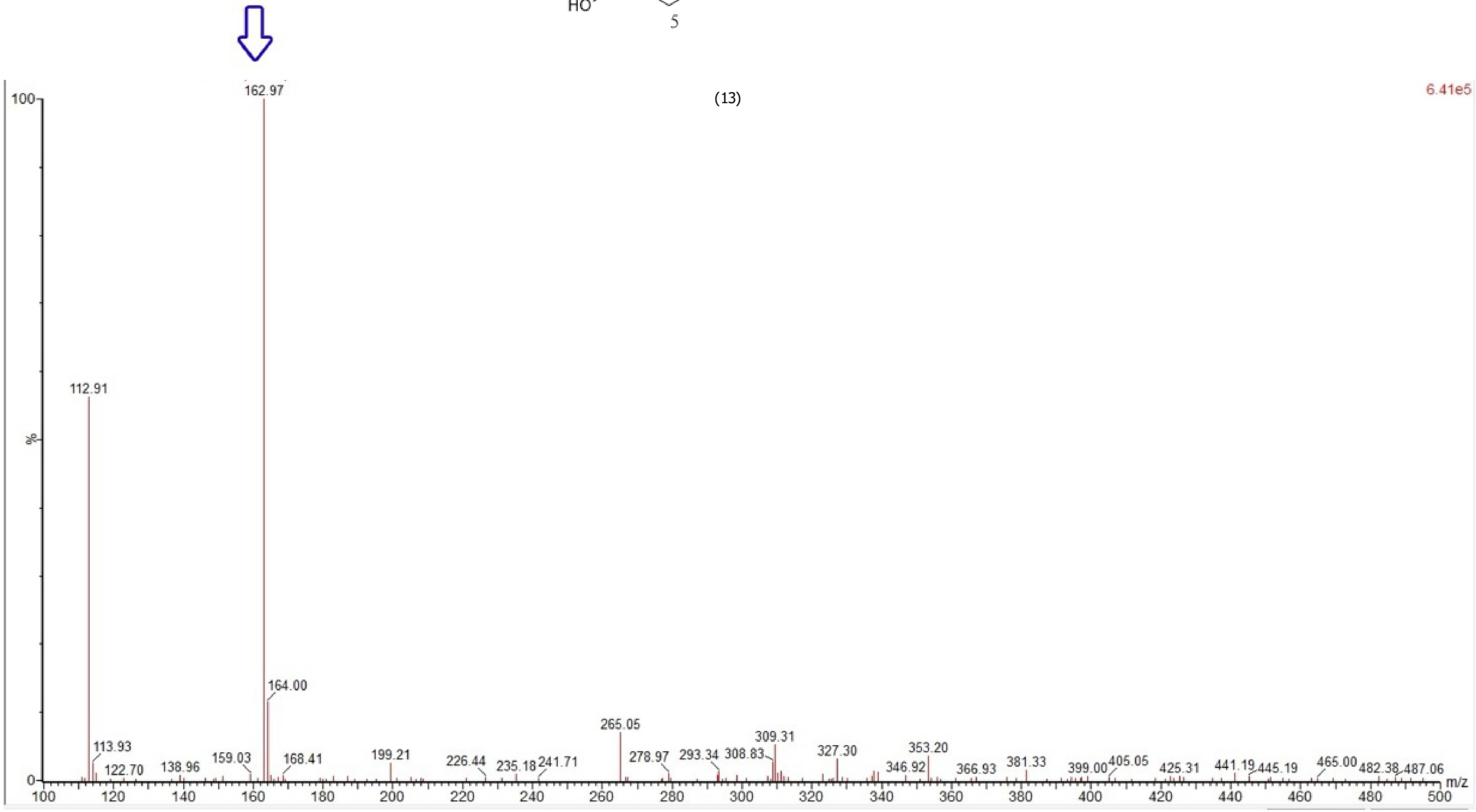

Figure S37. Mass Spectrum of coumaric acid (13)

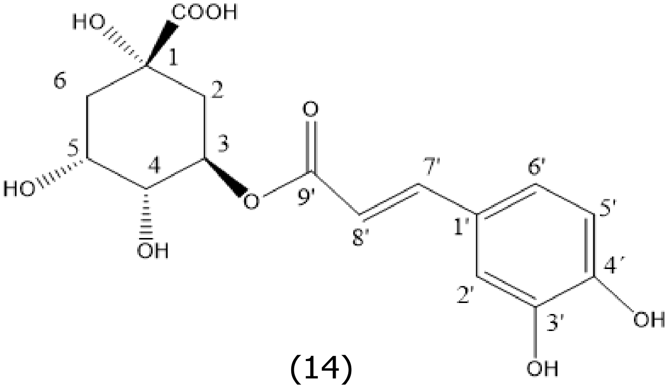

(14)

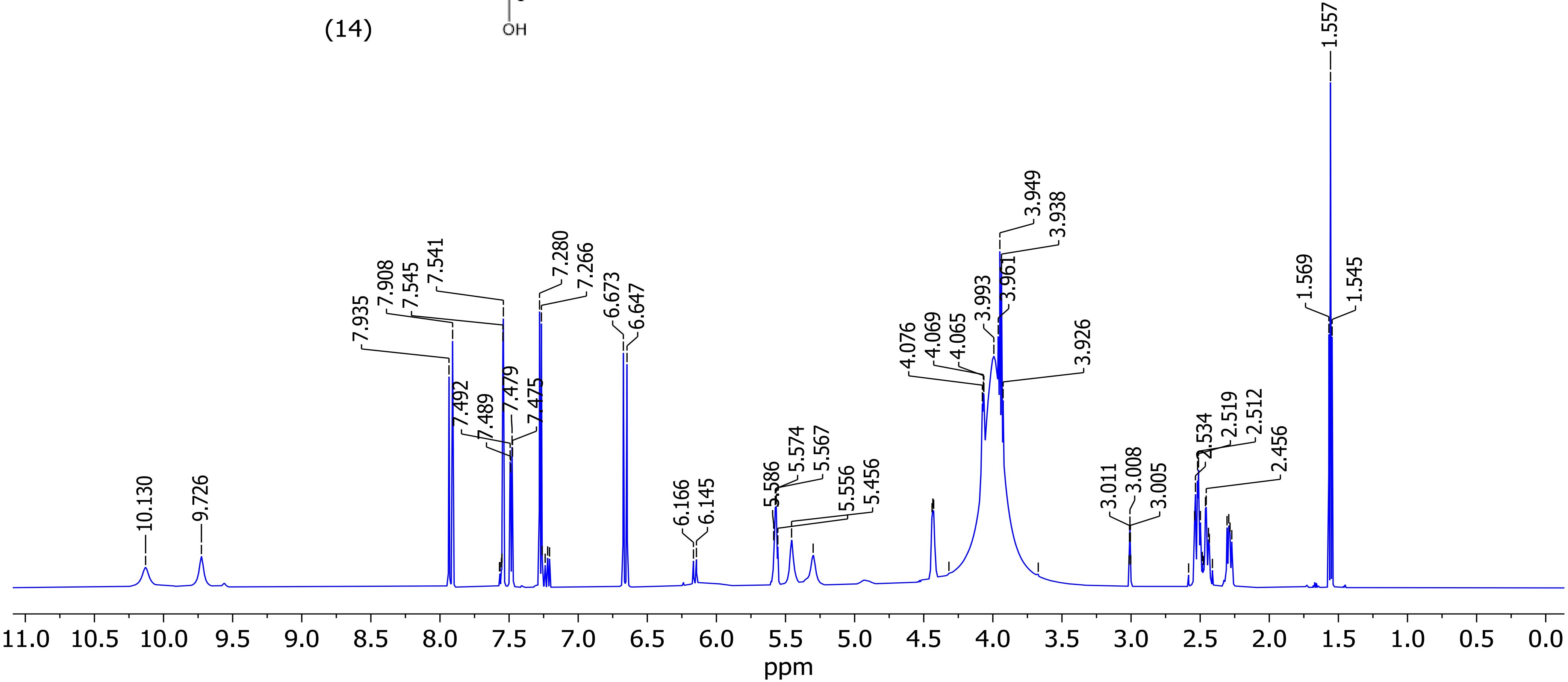

Figure S38. ^{1}H NMR (CD_3OD, 600 MHz) of chlorogenic acid (14)

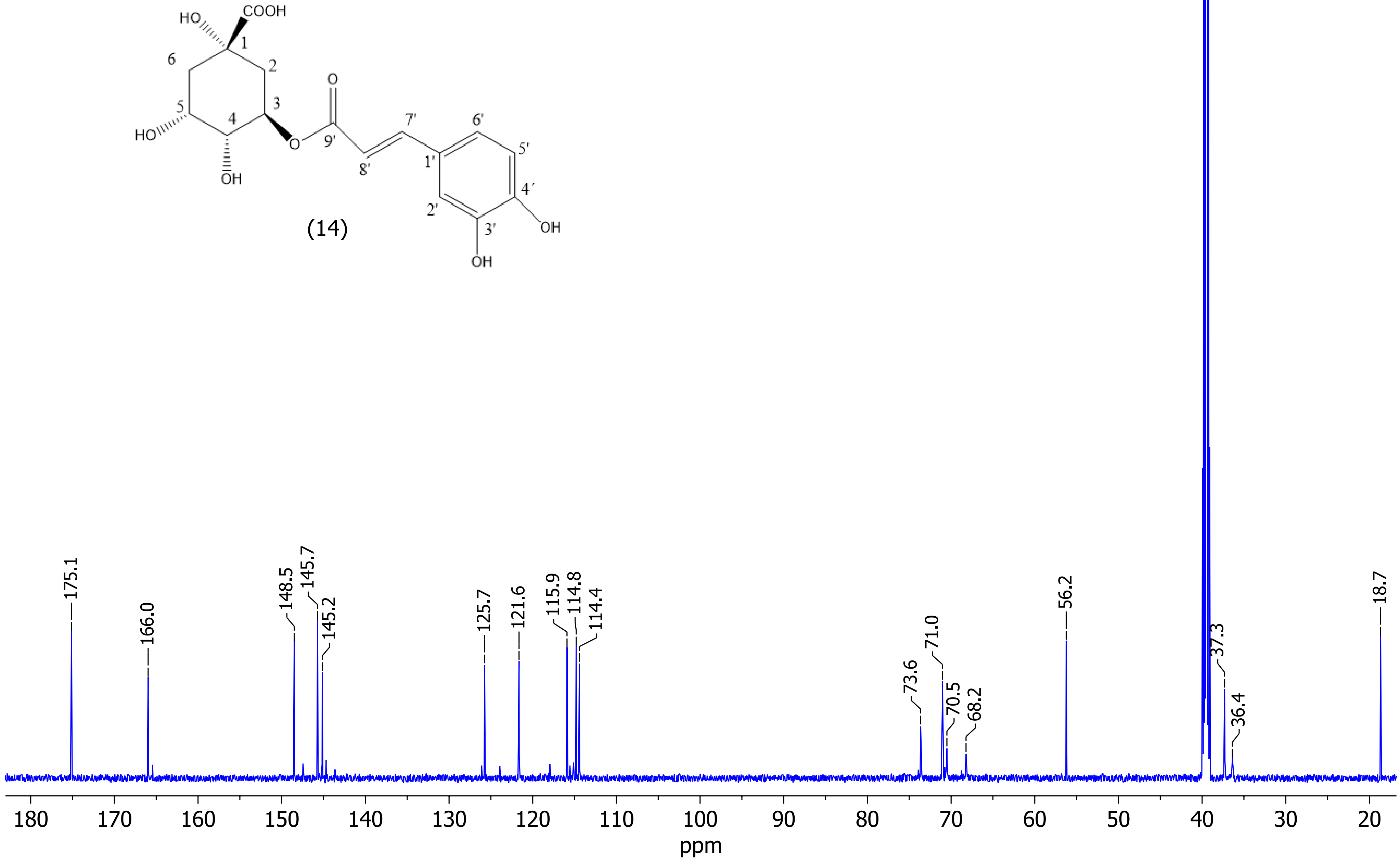

Figure S39. ^{13}C NMR (CD_3OD, 600 MHz) of chlorogenic acid (14)

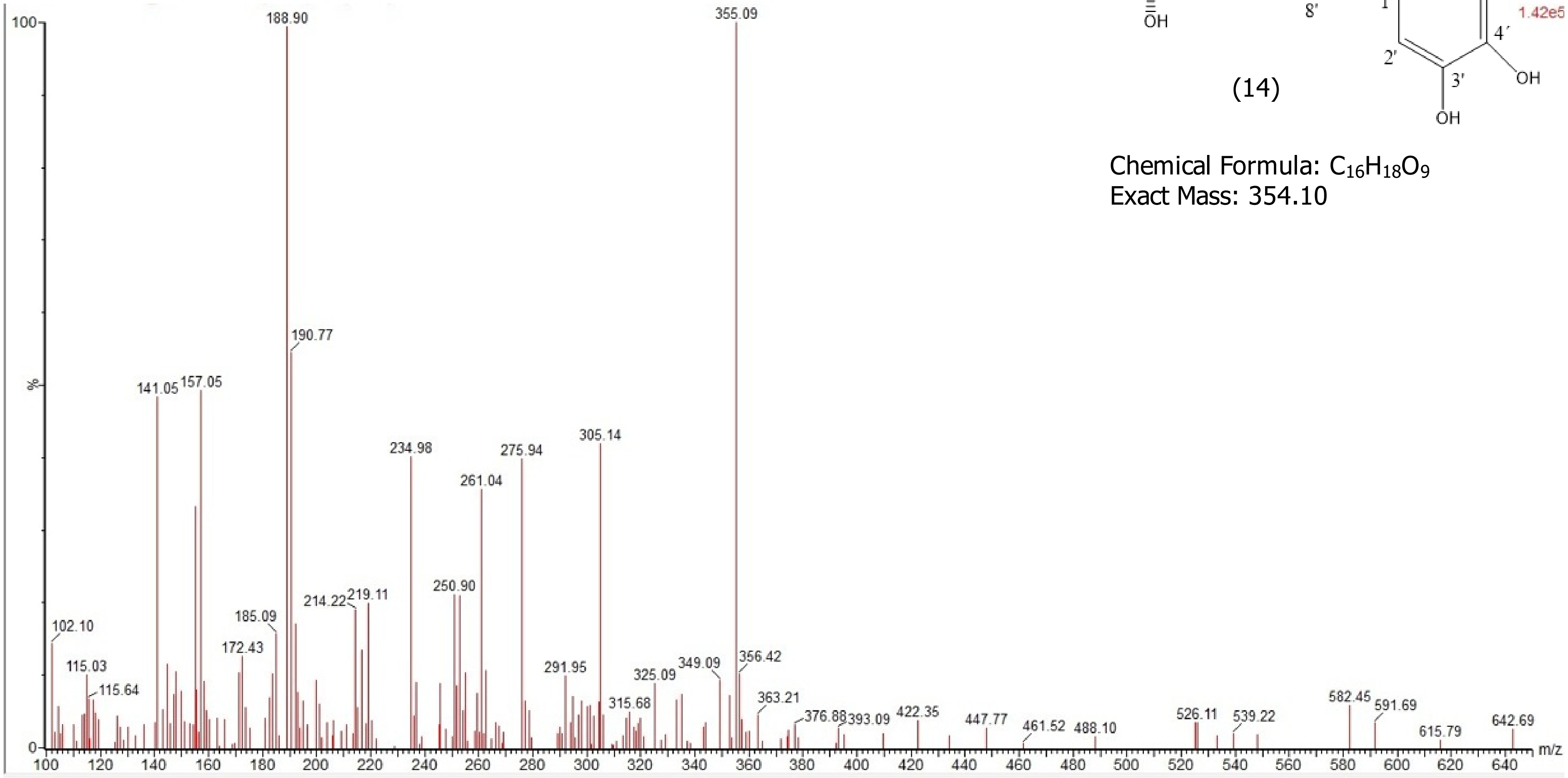

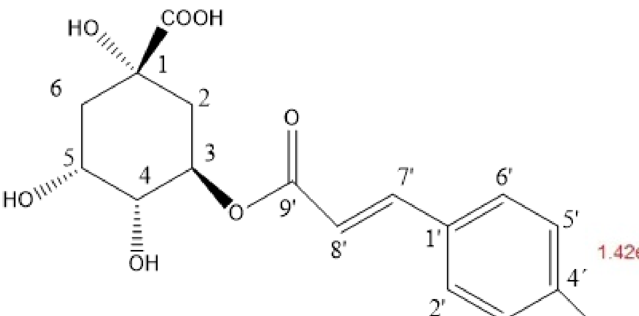

(14)

Chemical Formula: $C_{16}H_{18}O_9$
Exact Mass: 354.10

Figure S40. Mass ESspectrum of chlorogenic acid (14)

Table S1. 3D protein-ligand interaction diagrams of the active site of MAO-A and MAO-B with compounds isolated from *Tilia americana* var. *mexicana.*

Compound	MA0-A	MA0-B
α-Amyrine **(1)**	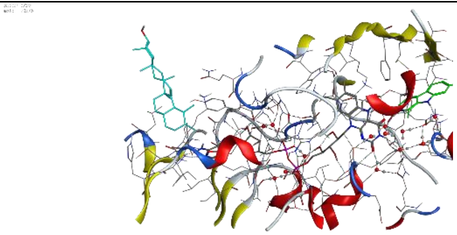	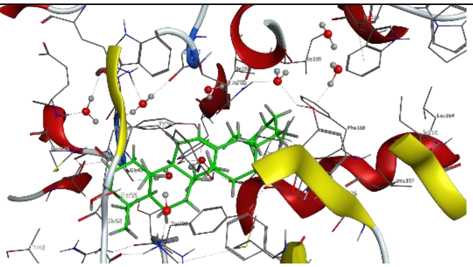
β-Amyrine **(2)**	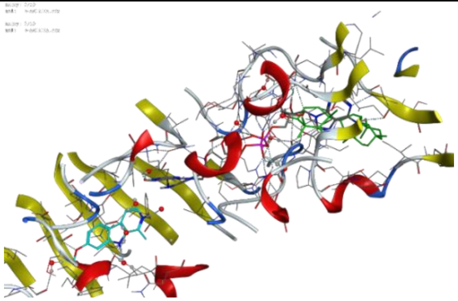	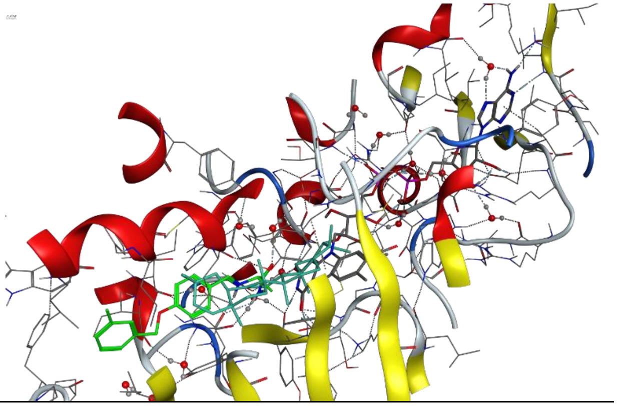

β-Sitosterol **(3)**	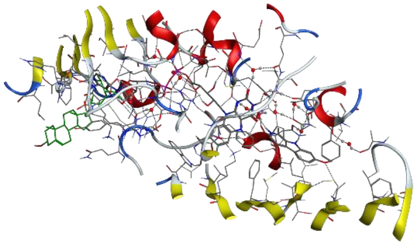	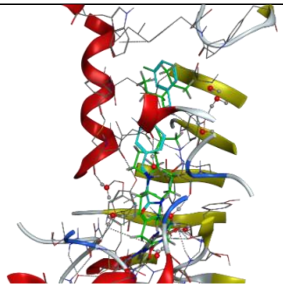
Stigmasterol **(4)**	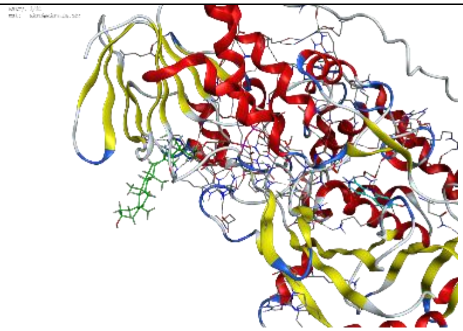	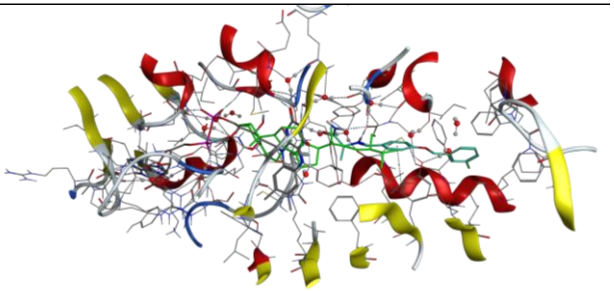
Ursolic acid **(5)**	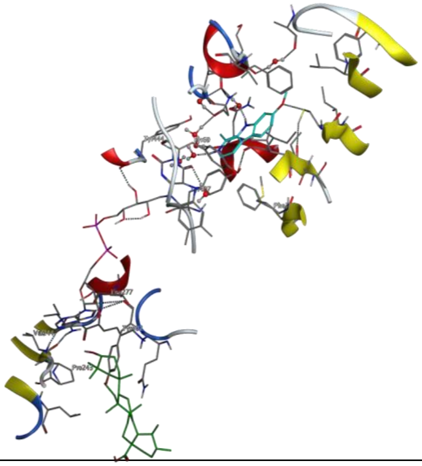	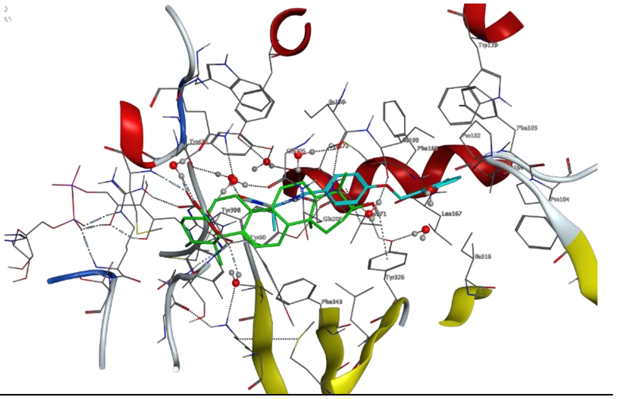

β-Sitosterol glycoside **(6)**	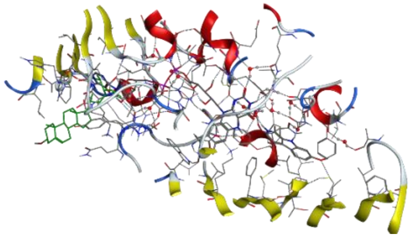	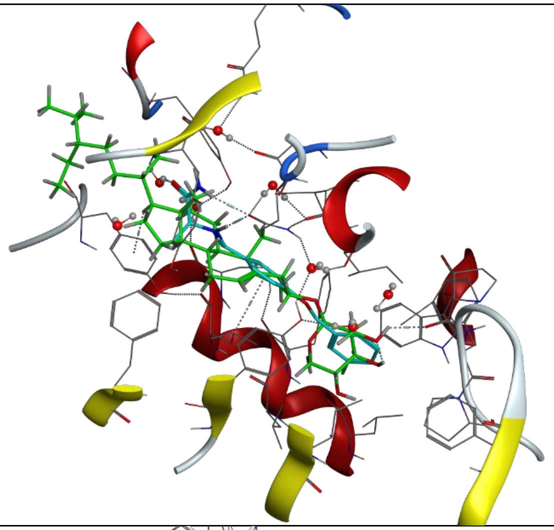
4α-Terpineol sambubioside **(7)**	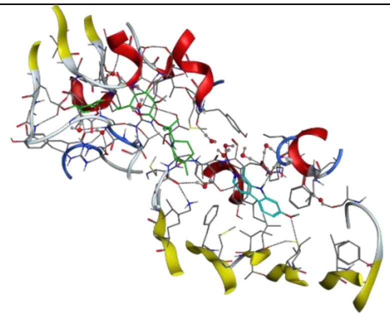	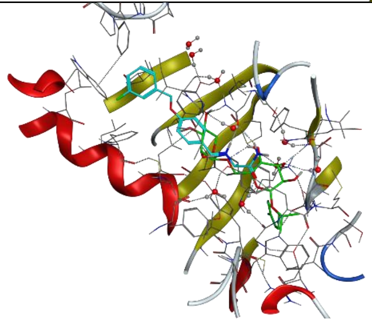
Tiliroside **(8)**	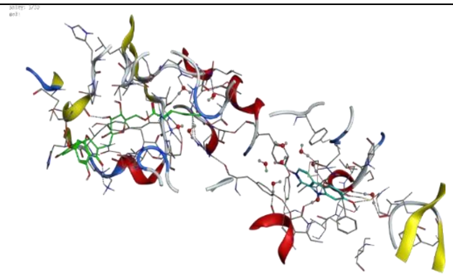	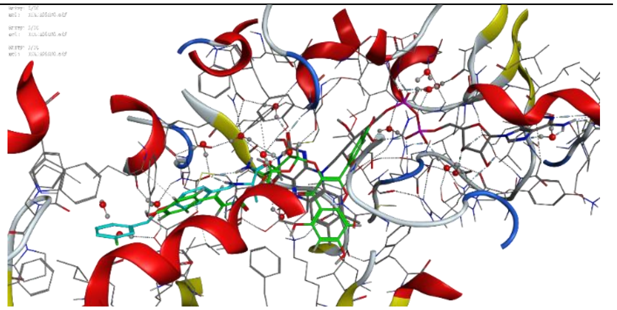

Rutin **(9)**	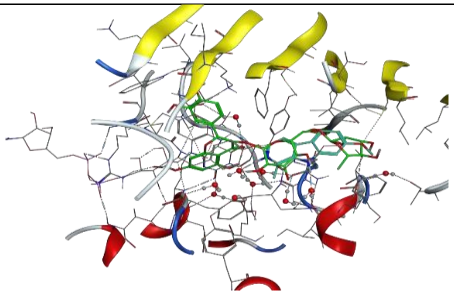	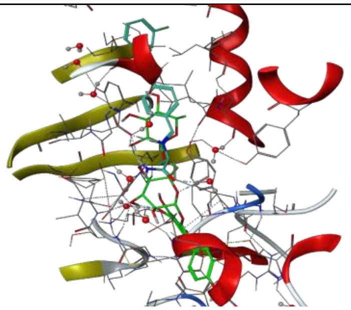
Scopoletin **(11)**	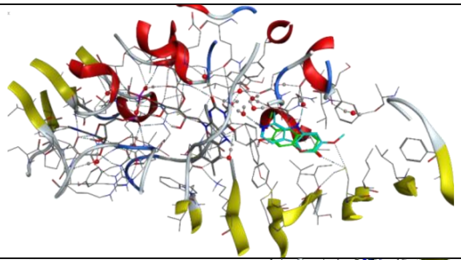	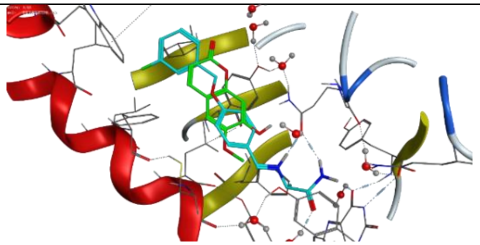
Caffeic acid **(12)**	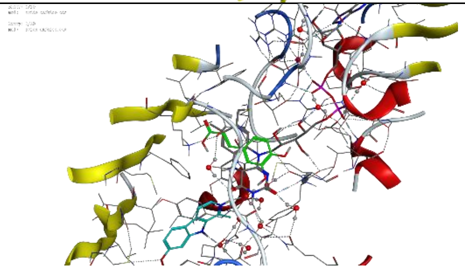	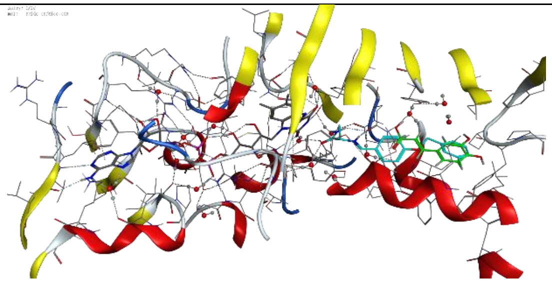
Coumaric acid **(13)**	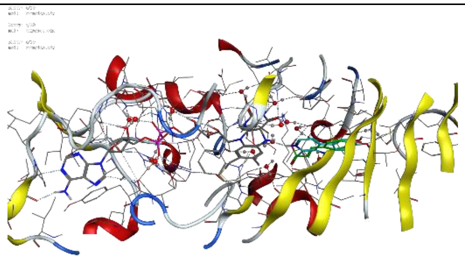	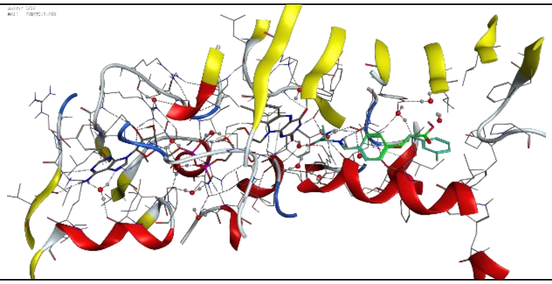

Chlorogenic acid **(14)**	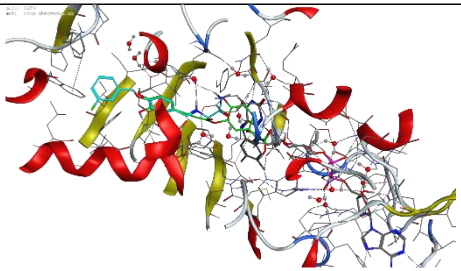	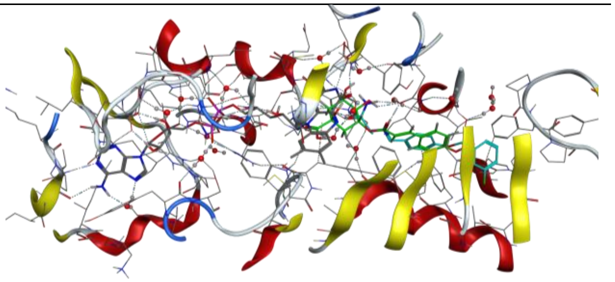

Table S2. Protein–ligand interaction diagrams of the active site 3D view of the binding interacting with MAO-B and 2D view of binding site interactions diagrams for compounds **(1)** - **(9)**; **(11)** - **(14)**.

<table>
<tr><th>Protein–ligand interaction diagrams of the active site 3D view of the binding interacting with MAO-B</th><th>2D view of binding site interactions diagrams
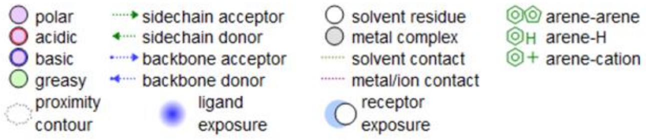
</th></tr>
<tr><td>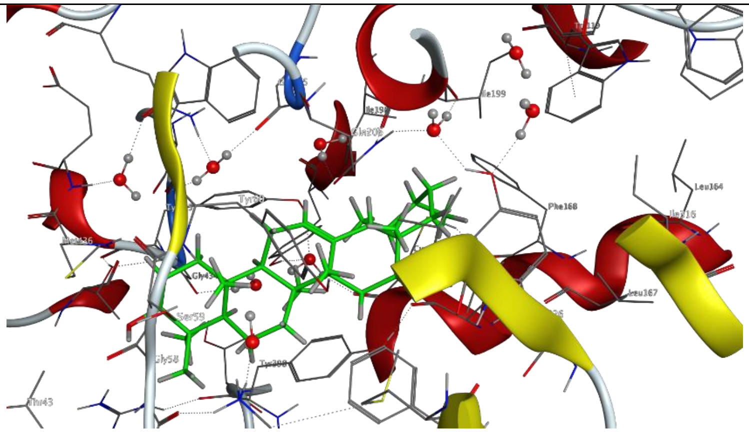

(1)</td><td>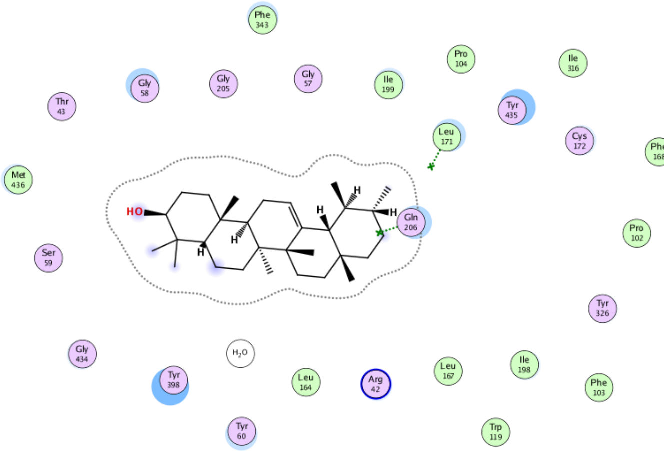

(1)</td></tr>
</table>

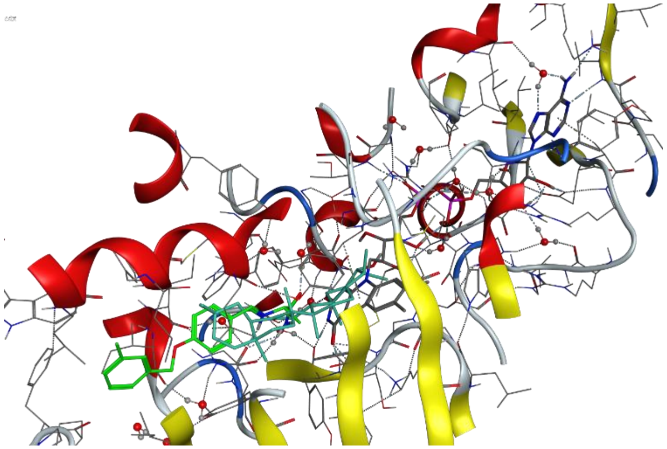

(2)

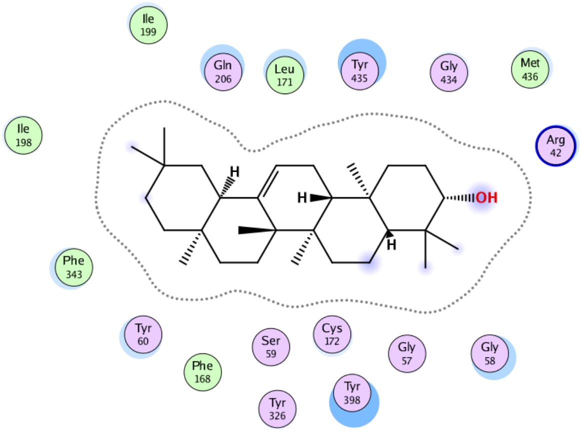
Ile 199
Gln 206
Leu 171
Tyr 435
Gly 434
Met 436
Ile 198
Arg 42
H
H
H
OH
Phe 343
Tyr 60
Ser 59
Cys 172
Gly 57
Gly 58
Phe 168
Tyr 398
Tyr 326

(2)

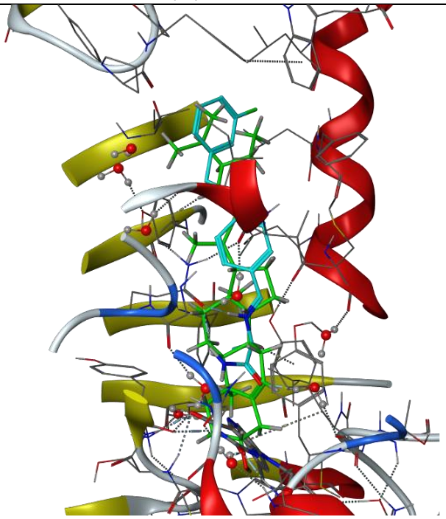

(3)

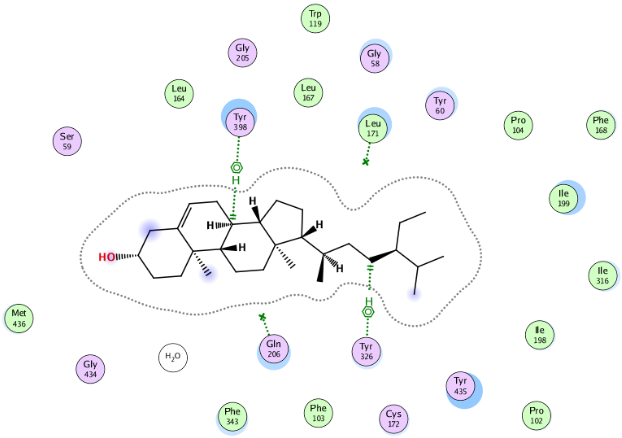
Trp 119
Gly 205
Gly 58
Leu 164
Leu 167
Tyr 398
Tyr 60
Leu 171
Ser 59
Pro 104
Phe 168
Ile 199
HO
Ile 316
Met 436
H_2O
Gln 206
Tyr 326
Ile 198
Gly 434
Tyr 435
Phe 343
Phe 103
Cys 172
Pro 102

(3)

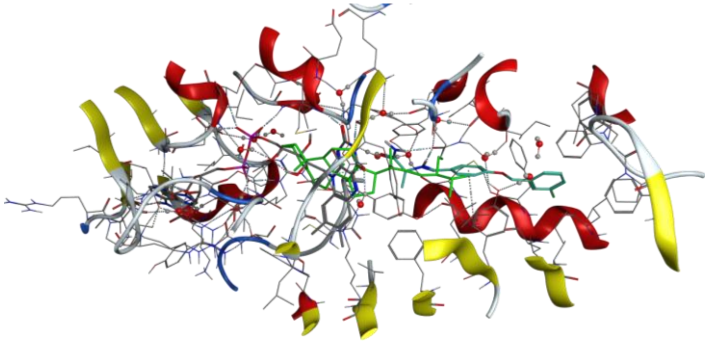

(4)

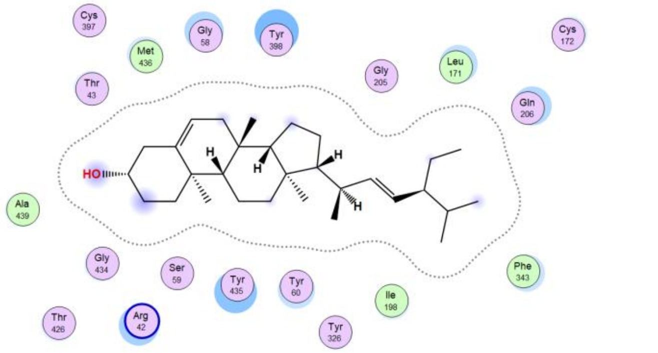
Cys 397
Met 436
Gly 58
Tyr 398
Cys 172
Thr 43
Gly 205
Leu 171
Gln 206
HO
Ala 439
Gly 434
Ser 59
Tyr 435
Tyr 60
Phe 343
Ile 198
Thr 426
Arg 42
Tyr 326

(4)

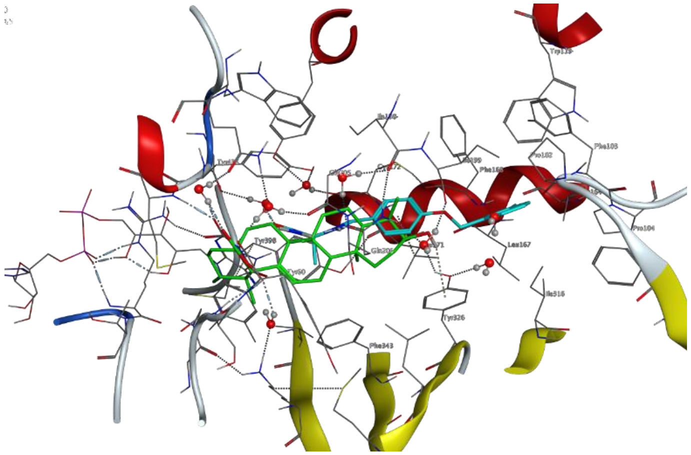

(5)

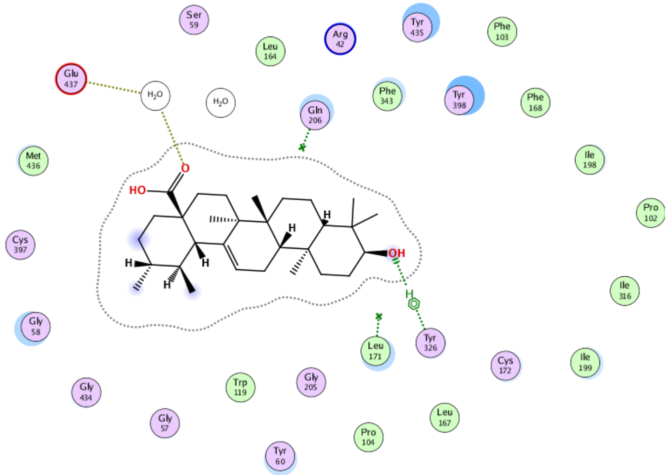
Ser 59
Tyr 435
Leu 164
Arg 42
Phe 103
Glu 437
H_2O
H_2O
Phe 343
Tyr 398
Phe 168
Gln 206
Met 436
Ile 198
HO
O
OH
Pro 102
Cys 397
Ile 316
Gly 58
Leu 171
Tyr 326
Cys 172
Ile 199
Gly 434
Trp 119
Gly 205
Leu 167
Gly 57
Pro 104
Tyr 60

(5)

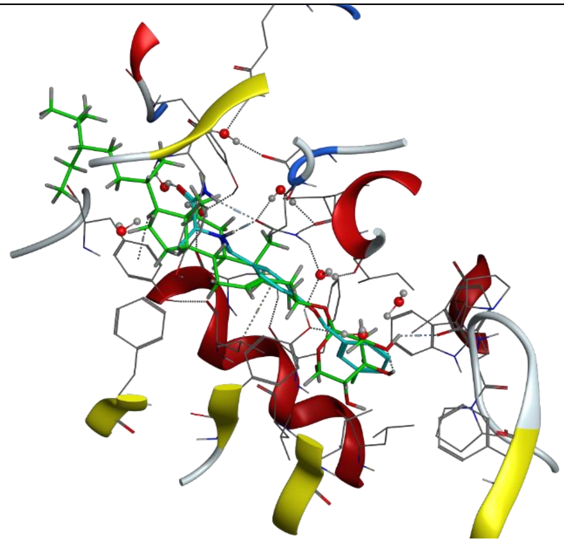

(6)

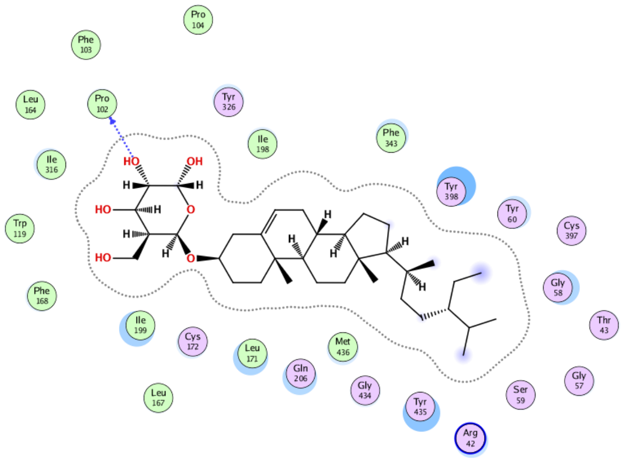
Pro 104
Phe 103
Leu 164
Pro 102
Tyr 326
Ile 198
Phe 343
Ile 316
Tyr 398
Tyr 60
Cys 397
Trp 119
Gly 58
Phe 168
Thr 43
Ile 199
Cys 172
Leu 171
Met 436
Gln 206
Gly 434
Tyr 435
Ser 59
Gly 57
Leu 167
Arg 42

(6)

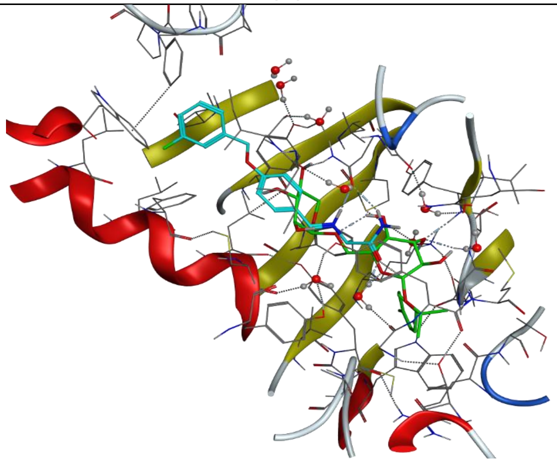

(7)

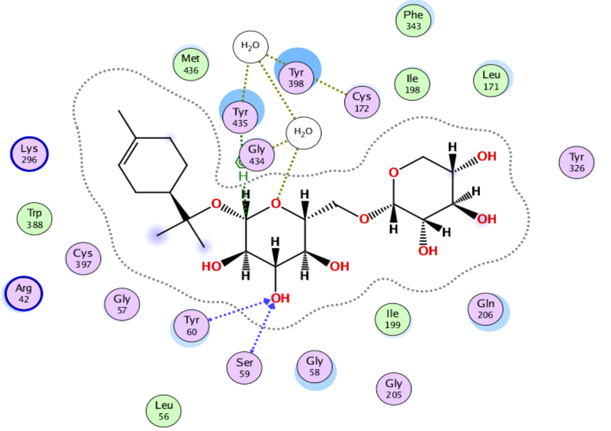
Phe 343
H_2O
Met 436
Tyr 398
Ile 198
Leu 171
Cys 172
Tyr 435
H_2O
Gly 434
Lys 296
Tyr 326
Trp 388
Cys 397
Arg 42
Gly 57
Tyr 60
Ser 59
Gly 58
Ile 199
Gln 206
Gly 205
Leu 56

(7)

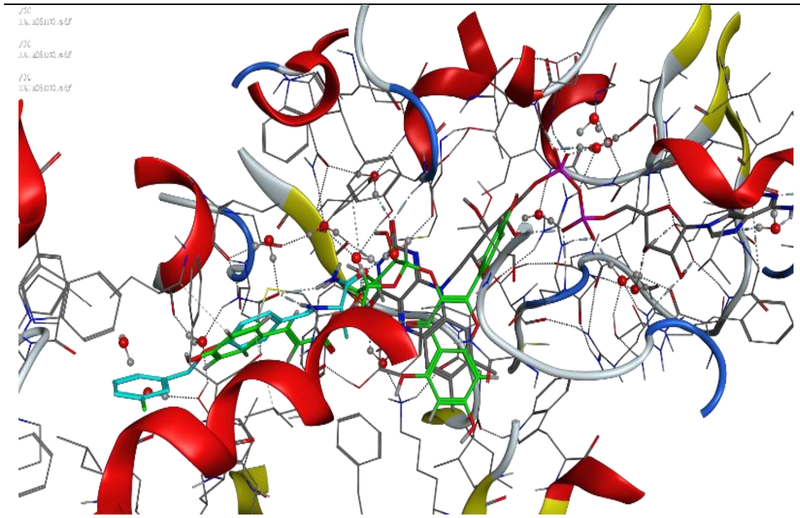

(8)

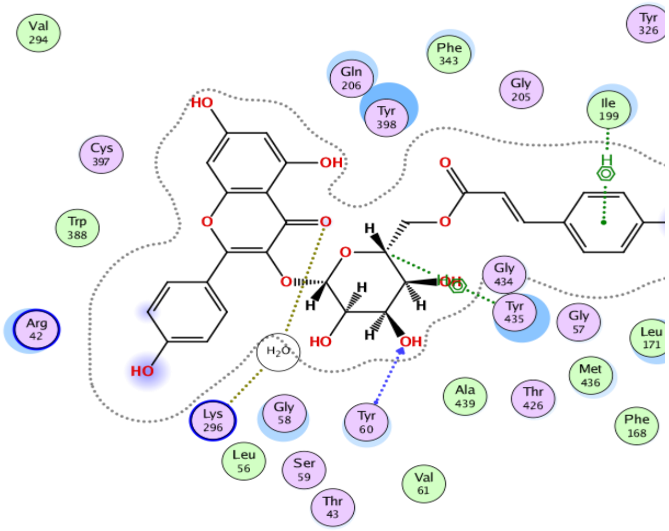

Val 294
Gln 206
Tyr 398
Phe 343
Gly 205
Ile 199
Tyr 326
Cys 397
Trp 388
Arg 42
Gly 434
Tyr 435
Gly 57
Leu 171
Lys 296
Gly 58
Tyr 60
Ala 439
Thr 426
Met 436
Phe 168
Leu 56
Ser 59
Thr 43
Val 61
HO
OH
HO

(8)

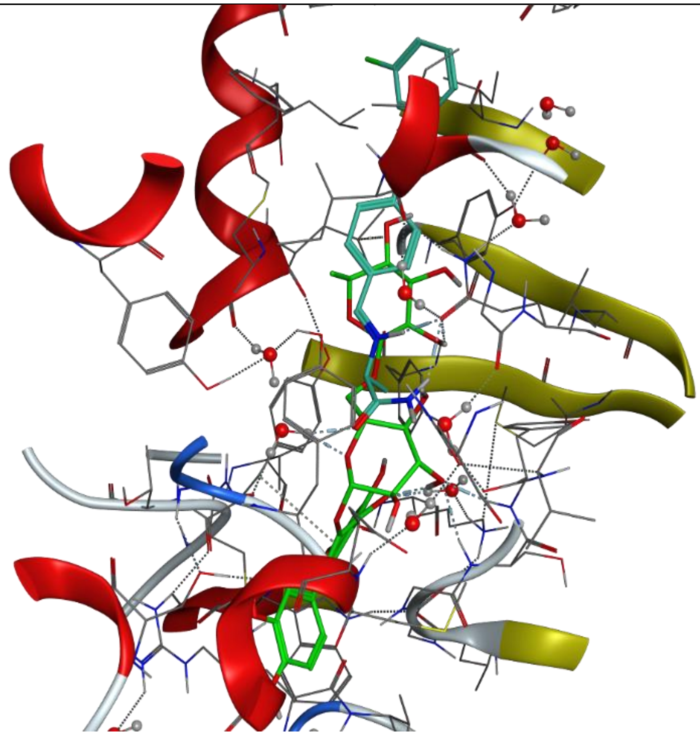

(9)

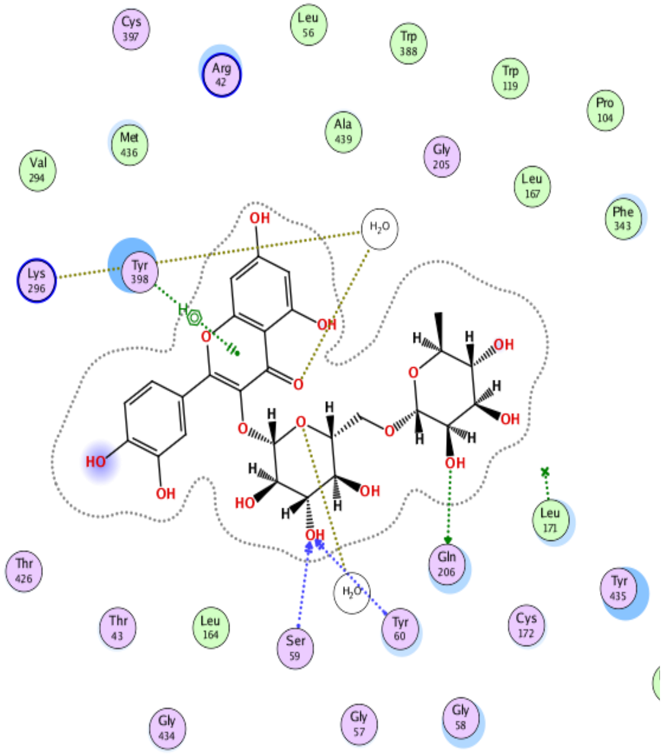
Cys 397
Leu 56
Trp 388
Arg 42
Trp 119
Pro 104
Met 436
Ala 439
Gly 205
Val 294
Leu 167
Phe 343
H2O
Lys 296
Tyr 398
OH
O
HO
Leu 171
Thr 426
Gln 206
Tyr 435
H2O
Thr 43
Leu 164
Ser 59
Tyr 60
Cys 172
Gly 434
Gly 57
Gly 58

(9)

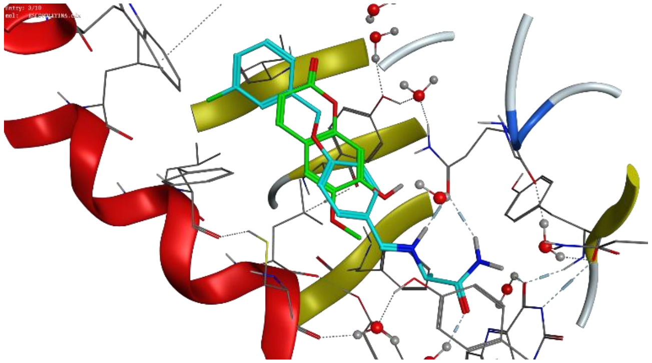

(11)

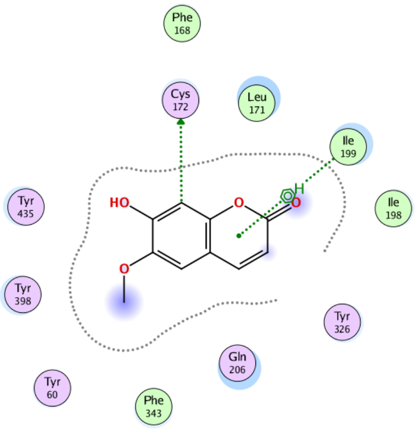
Phe 168
Cys 172
Leu 171
Ile 199
Tyr 435
HO
O
O
Ile 198
Tyr 398
Tyr 326
Gln 206
Tyr 60
Phe 343

(11)

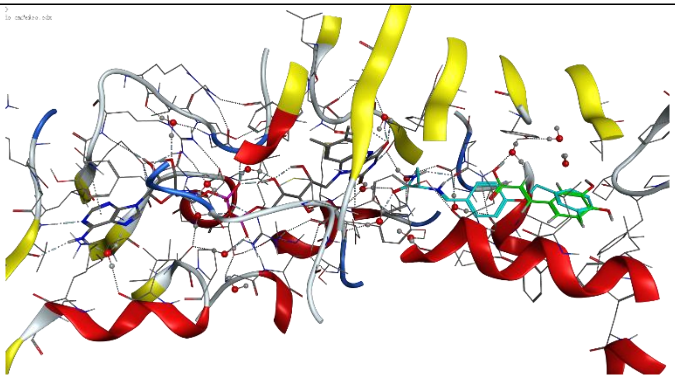

(12)

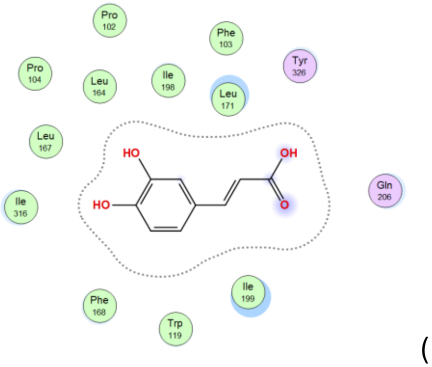
Pro 102
Phe 103
Pro 104
Leu 164
Ile 198
Leu 171
Tyr 326
Leu 167
HO
OH
HO
O
Ile 316
Gln 206
Phe 168
Trp 119
Ile 199

(12)

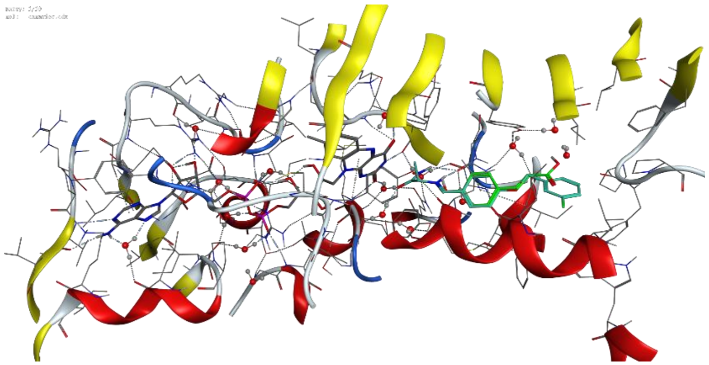

(13)

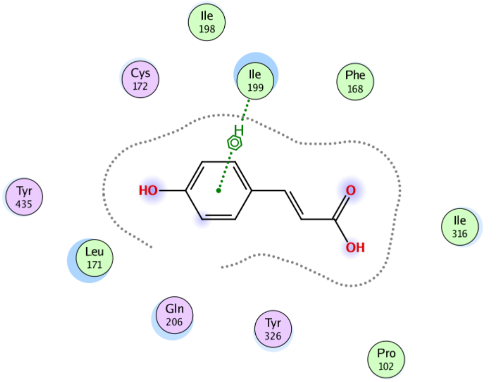
Ile 198
Cys 172
Ile 199
Phe 168
Tyr 435
HO
O
OH
Ile 316
Leu 171
Gln 206
Tyr 326
Pro 102

(13)

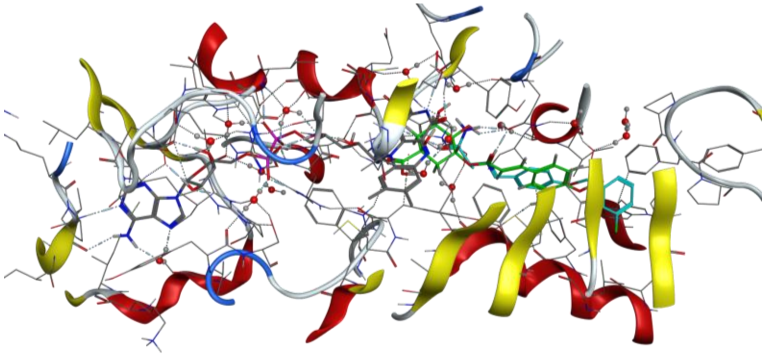

(14)

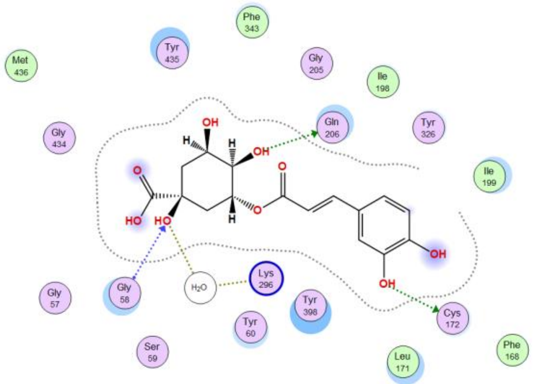
Phe 343
Met 436
Tyr 435
Gly 205
Ile 198
Gly 434
Gln 206
Tyr 326
Ile 199
OH
OH
O
HO
HO
OH
OH
H₂O
Lys 296
Gly 58
Gly 57
Tyr 398
Tyr 60
Ser 59
Cys 172
Leu 171
Phe 168

(14)

Table S3. Interaction/Amino Acid with MAO-B diagrams for compounds from *Tilia americana* var. *mexicana*

Compound	Interaction/Amino Acid with MAO-B
**Safinamide (Reference compound)**	**Polar:** Cys397, Gly57, Cyst172, Gly58.<br>**Greasy:** Trp388, Phe343, Ile198, Phe168, Leu56,<br>Basic: Arg42, Lys296<br>**Receptor exposure and polar:** Tyr398, Gly,58, Tyr435, Gln206<br>**Receptor exposure and greasy:** Ile199, Leu171, Phe 343<br>**Side chain acceptor:** Cys172<br>**Backbone chain acceptor:** Ile198<br>**Arene-H:** Leu171
**α-amyrine (1)**	**Greasy:** Val244, Pro243<br>**Receptor exposure and polar**: Tyr402, Gln401, Thr245<br>**Receptor exposure and greasy:** Leu259<br>**Receptor exposure and basic:** Lys280
**β--amirine (2)**	**Polar:** Gly434, Gly57, Cys172, Ser59, Tyr326.<br>**Basic:** Arg42.<br>**Greasy:** Ile198, Ile199, Met436, Phe168.<br>**Receptor exposure and polar :** Gln206, Tyr435, Tyr60, Tyr398, Gly58.

**β-sitosterol (3)**	**Polar:** Ser59, Gl205, Gly434, Cys172, Tyr326.<br>**Greasy:** Met436, Phe103, Pro102, Ile198, Ile316, Phe168, Pro104, Trp119, Leu167, Leu164.<br>**Receptor exposure and polar:**Tyr 398, Gly 58, Tyr 60, Tyr 432, Gln206.<br>**Receptor exposure and greasy:** Leu171, Ile199.<br>**Arene-H:** Tyr326, Tyr398.<br>**Side chain acceptor:** Leu171, Gln206.
**Stigmasterol (4)**	**Polar: Cys397, Thr43, Gly205, Cys172, Gly434, Ser59, Tyr326**<br>**Greasy**: Ala439, Ile198, Met436<br>**Basic:** Arg42<br>**Receptor exposure and polar: Gly58, Tyr398, Gln206, Tyr60, Tyr435**<br>**Receptor exposure and greasy:** Leu171, Phe343<br>**Receptor exposure and basic:** Arg42.
**Ursolic acid (5)**	**Polar:** Ser59, Cys172, Tyr326, Gly205, Gly57, Gly434, Cys397.<br>**Greasy:** Met436, Leu164, Phe343, Phe103, Phe168, Ile198, Pro102, Ile316, Ile99, Leu167, Pro104, Trp119.<br>**Basic:** Arg42.<br>**Acidic:** Glu437.<br>**Receptor exposure and polar:** Gly58, Gln206, Tyr435, Tyr298, Tyr60.<br>**Receptor exposure and greasy:** Leu171.<br>**Side chain donator:** Gln206, Leu171.<br>**Pi-arene:** Tyr326.<br>**Solvent contact:** Glu437.
**β-sitosterol glucoside (6)**	**Polar:** Tyr 326, Cys172, Gly434, Ser59, Gly57, Thr43, Cys397, Tyr60.<br>**Greasy:** Pro102, Pro103, Pro 104, Leu164, Ile316, Trp119, Phe168, Leu167, Met436.<br>**Basic:** Arg42.<br>**Receptor exposure and polar:** Gln206, Tyr435, Gly58, Tyr398.<br>**Receptor exposure and greasy: Ile199**, Leu171.<br>**Backbone donor:** Pro102

**4α –terpineol sambubioside (7)**	**Polar:** Cys397, Gly57, Ser59, Gly205, Gly434, Cys172, Tyr326.<br>**Greasy:** Trp388, Leu56, Ile199, Leu171, Ile198, Met436.<br>**Basic:** Arg42, Lys296.<br>**Receptor exposure and polar:** Tyr435, Tyr398, Tyr60, Gly58, Gln206.<br>**Solvent contact:** Gly434, Tyr435, Tir398, Cys172.<br>**Backbone donor:** Tyr60, Ser59.
**Tiliroside (8)**	**Polar:** Cys397, Tyr326, Gly205, Cys172, Gly57, Thr426, Ser59, Thr43.<br>**Greasy:** Val 294, Trp388, Leu56, Val61, Ala439, Met436, Phe168, Ile198.<br>**Receptor exposure and Basic:** Lys 296.<br>**Receptor exposure and polar:** Gln205, Tyr398, Tyr435, Tyr60, Gly58.<br>**Receptor exposure and greasy:** Ile199, Leu171.<br>**Backbone donor:** Tyr60.<br>**Solvent contact:** Lys296.<br>**Arene-H:** Ile199.
**Rutin (9)**	**Polar:** Cys397, Gly205, Tyr326, Thr426, Thr43, Gly434, Ser59, Gly57, Cys172<br>**Greasy:** Val294, Met436, Ala439, Leu56, Trp388, Trp119, Leu167, Pro104, phe343, Ile199, Pro102, Ile 198, Phe 103, Leu 164<br>**Receptor exposure and polar:** Tyr398, Gln206, Tyr435, Gly58.<br>**Receptor exposure and basic:** Tyr398<br>**Arene-H Bond:** Tyr398<br>**Solvent residues:** Tyr398, Lys296.<br>**Backbone donor:** Ser59, Tyr60.
**Scopoletin (11)**	**Polar:** Cys172, Tyr435, Tyr398, Tyr60, Tyr326.<br>**Greasy:** Phe168, Ile198, Phe343.<br>**Receptor exposure and polar:** Gln206.<br>**Receptor exposure and greasy:** Leu171, Ile199.<br>**Arene-H bond:** Ile199

**Caffeic acid (12)**	**Polar:** Tyr326, Gln2026<br>**Greasy:** Pro104, Pro102, Leu167, Leu168, Ile 198, Phe 103, Trp119, Phe168, Ile313.<br>**Receptor exposure and polar:** Gln206.<br>**Receptor exposure and greasy:** Leu171, Ile199.
**Coumaric acid (13)**	Polar: Cys172, Tyr435, Tyr326.<br>Greasy: Ile198, Phe168, Ile316, Pro102.<br>**Receptor exposure and polar:** Gln206.<br>**Receptor exposure and greasy:** Ile199, Leu171.<br>**Arene-H interaction:** Ile199.
**Chlorogenic acid (14)**	**Polar: Gly434, Gly205, Tyr326,Ser59, Gly57**<br>**Greasy:** Met436, Phe343,Ile198, Phe168<br>**Receptor exposure and polar: Tyr435, Gln206, Ty 398, Ggly58**<br>**Receptor exposure and greasy: Ile199, Leu171,**<br>**Polar and basic: Lys296**<br>**Back bone acceptor:** Gly58
